# Ligand modification for the tuning of activity and selectivity in the chemoselective transfer hydrogenation of α,β-unsaturated carbonyls using EtOH as a hydrogen source

**DOI:** 10.1039/d5dt01348h

**Published:** 2025-07-28

**Authors:** Alicia Beaufils, Nicole Elia, Sabela Reuge, Martin Albrecht

**Affiliations:** a Department of Chemistry, Biochemistry, and Pharmaceutical Sciences, University of Bern Freiestrasse 3 CH-3012 Bern Switzerland martin.albrecht@unibe.ch

## Abstract

The selective reduction of α,β-unsaturated ketones, either at the olefinic or the carbonyl site, offers attractive synthetic opportunities. While carbonyl reduction is well established, selective olefin reduction is less common, particularly when using environmentally friendly ethanol as a hydrogen source. Recently, we reported a coordinatively unsaturated ruthenium complex containing an *N*,*N*′-bidentate coordinating pyridinium amidate (PYA) ligand as an efficient catalyst for ethanol-based transfer hydrogenation of α,β-unsaturated ketones; however, there was over-reduction and thus loss of selectivity in reactions over an extended period of time. Capitalizing on the facile synthetic modulation of PYA ligands, we herein report on a series of operationally unsaturated two-legged piano-stool ruthenium cymene complexes [Ru(N^N′)(cym)](PF_6_) 3a–e with modifications on the PYA-appended aroyl unit. Spectroscopic analysis of these complexes suggests a higher contribution of the π-basic zwitterionic resonance structure of the PYA unit in CD_2_Cl_2_ and a larger contribution of the π-acidic quinoidal structure in polar and more coordinating CD_3_OD. The latter also allows for stabilization of the catalytically relevant alkoxide intermediate [Ru(OEt)(N^N′)(cym)] 4. Application of complexes 3a–3e in transfer hydrogenation of *trans*-chalcone indicates generally good transfer hydrogenation activity and good selectivity towards olefin hydrogenation for all complexes. The variant with a *p*-CF_3_-C_6_H_4_ substituted PYA ligand, complex 3c, combined high activity and very high selectivity, affording almost exclusively the desired saturated ketone product with only traces of the saturated alcohol even after prolonged reaction times, underpinning the effectiveness of PYA ligand modulation in tailoring activity and selectivity.

## Abstract 2

*N*,*N*′-bidentate pyridinium amidate (PYA) ruthenium complexes catalyze the selective olefin transfer hydrogenation of α,β-unsaturated ketones in ethanol. Tailoring the aryl substituent enhances the activity and selectivity toward olefin hydrogenation.

## Graphical abstract

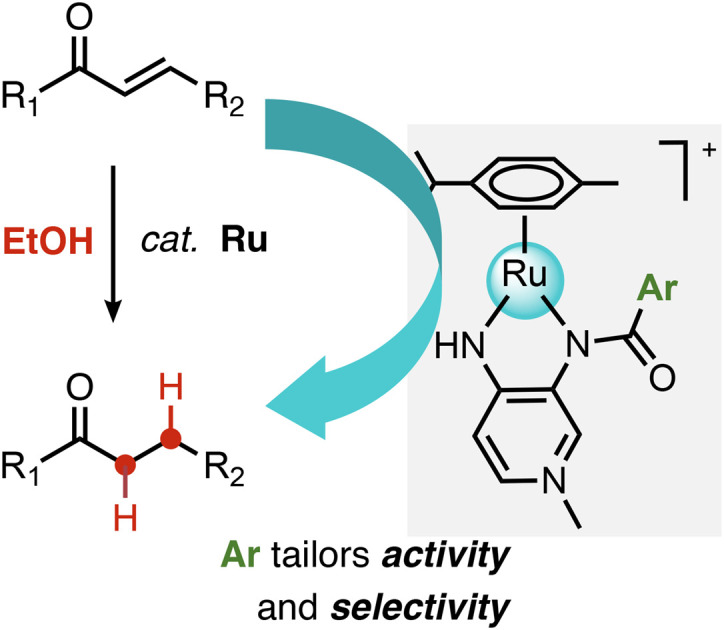

## Introduction

Metal-mediated catalysis relies on the availability of a free coordination site at the metal center for substrate coordination.^1–3^ In homogeneous catalysis, such a free site can be introduced by various methodologies, including the cleavage of dimeric complexes or the selective abstraction of a coordinated ligand. The latter method often involves solvent coordination,^4^ and thus ligand abstraction typically results in the replacement of a kinetically robust ligand by a kinetically labile one. In some cases, however, coordinative unsaturation is sufficiently stable for ‘under-ligated’ complexes to be isolated,^5–8^ with obvious opportunities for catalysis. Closer inspection of such coordinatively unsaturated complexes indicated that one of the ligands tends to compensate for the unsaturation by π-donor interactions, which leads to partial electronic saturation.^9–11^ As a consequence, the term ‘operationally unsaturated’ has been proposed to characterize these complexes.^8,11,12^

**Fig. 1:**
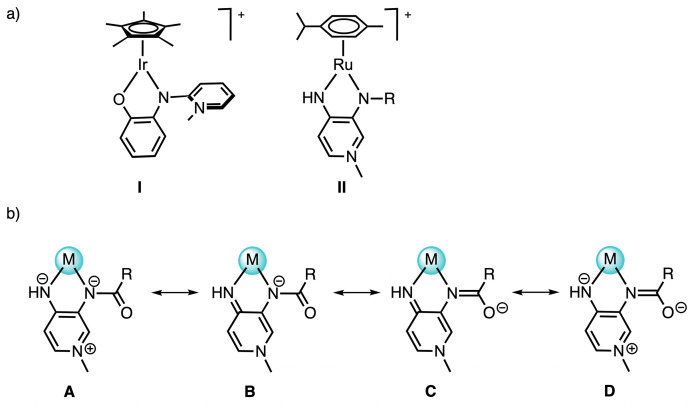
(a) Underligated Ir^III^ and Ru^II^ complexes containing pyridinium amidate (PYA) ligands; (b) schematic representation of the limiting resonance structures in *N*,*N*′-bidentate PYA complexes.

We recently demonstrated that functionalized and bidentate coordinating pyridinium amidate (PYA) ligands^13^ readily form operationally unsaturated complexes of iridium(iii) and ruthenium(ii), leading to compounds with two-legged piano-stool geometries such as I and II (Fig. 1a).^14–17^ These complexes are sufficiently stable to be isolated in the solid state and for investigation of their reactivity towards exogenous ligands and substrates for catalytic transformations.^16,17^ For example, the PYA iridium complex I is a catalyst for formic acid dehydrogenation with outstanding activity,^14^ while ruthenium complex II efficiently catalyzes the transfer hydrogenation of α,β-unsaturated ketones under mild conditions using ethanol as a renewable and attractive hydrogen source.^18^ Despite their high efficiency, the hydrogen transfer leads to gradual over-reduction of the C=O bond after complete transformation of the olefinic C=C bond, thus limiting its applicability since quenching of the reaction at full conversion is necessary to maintain high selectivity. This limitation prompted us to investigate the implication of the ligand system in order to suppress the undesired carbonyl reduction reactivity.

Among the PYA ligands reported over the years,^13,19–24^ one influential variation on the ligand scaffold pertains to the acyl unit. For example, carbonyl substituents incorporating donor motifs such as pyridine, N-heterocyclic carbene, or phenyl groups as chelating ligands have been developed.^25–28^ Taking advantage of the specific *N*,*N*′-coordination of the ligand and the easy modification of PYAs,^13^ we describe here the synthesis and characterization of a series of *N*,*N*′-bidentate PYA ruthenium complexes derived from II with modified acyl units. The flexible donor properties of the *N*,*N*′-bidentate PYA unit are represented by the various limiting resonance structures including (i) coordination as a formally dianionic π-basic ligand with remote pyridinium stabilization (resonance structure A, Fig. 1b) and (ii) as a formally monoanionic ligand with a π-acidic ligand imine-type coordination site (resonance structures B and D). In addition, we envisioned that the acyl substituent R further affects the electronic properties of the metal center, with the possibility to introduce two formally neutral imine bonding sites and a remote oxoanionic feature as shown in the limiting resonance structure C. Such electronic modulation in the acyl unit was previously demonstrated to affect metal coordination with first-row pincer-type PYA complexes, though with a change in the amide coordination mode from nitrogen (κ-N) to oxygen (κ-O).^29^ Furthermore, steric effects at this position are expected to influence the accessibility of the metal center by the substrate, thus impacting both activity and selectivity, a common challenge for transfer hydrogenation of α,β-unsaturated ketones.^30–33^ Here we have explored the impact of systematic acyl modifications on the catalytic application of *N*,*N*-bidentate PYA ruthenium complexes in transfer hydrogenation using ethanol as a hydrogen source. Specifically, we demonstrate that specific variations of the acyl unit enhance the selectivity towards olefin reduction considerably, even in reactions that were run over extended periods of time.

## Results and discussion

### Synthesis and characterization of the second generation of *N*,*N*′-bidentate PYA ruthenium complexes

**Scheme 1:**
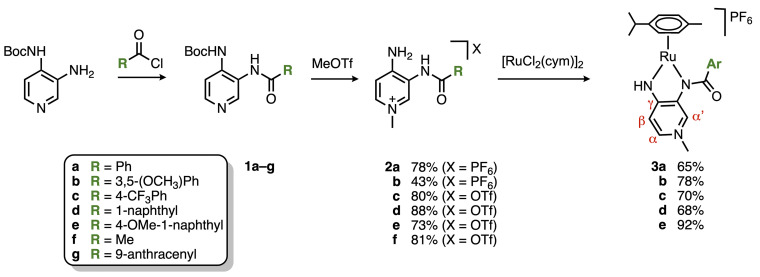
Synthesis of ligands and ruthenium complexes 3a–e.

The ligand precursor pyridinium salts were synthesized according to previously reported procedures,^16^ starting from *tert*-butyl(3-amino-pyridin-4-yl)carbamate and substituted acyl chlorides (Scheme 1). The acyl chlorides were commercially available except for 4-methoxy-1-naphthoyl chloride, which was synthesized in quantitative yield from the corresponding carboxylic acid in the presence of an excess of SOCl_2_. Amidation of these acyl chlorides under basic conditions yielded compounds 1a–e in acceptable 54–90% yields. In the presence of 5 eq. of MeOTf, selective methylation of the pyridine nitrogen accompanied by Boc-deprotection of the 4-amino sites yielded the corresponding pyridinium salts 2a–e. Direct anion exchange from OTf^−^ to PF_6_^−^ was performed for complexes 2a and 2b and was accomplished with an excess of NH_4_PF_6_ in a MeCN/H_2_O mixture. While the triflate salt of 2b was isolated as an oil, the PF_6_^−^ analogue was obtained as a solid, which facilitated purification considerably. An attempt to expand the aryl substituent to anthracyl groups failed because methylation of 1g gave only decomposition products irrespective of the methylating agent (MeOTf or MeI) or the reaction conditions (25–40 °C, CH_3_CN or CH_2_Cl_2_ as the solvent, and 1–5 eq. of MeX) and yielded the starting compound pyridine-3,4-diamine as the major product. Salts 2a–b were ruthenated in the presence of [RuCl_2_(cym)]_2_ (cym = *p*-cymene) and NaOAc in CH_2_Cl_2_ under reflux conditions, with the addition of NaPF_6_ for salts 2c–e to afford the corresponding air- and moisture-stable complexes 3a–e in 66–92% yields. Remarkably, ruthenation of ligand 2f with a methyl rather than an aryl substituent led to a mixture of products, which was not followed up any further. Successful formation of the ruthenium complexes 3a–e was indicated macroscopically by a change of color of the reaction mixture from light to dark red and was confirmed by mass spectrometry and elemental analysis. Evidence for the formation of complexes 3a–e was obtained by ^1^H NMR spectroscopy, with loss of the amide proton resonance and strong deshielding of the NH singlet integrating for one proton only. Moreover, diagnostic shifts of the PYA proton resonances indicate metal coordination. While the pyridylidene protons H_α′_ and H_β_ shifted downfield upon ruthenation, the pyridylidene proton H_*α*_ of all complexes 3a–e resonates at a noticeably higher field compared to the same nucleus in ligand precursors 2a–e (Δ*δ* about −0.5 ppm).

### Spectroscopic and structural analysis of complexes 3a–e

The electronically flexible character of the PYA ligand imparts stability to the formally underligated complex, predominantly through π-donation *via* enhanced contribution of the zwitterionic structure A (Fig. 1b), while π-acidic bonding, especially through resonance form C, promotes the coordination of an additional ligand L to attain the more common three-legged piano-stool ruthenium geometry.^16^ The chemical shift difference between pyridylidene protons H_α_ and H_β_ (*cf.*Scheme 1) is a diagnostic probe to evaluate the electronic structure of the ligand, with larger values indicating a higher contribution of the quinoidal form C.^34^ Analysis of the chemical shifts of the PYA H_α_, H_α′_ and H_β_ protons of complexes 3a–e indicates a similar ligand electronic structure that is independent of the aroyl substituent when measured in CD_2_Cl_2_, a low-polarity solvent (Table 1, entries 1–5). The Δ(H_α_–H_β_) is 0.10(3) ppm and very similar in all complexes. The most notable difference pertains to H_α′_, which is considerably more deshielded in complexes 3d and 3e (*δ*_H_ = 9.22 and 8.94, respectively) in comparison with the frequencies observed for complexes 3a–c, *δ*_H_ = 8.56 (±0.01). This deshielding has been tentatively attributed to a more defined orientation of the naphthyl substituents compared to the phenyl analogues and ensuing hydrogen bonding of the carbonyl oxygen with H_α‘_. When the ^1^H NMR analyses were performed in CD_3_OD instead of CD_2_Cl_2_, a significant increase in the chemical shift differences of these pyridylidene protons to Δ(H_α_–H_β_) = 0.46(3) ppm was observed (entries 6–10). These changes are beyond regular solvent effects as judged by the minor displacement of the cymene signals (Table S1). The larger shift difference in CD_3_OD for complexes 3a–e therefore suggests a higher contribution of the quinoidal form C in polar methanol. Notably, the opposite trend is generally observed, *i.e.* a larger contribution of zwitterionic ligand structure A in polar solvents.^25,34,35^ This opposite trend observed here with complexes 3a–e is similar to that of cognate coordinatively unsaturated iridium PYA complexes.^17^ It has been attributed to reversible solvent coordination leading to a higher electron density at the metal center by the additional solvento ligand. The higher electron density imparted by a σ-bound solvento ligand alleviates the requirement for a strongly π-basic PYA structure to stabilize the metal center and thus leads to a higher predominance of the quinoidal ligand structure C (or B). In contrast, with coordinatively saturated complexes, the polarity of the solvent (rather than its coordination ability) generally leads to a preponderance of the zwitterionic structure.

**Table 1:** Selected ^1^H NMR shifts (ppm) of the CH_α_, CH_α′_, CH_β_ units for complexes 3 and 4

Entry	Complex	Solvent	H_α‘_	H_α_	H_β_	*Δ*(H_α′_ − H_β_)	*Δ*(H_α_ − H_β_)
1	3a	CD_2_Cl_2_	8.55	7.35	7.28	1.27	0.07
2	3b	CD_2_Cl_2_	8.55	7.34	7.25	1.30	0.09
3	3c	CD_2_Cl_2_	8.57	7.38	7.30	1.27	0.08
4	3d	CD_2_Cl_2_	9.22	7.42	7.29	1.93	0.13
5	3e	CD_2_Cl_2_	8.94	7.38	7.28	1.66	0.10
6	3a	CD_3_OD	8.67	7.61	7.18	1.49	0.43
7	3b	CD_3_OD	8.70	7.58	7.15	1.55	0.43
8	3c	CD_3_OD	8.76	7.58	7.09	1.67	0.49
9	3d	CD_3_OD	9.28	7.98	7.12	2.16	0.86
10	3e	CD_3_OD	9.03	7.66	7.20	1.83	0.46
11	4a	CD_3_OD	8.91	7.28	6.63	2.28	0.65
12	4b	CD_3_OD	8.96	7.12	6.38	2.58	0.74
13	4c	CD_3_OD	9.07	7.14	6.40	2.67	0.74
14	4d	CD_3_OD	9.26	7.17	6.42	2.84	0.75

**Scheme 2:**
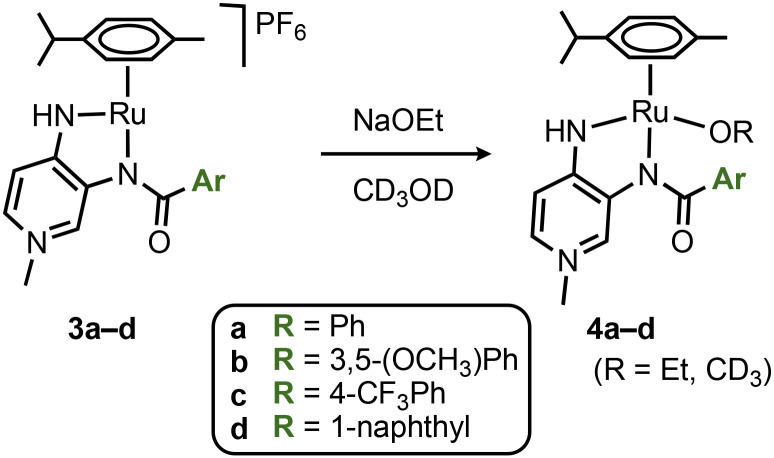
Synthesis of the alkoxide ruthenium complexes 4a–d.

**Fig. 2:**
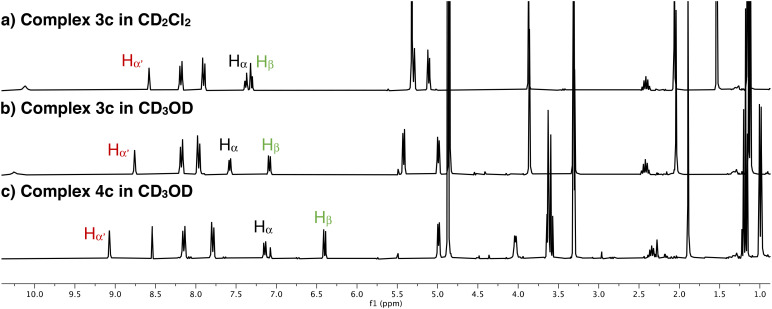
Stacked ^1^H NMR spectra (298 K, 300 MHz) of (a) complex 3c in CD_2_Cl_2_, (b) 3c in CD_3_OD, and (c) 4c in CD_3_OD, showing the chemical shift of pyridylidene protons H_α_, H_α′_, and H_β_.

Preliminary mechanistic studies of the catalytic transfer hydrogenation with complex 3a using EtOH as a benign hydrogen source^36–40^ suggested the formation of the alkoxide complexes 4 as an initially formed species (Scheme 2).^18^ Alkoxide coordination to complexes 3a–d was accomplished with NaOEt in CD_3_OD on the NMR scale. Coordination of an anionic ligand induced an even larger separation of H_α_ and H_β_ protons *Δ*(H_α_–H_β_) = 0.70(5) ppm (Table 1, entries 11–14; Fig. 2), further showcasing the higher contribution of the quinoidal resonance form upon coordination of an anionic ligand as a consequence of the higher electron density at the ruthenium center.^16^ These NMR spectroscopic data therefore underpin the electronically dynamic character of the PYA system (*cf.*Fig. 1b), with a higher contribution of the quinoidal resonance form C in coordinating solvents, which is particularly useful to generate and stabilize the catalytically relevant intermediate 4.

**Fig. 3:**
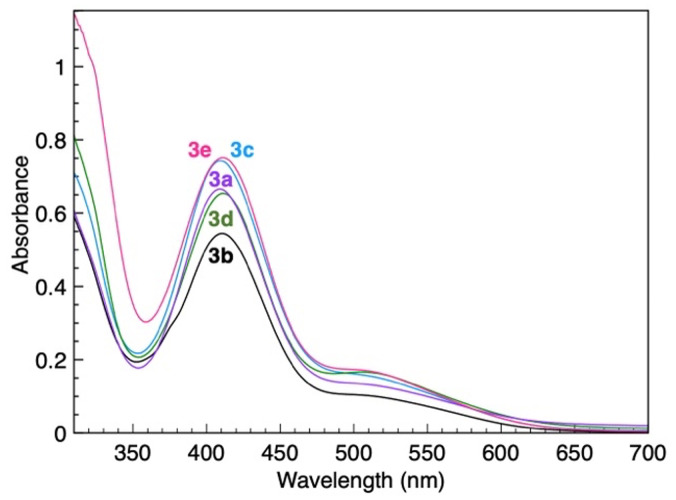
UV-Vis absorption spectra of Ru(ii) PYA complexes 3a–e with various acyl substituents (in CH_2_Cl_2_).

While UV-Vis spectroscopy has been used to probe the donor strength of the ligand system,^25^ no significant trends were observed for complexes 3a–e. All complexes show a diagnostic metal-to-ligand charge transfer (MLCT) band in the visible region with an absorption maximum *λ*_max_ = 410 ± 1 nm (Fig. 3 and Table S2). The similarity of the absorption maxima is in agreement with the essentially identical chemical shifts of the PYA protons for complexes 3a–e, suggesting no impact of the acyl modification on the PYA donor properties. Rather remarkably, the absorption maximum is identical in CH_2_Cl_2_ and CH_3_OH (Fig. S1), indicating no major solvent dependence in contrast to the NMR spectroscopic data. The solvent independence of the absorption band may point to the non-PYA orbitals involved in the lowest energy transition.^41,42^ Electrochemical analysis of complex 3a by cyclic voltammetry revealed several processes, some (quasi-)reversible *e.g.* at *E*_1/2_ = +0.24 and +0.72 V, and others irreversible, *e.g.* at *E*_pa_ = +1.18 and +1.55 V, as a consequence of a combination of ligand- and metal-centered processes (Fig. S2). The difficulty in attributing and thus rationalizing the different processes prevented us from using this technique to probe the donor strength of the ligand.

**Fig. 4:**
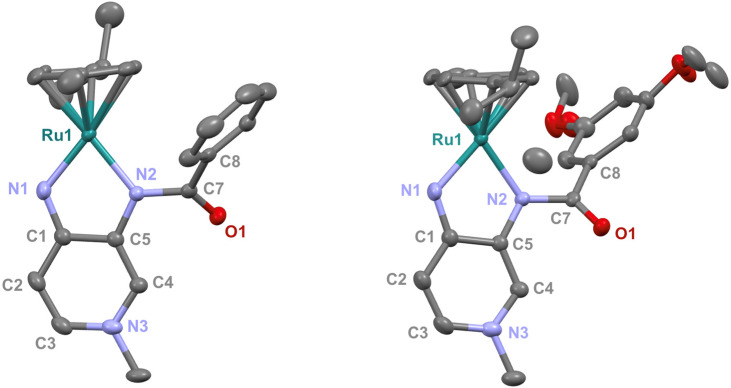
Molecular structure of complexes 3a (left, from ref. 16) and 3b (right) from X-ray diffraction (50% probability ellipsoids; hydrogen atoms and non-coordinating PF_6_^−^ anions have been omitted for clarity).

Suitable crystals of complex 3b for X-ray diffraction analysis were obtained from a CH_2_Cl_2_/Et_2_O mixture. The structure of complex 3b revealed a two-legged piano-stool geometry in the solid state (Fig. 4). The pyramidalization angle *α*, defined as the angle between the centroid of the N–Ru–N moiety, the Ru center, and the centroid of the capping *p*-cymene, is a good indicator of the coordinative unsaturation of these complexes.^8^ Complexes 3a and 3b feature a pyramidalization angle *α* of 176°, confirming the absence of agostic interactions between the ligand and the metal center and also confirming a monomeric structure in the solid state (Table 2). Comparison of the bond lengths with complex 3a, which bears a phenyl group instead of 3,5-dimethoxyphenyl, revealed a highly similar structure within the PYA system. Notably, the C1–N1 bond is in both complexes some 0.06 Å shorter than the C5–N2 bond, pointing to a larger π-bond contribution to the C–N bond *para* to the N–Me site, which reinforces the relevance of quinoidal resonance structures **B** and **C**. While single crystals of complexes 3c and 3e were also grown, they did not diffract enough for a full measurement. Nonetheless, preliminary structure determination of complex 3c showed the same two-legged piano stool structure as identified for 3b.

**Table 2:** Selected bond lengths (Å) and angles (deg) in complexes 3a and 3b

	Complex 3a a	Complex 3b
Ru1–N1	1.963(2)	1.968(2)
Ru1–N2	2.043(2)	2.044(2)
N1–C1	1.340(4)	1.338(3)
N2–C5	1.396(3)	1.396(3)
N2–C7	1.398(3)	1.399(2)
C7–O1	1.216(3)	1.216(2)
C7–C8	1.493(3)	1.500(3)
C_α_–C_β_ b	1.371(8)	1.367(7)
C_β_–C_γ_ b	1.415(6)	1.415(5)
N1–Ru–N2	78.63(8)	78.52
*α* c	176.55	176.75

a From ref. 16.

b C_α_–C_β_ is the average bond length of C2–C3 and C4–C5, C_β_–C_γ_ is the average bond length of C1–C2 and C1–C5.

c Pyramidalization angle *α* is the angle between the centroid of the N–Ru–N moiety, Ru, and the centroid of *p*-cym.

### Chemoselective transfer hydrogenation of α,β-unsaturated ketones

**Fig. 5:**
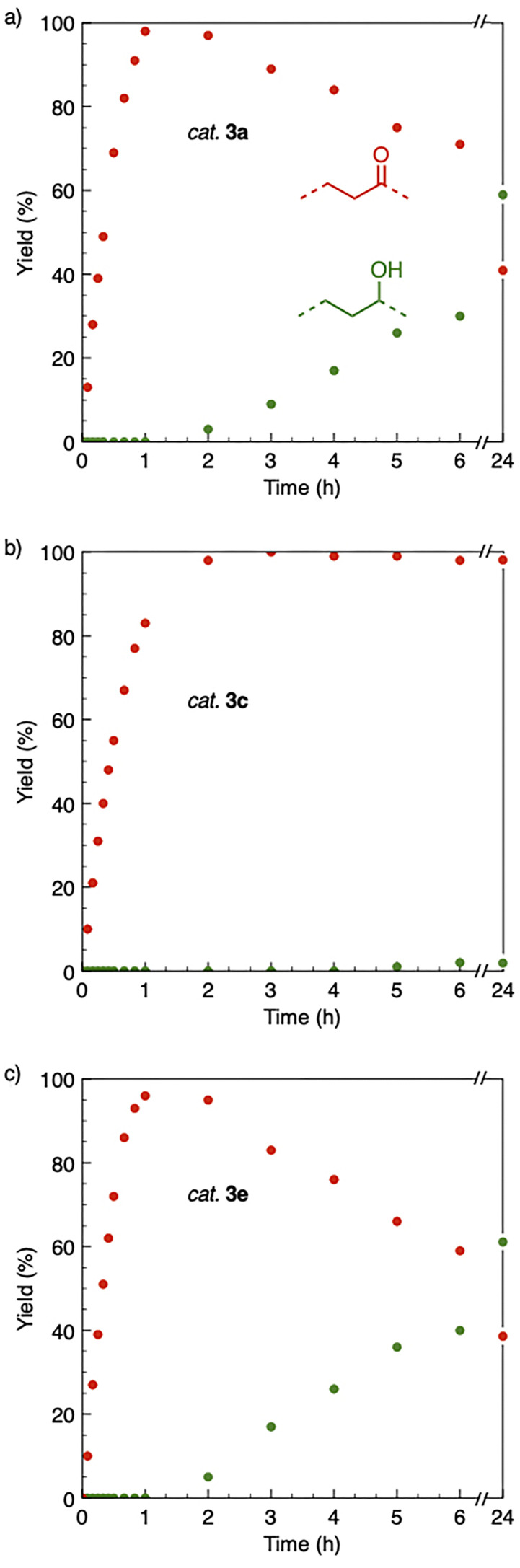
Time-conversion profiles of the Ru-catalyzed transfer hydrogenation of *trans*-chalcone 5a to form subsequently 1,3-diphenylpropan-1-one (red dots) and 1,3-diphenylpropan-1-ol (green dots) using EtOH as a hydrogen source and complexes 3a (a), 3c (b) and 3e (c) as catalyst precursors.

Based on the activity of 3a in the chemoselective transfer hydrogenation of unsaturated ketones,^18^ complexes 3a–e were investigated in this reaction using *trans*-chalcone 5a as a model substrate (Table 3). Under optimized conditions, *i.e.* 1 mol% complex and 5 mol% K_2_CO_3_ in 5 mL EtOH at 25 °C under a N_2_ atmosphere, complex 3a achieved 98% yield of the desired saturated ketone 6a within 1 h. Time-dependent monitoring of the conversion revealed a maximum turnover frequency TOF_C=C_ = 170 h^−1^ (entry 1). The reaction rates were slower when using complexes 3b or 3c, which required 3 and 2 h, respectively, to reach similar yields (entries 2 and 3). The most active complex of this series was the naphthalene-based complex 3d, with a TOF_C=C_ = 200 h^−1^ and achieving 97% yield of ketone 6a in 50 min (entry 4). Similarly, 96% yield of 6a was obtained with complex 3e after a slightly longer reaction time of 1 h (entry 5). Further monitoring of the reaction revealed that upon full consumption of the substrate, hydrogenation of the carbonyl bond was initiated to eventually afford the fully saturated alcohol 7a. This second hydrogenation was slower for all catalysts tested here, as seen from the TOF_C=O_ ≤ 11 h^−1^ (see the SI for details). For example, complex 3a accomplished 59% ketone hydrogenation in 24 h to give alcohol 7a (Fig. 5a). Complex 3b, which is less active in olefin hydrogenation, is also less active in carbonyl hydrogenation, affording a modest 11% yield of the fully saturated product 7a after 24 h (Fig. S3a). Complex 3c is even slower in this second hydrogenation, and the ketone was recovered in 98% after 24 h (Fig. 5c). In sharp contrast, the fastest olefin hydrogenation using complex 3d (*k*_C=C_ = 101 × 10^–5^ h^−1^) also showed high activity towards C=O bond hydrogenation and reached 85% yield of alcohol 7a in 24 h (*k*_C=O_ = 5.5 × 10^−5^ h^−1^, Fig. S3b). Similarly, a lack of selectivity was observed with complex 3e, with 61% of the fully hydrogenated product 7a present after 24 h (Fig. 5e). While these data indicate good general selectivity towards olefin hydrogenation of all complexes 3a–e, the specificity of complex 3c stands out as it provides exclusively the ketone product with only traces of the saturated alcohol even after extended reaction times. Quantitatively, the selectivity is reflected by the ratio of the two reaction rates, Sel = *k*_C=C_/*k*_C=O_. This ratio is 110 for 3c and almost an order of magnitude higher than for the other complexes (20 ± 5). Speculatively, the higher selectivity of 3c may be attributed to a fine balance of the π-stacking behavior of substrate 5a*vs.* the partially saturated 6a with the electron-deficient CF_3_-aryl unit of the catalyst (*vide infra*). In addition, the putative hydride derived from 3c is expected to have the highest hydricity due to the electron-withdrawing character of the acyl group and the ensuing increase of the relevance of the dianionic ligand resonance structure A.

**Table 3:** Comparison of catalytic data for the transfer hydrogenation of *trans*-chalcone 5a using complexes 3a–e a

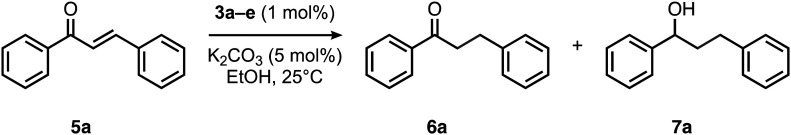								
Entry	Complex	Yield_max_6a (time)	6a : 7a (%) (24 h)	*k*_C=C_ (10^−5^ h^−1^)	*k*_C=O_ (10^−5^ h^−1^)	TOF_C=C_ (h^−1^)	TOF_C=O_ (h^−1^)	Sel b
1	3a	98% (1 h)	41 : 59	74	3.9	170	7	19
2	3b	98% (3 h)	90 : 11	38	∼1.5^c^	100	2	25
3	3c	98% (2 h)	98 : 2	56	0.5	130	1	110
4	3d	97% (0.83 h)	15 : 85	101	5.5	200	11	18
5	3e	96% (1 h)	39 : 61	76	5.1	160	9	15

a Reaction conditions: *trans*-chalcone 5a (0.5 mmol), K_2_CO_3_ (5 mol%), complexes 3a–e (1 mol%) in EtOH (5 mL), 25 °C, N_2_; yields determined by ^1^H NMR spectroscopy relative to 1,3,5-trimethoxybenzene from duplicate runs. *R*^2^ for rates >0.98.

b Sel = selectivity = *k*_C=C_/*k*_C=O_.

c Not sufficient data points for an accurate determination.

**Fig. 6:**
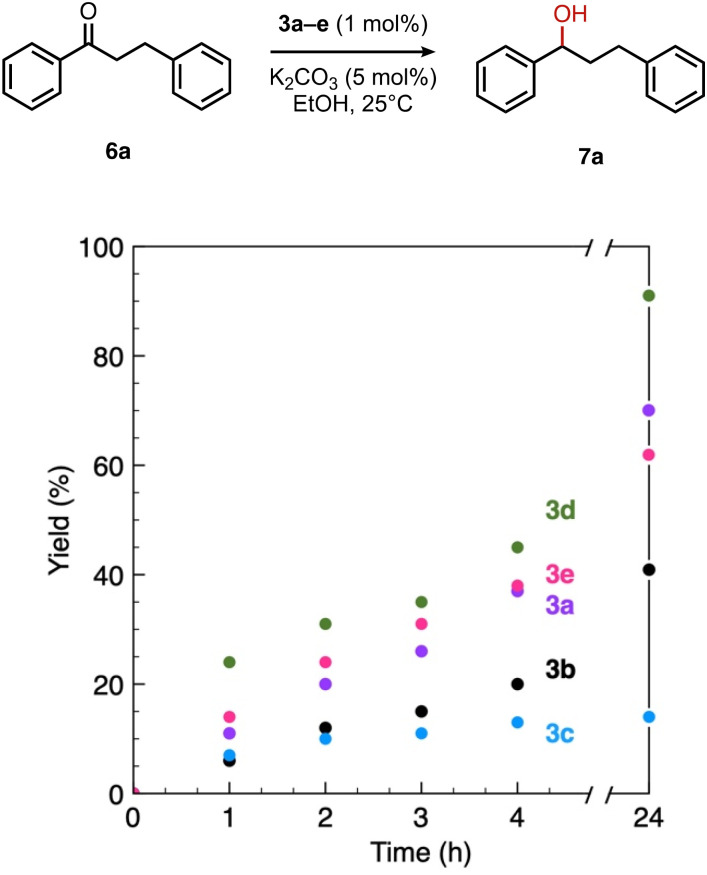
Time-conversion profiles for the transfer hydrogenation of 1,3-diphenylpropan-1-one 6a using EtOH as a hydrogen source under standard conditions catalyzed by complexes 3a–e.

This trend in the ketone hydrogenation activity of complexes 3a–e was further confirmed when using pure 1,3-diphenylpropan-1-one 6a directly as a substrate under optimized catalytic conditions (Fig. 6). In this reaction, complex 3d again provided the most active catalyst, with 91% of alcohol 7a after 24 h *vs.* 85% starting from *trans*-chalcone (Table 4, entry 4). Moderate yields of 70% and 62% were obtained for complexes 3a and 3e, respectively (entries 1 and 5), and 3b achieved only 41% yield within 24 h. This complex was also slow in olefin hydrogenation, indicating that the general catalytic activity of this complex is only modest. Complex 3c gave the lowest activity towards C=O bond hydrogenation, with 14% yield of the alcohol product 6a after 24 h,^43^ thus supporting the poor interaction of the complex with the saturated ketone once olefin hydrogenation took place (see above).

**Table 4:** Comparison of catalytic data for the transfer hydrogenation of 1,3-diphenylpropan-1-one 5a using complexes 3a–e a

Entry	Complex	TON
1	3a	70
2	3b	41
3	3c	14
4	3d	91
5	3e	62

a Reaction conditions: 5a (0.5 mmol), K_2_CO_3_ (5 mol%), complexes 3a–e (1 mol%) in EtOH (5 mL), 25 °C, N_2_; yields determined by ^1^H NMR spectroscopy relative to 1,3,5-trimethoxybenzene from duplicate runs.

The activity of complex 3c in the transfer hydrogenation of C=C bonds is remarkable. The observed rates are orders of magnitude lower than the best Ru-based catalysts for the transfer hydrogenation of C=O bonds,^45–47^ yet considerably higher than that of unactivated C=C bonds.^48,49^ As a consequence, catalysts generally afford allylic alcohols or fully saturated alcohols when exposed to α,β-unsaturated ketone substrates.^31,50–54^ From a synthetic point, therefore, the pronounced selectivity towards C=C bonds over C=O bonds in enone substrates is most remarkable and offers interesting synthetic opportunities.

High selectivity for the olefin transfer hydrogenation of α,β-unsaturated ketones was observed when using complex 3c with the bulkier substrate 5b (Table 5, entries 1 and 2). Within 4 h, complete and selective hydrogenation of the olefinic C=C bond was observed with full retention of the C=O bond. After 24 h, the selectivity was 93% and thus still very high. Also, aliphatic enones such as cyclohexanone were converted well (entry 3), which may point to electronic rather than π-stacking effects that control the selectivity of complex 3c (entry 3). Of note, the selectivity and activity of the catalyst are substrate-dependent. For example, substrate 5d was converted well with both complexes 3a and 3c, though the selectivity was considerably better for complex 3a (entry 4). Likewise, when using the conjugated diene 5e as a substrate, conversion was reduced and reached only 48% after 4 h, with a maximum yield of 40% of compound 6e together with a mixture of isomerized products (entry 5). Longer reaction times did not improve the yield but instead only led to an increase of isomerized products. Similar isomerization side reactions were observed with complex 3a; however, conversion was almost complete, and 73% yield of product 6c was obtained already after 3 h (entry 3). Even though complex 3c is highly selective towards the hydrogenation of the olefin bond in α,β-unsaturated ketones with good retention of selectivity over time (entries 1 and 2), complex 3a is preferred for the conjugated dienone substrate 5c. These data suggest that acyl group modification offers a useful strategy for optimizing the catalyst towards high conversion of a specific substrate.

**Table 5:** Application of complex 3c in the transfer hydrogenation of unsaturated ketones 5a–e a

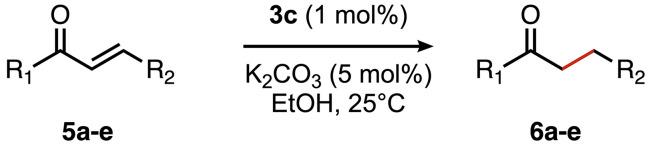					
Entry	Substrate	Product	Time	Conversion	Yield
1	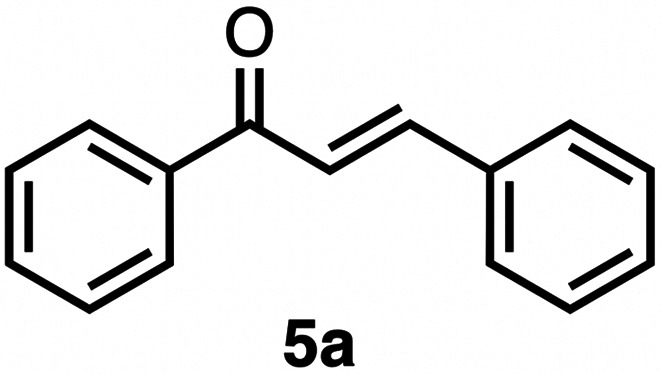	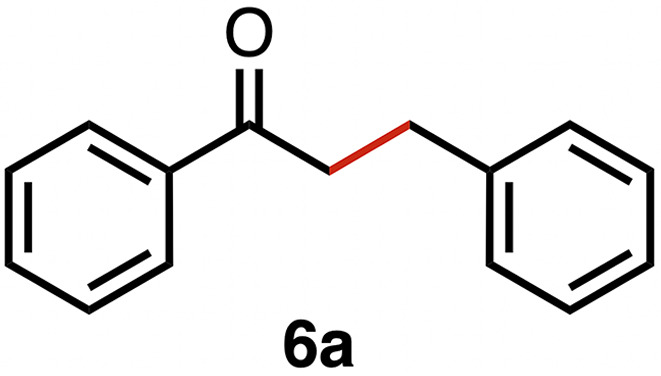	2 h	98%	98%
			24 h	>99%	98%
2	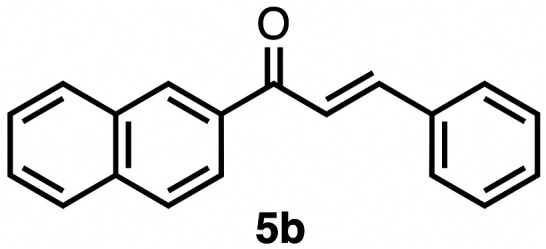	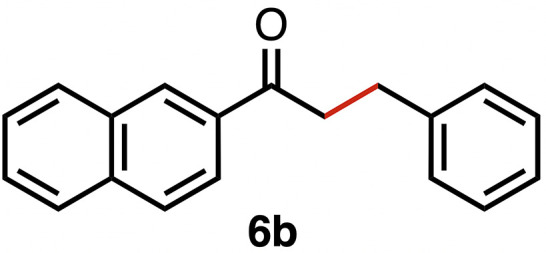	4 h	>99%	99%
			24 h	>99%	93%
3	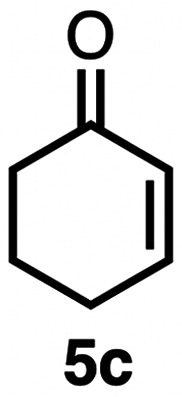	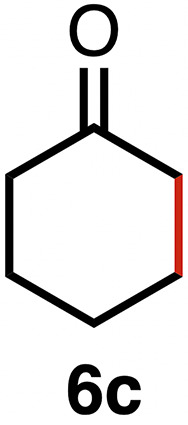	2 h	92%	92%
4	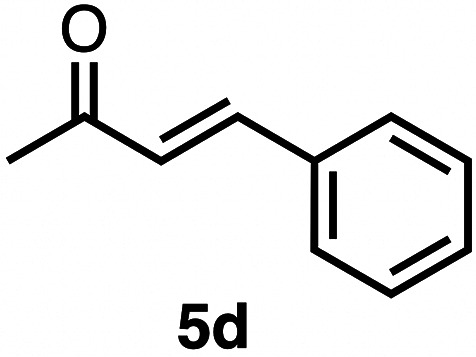	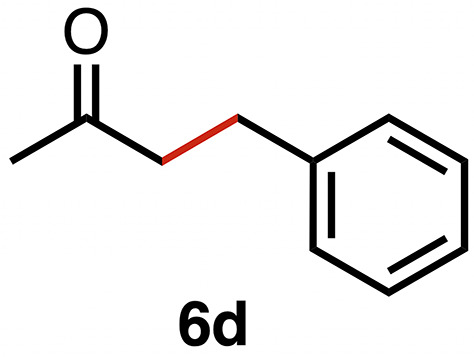	6 h	83%	61%
			6 h^b^	84%	84%
5	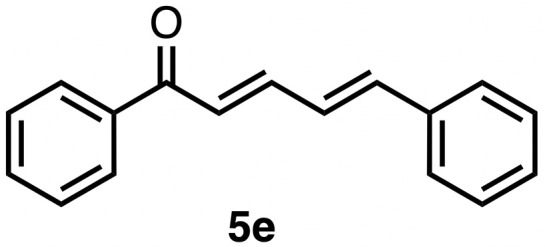	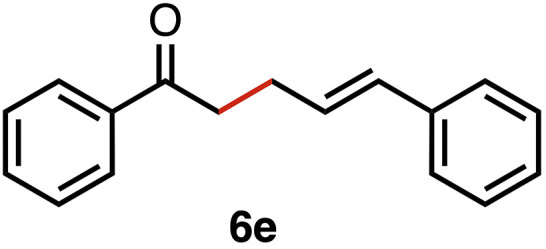	4 h	52%	40%
			24 h	59%	39%
			3 h^b^	94%	73%

a Reaction conditions: substrate (0.5 mmol), K_2_CO_3_ (5 mol%), complex 3c (1 mol%) in EtOH (5 mL), 25 °C, N_2_; yields determined by ^1^H NMR spectroscopy relative to 1,3,5-trimethoxybenzene and the average of duplicate runs.

b Using complex 3a instead of 3c.

## Conclusions

In this work, we presented a series of *N*,*N*′-bidentate PYA ruthenium complexes as efficient catalysts for the selective olefin transfer hydrogenation of α,β-unsaturated ketones using ethanol as a hydrogen source. In particular, the introduction of a *para*-CF_3_-substituted aryl group in the ligand acyl unit drastically improved the selectivity toward olefin hydrogenation, providing almost exclusively the desired ketone product with only minimal traces of the saturated alcohol even after extended reaction periods. The general adaptability of the bidentate ligand, with dynamic metal stabilization through π-acidic quinoidal and π-basic anionic forms, constitutes an attractive platform for further catalytic applications, especially when the electronic properties of the metal-bound substrates and intermediates change throughout a catalytic cycle.

## Experimental

### General

All reactions were performed under air unless stated otherwise. Experiments under an inert atmosphere were carried out using standard Schlenk techniques under an N_2_ atmosphere and in dry deoxygenated solvents. The synthesis of ligands 2a–2e is described in the SI. Complex 3a was prepared according to literature procedures.^18^ Dry solvents were taken from a solvent purification system (SPS), stored over molecular sieves for at least 2 days, and degassed by N_2_ gas bubbling for 30 min. All other compounds were commercially available and used as received. Nuclear magnetic resonance spectra were recorded on a Bruker Avance Neo spectrometer operating at 300 or 400 MHz for ^1^H at room temperature unless otherwise noticed. All chemical shifts (d) are quoted in ppm and coupling constants in Hz. Chemical environments have been assigned through COSY, HSQC/HMBC or NOE NMR spectroscopic experiments. Residual protio solvent resonances were used as an internal reference for ^1^H and ^13^C{^1^H} NMR spectra and externally referenced to SiMe_4_. ^31^P{^1^H} NMR spectra were externally referenced to 85% H_3_PO_4_ (D_2_O). ^19^F{^1^H} NMR chemical shifts were externally referenced to CFCl_3_. Elemental analyses were performed at the DCBP Microanalytic Laboratory using a Thermo Scientific Flash 2000 CHNS-O elemental analyzer. High-resolution mass spectrometry was carried out with a Thermo Scientific LTQ Orbitrap XL (ESI-TOF) by the DCBP mass spectrometry group at the University of Bern. UV-vis spectra were collected on a Shimadzu UV 1800 spectrophotometer, with a silicon photodiode detector ranging from 190 to 1100 nm. Starna Scientific quartz cuvettes (type 23-N/Q/10) with a path length of 10 mm were used. The spectra were collected at 298 K.

#### Synthesis of complex 3b

Compound 2b (107 mg, 0.25 mmol), NaOAc (101 mg, 1.23 mmol) and [RuCl_2_(cym)]_2_ (75 mg, 0.13 mmol) were dissolved in CH_2_Cl_2_ (10 mL). The reaction mixture was refluxed for 16 h, cooled, and filtered through Celite. The filtered solution was washed with saturated NaHCO_3_ solution (3 × 10 mL). The organic phase was dried over Na_2_SO_4_, filtered, and evaporated to dryness. The crude product was purified by gradient column chromatography (neutral Al_2_O_3_; CH_2_Cl_2_ to CH_2_Cl_2_/MeOH 99 : 1) to give analytically pure 3b as a red solid (128 mg, 78%). ^1^H NMR (CD_2_Cl_2_, 298 K, 300 MHz): *δ* 9.90 (bs, 1H, NH), 8.55 (s, 1H, CH_PYA_), 7.34 (dd, *J* = 6.9, 1.7 Hz, 1H, CH_PYA_), 7.25 (d, *J* = 6.8 Hz, 1H, CH_PYA_), 7.17 (d, *J* = 2.4 Hz, 2H, CH_Ar_), 6.78 (t, *J* = 2.3 Hz, 1H, CH_Ar_), 5.36 (d, *J* = 6.2 Hz, 2H, CH_cym_), 5.14 (d, *J* = 6.2 Hz, 2H, CH_cym_), 3.90 (s, 6H, 2 × OCH_3_), 3.86 (s, 3H, NCH_3_), 2.43 (septet, *J* = 6.9 Hz, 1H, CHMe_2_), 2.06 (s, 3H, cym-CH_3_), 1.15 (d, *J* = 6.9 Hz, 6H, CH(C*H*_3_)_2_). ^13^C{^1^H} NMR (CD_2_Cl_2_, 298 K, 75 MHz): *δ* 182.12 (C=O), 161.24 (C_Ar_), 160.99 (C_PYA_), 142.34 (C_Ar_), 140.94 (C_PYA_), 133.29 (CH_PYA_), 129.65 (CH_PYA_), 110.34 (CH_PYA_), 108.24 (CH_Ar_), 104.29 (CH_Ar_), 100.90 (C_cym_), 92.10 (C_cym_), 82.09 (CH_cym_), 79.41 (CH_cym_), 56.24 (OCH_3_), 46.42 (NCH_3_), 32.09 (CHMe_2_), 23.36 (CH(*C*H_3_)_2_), 19.96 (cym-CH_3_). ^19^F{^1^H} NMR (CD_2_Cl_2_, 298 K, 282 MHz): *δ* −72.44 (d, J = 711 Hz, PF_6_). ^31^P{^1^H} NMR (CD_3_CN, 298 K, 121 MHz): −144.17 (septet, *J* = 711 Hz, PF_6_). HR-MS ESI: 522.1313 (522.1325 calcd for [M − PF_6_]^+^). Elem. anal. found (calcd) for C_25_H_30_F_6_N_3_O_3_PRu: C 45.82 (45.05), H 4.52 (4.54), N 6.19 (6.30)%.

#### Synthesis of complex 3c

Compound 2c (67 mg, 0.15 mmol), NaOAc (61 mg, 0.75 mmol), [RuCl_2_(cym)]_2_ (46 mg, 0.07 mmol) and NaPF_6_ (63 mg, 0.37 mmol) were dissolved in CH_2_Cl_2_ (10 mL). The reaction mixture was refluxed for 16 h, cooled, and filtered through Celite. The filtrate was washed with saturated NaHCO_3_ solution (3 × 5 mL). The organic phase was dried over Na_2_SO_4_, filtered, and evaporated to dryness. The crude product was purified by gradient column chromatography (neutral Al_2_O_3_; CH_2_Cl_2_ to CH_2_Cl_2_/MeOH 99 : 1) to give complex 3c as a red solid (71 mg, 70%). Recrystallization from CH_2_Cl_2_ and Et_2_O afforded an analytically pure material. ^1^H NMR (CD_2_Cl_2_, 298 K, 300 MHz): *δ* 10.13 (bs, 1H, NH), 8.57 (s, 1H, CH_PYA_), 8.18 (d, *J* = 8.0 Hz, 2H, CH_Ar_), 7.90 (d, *J* = 8.1 Hz, 2H, CH_Ar_), 7.38 (dd, *J* = 6.9, 1.7 Hz, 1H, CH_PYA_), 7.30 (d, *J* = 6.9 Hz, 1H, CH_PYA_), 5.30 (d, *J* = 6.2 Hz, 2H, CH_cym_), 5.11 (d, *J* = 6.2 Hz, 2H, CH_cym_), 3.87 (s, 3H, NCH_3_), 2.42 (septet, *J* = 6.9 Hz, 1H, CHMe_2_), 2.06 (s, 3H, cym-CH_3_), 1.15 (d, *J* = 6.9 Hz, 6H, CH(C*H*_3_)_2_). ^13^C{^1^H} NMR (CD_2_Cl_2_, 298 K, 75 MHz): *δ* 181.11 (C=O), 161.13 (C_PYA_), 143.90 (C_Ar_), 140.70 (C_PYA_), 133.74 (CH_PYA_), 130.35 (CH_Ar_), 130.10 (CH_PYA_), 125.94 (q, *J* = 4.0 Hz, CH_Ar_), 110.60 (CH_PYA_), 101.13 (C_cym_), 91.84 (C_cym_), 81.99 (CH_cym_), 79.33 (CH_cym_), 46.51 (NCH_3_), 32.12 (CHMe_2_), 23.33 CH(*C*H_3_)_2_, 20.00 (cym-CH_3_). ^19^F{^1^H} NMR (CD_2_Cl_2_, 298 K, 282 MHz): *δ* −63.14 (s, CF_3_), −72.13 (d, *J* = 711 Hz, PF_6_). ^31^P{^1^H} NMR (CD_3_CN, 298 K, 121 MHz): *δ* −144.11 (septet, *J* = 711 Hz, PF_6_). HR-MS ESI: 530.0976 (530.0988 calcd for [M − PF_6_]^+^). Elem. anal. found (calcd) for C_24_H_25_F_9_N_3_OPRu × 0.5 Et_2_O: C 44.06 (43.89), H 3.91 (4.25), N 5.97 (5.01)%.

#### Synthesis of complex 3d

Complex 3d was prepared using a procedure analogous to that described for 3c starting from compound 2d (144 mg, 0.33 mmol), NaOAc (138 mg, 1.68 mmol), [RuCl_2_(cym)]_2_ (103 mg, 0.17 mmol) and NaPF_6_ (141 mg, 0.84 mmol). Complex 3d was obtained as a red solid (152 mg, 68%). ^1^H NMR (CD_2_Cl_2_, 298 K, 300 MHz): *δ* 10.11 (bs, 1H, NH), 9.22 (s, 1H, CH_PYA_), 8.20 (d, *J* = 8.2 Hz, 1H, CH_Ar_), 8.16 (d, *J* = 8.2 Hz, 1H, CH_Ar_), 8.07 (d, *J* = 7.7 Hz, 1H, CH_Ar_), 7.97 (d, *J* = 7.1, 1H, CH_Ar_), 7.74–7.58 (m, 3H, CH_Ar_), 7.42 (dd, *J* = 6.8, 1.8 Hz, 1H, CH_PYA_), 7.29 (d, *J* = 6.8 Hz, 1H, CH_PYA_), 5.07 (br s, 1H, CH_cym_), 4.89 (br s, 1H, CH_cym_),4.60 (br s, 2H, CH_cym_), 3.92 (s, 3H, NCH_3_), 2.11 (septet, *J* = 6.9 Hz, 1H, CHMe_2_), 1.71 (s, 3H, cym-CH_3_), 1.04 (d, *J* = 6.9 Hz, 6H, CH(C*H*_3_)_2_). ^13^C{^1^H} NMR (CD_2_Cl_2_, 298 K, 75 MHz): *δ* 182.85 (C=O), 161.44 (C_PYA_), 140.03 (C_Ar_), 139.82 (C_PYA_), 134.08 (CH_PYA_), 133.97 (C_Ar_), 131.69 (CH_PYA_), 131.57 (CH_Ar_), 131.50 (C_Ar_), 129.24 (CH_Ar_), 127.99 (CH_Ar_), 127.50 (CH_Ar_), 127.33 (CH_Ar_), 126.14 (CH_Ar_), 125.31 (CH_Ar_), 110.38 (CH_PYA_), 100.19 (C_cym_), 91.30 (C_cym_), 82.03 (CH_cym_), 79.24 (CH_cym_), 46.58 (NCH_3_), 31.83 (CHMe_2_), 23.32 (CH(*C*H_3_)_2_), 19.71 (cym-CH_3_). ^19^F{^1^H} NMR (CD_2_Cl_2_, 298 K, 282 MHz): *δ* −72.17 (d, *J* = 712 Hz, PF_6_). ^31^P{^1^H} NMR (CD_3_CN, 298 K, 121 MHz): *δ* −144.10 (septet, *J* = 712 Hz, PF_6_). HR-MS ESI: 512.1260 (512.1270 calcd for [M − PF_6_]^+^). Elem. anal. found (calcd) for C_27_H_28_F_6_N_3_OPRu: C 49.34 (49.39), H 4.19 (4.30), N 6.21 (6.40)%.

#### Synthesis of complex 3e

Complex 3e was prepared using a procedure analogous to that described for 3c starting from compound 2e (47 mg, 0.10 mmol), NaOAc (43 mg, 0.51 mmol), [RuCl_2_(cym)]_2_ (32 mg, 0.05 mmol) and NaPF_6_ (44 mg, 0.25 mmol). Complex 3e was isolated as a red solid (66 mg, 92%). ^1^H NMR (CD_2_Cl_2_, 298 K, 300 MHz): *δ* 10.02 (bs, 1H, NH), 8.94 (s, 1H, CH_PYA_), 8.49–8.42 (m, 1H, CH_Ar_), 8.39–8.31 (m, 1H, CH_Ar_), 7.97 (d, *J* = 8.0 Hz, 1H, CH_Ar_), 7.68–7.60 (m, 2H, CH_Ar_), 7.38 (dd, *J* = 6.9, 1.7 Hz, 1H, CH_PYA_), 7.28 (d, *J* = 6.9 Hz, 1H, CH_PYA_), 7.00 (d, *J* = 8.0 Hz, 1H, CH_Ar_), 5.15 (br s, 1H, CH_cym_), 5.01 (br s, 1H, CH_cym_), 4.77 (br s, 2H, CH_cym_), 4.16 (s, 3H, OCH_3_), 3.90 (s, 3H, NCH_3_), 2.23 (septet, *J* = 6.7 Hz, 1H, CHMe_2_), 1.81 (s, 3H, cym-CH_3_), 1.07 (d, *J* = 6.9 Hz, 6H, CH(C*H*_3_)_2_). ^13^C{^1^H} NMR (CD_2_Cl_2_, 298 K, 75 MHz): *δ* 182.91 (C=O), 161.21 (C_PYA_), 158.87 (C_Ar_), 140.44 (C_PYA_), 133.51 (CH_PYA_), 132.93 (C_Ar_), 131.04 (C_Ar_), 130.53 (CH_Ar_), 130.16 (CH_PYA_), 128.59 (CH_Ar_), 126.58 (CH_Ar_), 126.09 (CH_Ar_), 125.98 (C_Ar_), 123.16 (CH_Ar_), 110.34 (CH_PYA_), 102.94 (CH_Ar_), 100.52 (C_cym_), 91.43 (C_cym_), 81.87 (CH_cym_), 79.20 (CH_cym_), 56.47 (OCH_3_), 46.50 (NCH_3_), 31.94 (CHMe_2_), 23.38 CH(*C*H_3_)_2_, 19.86 (cym-CH_3_). ^19^F{^1^H} NMR (CD_2_Cl_2_, 298 K, 282 MHz): *δ* −72.37 (d, *J* = 711 Hz, PF_6_). ^31^P{^1^H} NMR (CD_3_CN, 298 K, 121 MHz): *δ* −144.14 (septet, *J* = 709 Hz, PF_6_). HR-MS ESI: 542.1366 (542.1376 calcd for [M − PF_6_]^+^). Elem. anal. found (calcd) for C_28_H_30_F_6_N_3_O_2_PRu: C 48.87 (48.98), H 4.44 (4.40), N 5.61 (6.12)%.

### General catalytic procedure

In a 10 mL round bottom flask, the substrate (0.5 mmol), complex 1 (1 mol%), and 1,3,5-trimethoxybenzene or mesitylene (10 mol%, internal standard) were dissolved in EtOH (5 mL) and the solution was degassed with N_2_ for 10 min. The catalytic run was started with the injection of K_2_CO_3_ (5 mol%, 2 M solution in H_2_O) and the tube was placed in a thermostated oil bath (25 °C). The reaction was monitored by ^1^H NMR spectroscopy by taking aliquots (*ca.* 0.1 mL) at set times under N_2_, which were dissolved in CDCl_3_ (0.5 mL) to determine the spectroscopic conversion and yields relative to the internal standard.

### Crystal structure determination

A suitable crystal of 3b was mounted and then transferred into a cold stream of nitrogen (173 K). All measurements were made on a RIGAKU Synergy S area-detector diffractometer using mirror optics monochromated Cu *K*α radiation (*λ* = 1.54184 Å). Data reduction was performed using the CrysAlisPro^55^ program. The intensities were corrected for Lorentz and polarization effects, and an absorption correction based on the multi-scan method using SCALE3 ABSPACK in CrysAlisPro^55^ was applied. The structure was solved by intrinsic phasing using SHELXT,^56^ which revealed the positions of all non-hydrogen atoms. All non-hydrogen atoms were refined anisotropically. H-atoms were assigned in geometrically calculated positions and refined using a riding model (1.2*U*_eq_ of the parent atom and 1.5*U*_eq_ of methyl groups) except for the hydrogen atom attached to N1, which was located from the difference density map and had its position and isotropic displacement parameter refined freely. Refinement of the structure was carried out on *F*^2^ using full-matrix least-squares procedures, which minimized the function Σw(*F*_o_^2^ − *F*_c_^2^)^2^. All calculations were performed using the SHELXL-2014/7^57^ program in OLEX2.^58^ Data collection and refinement parameters for 3b are given in Table S13. Crystallographic data for this structure have been deposited at the Cambridge Crystallographic Data Centre (CCDC) as supplementary publication number 2422796.

## Conflicts of interest

The authors declare no conflicts of interest.

## Supplementary Material

DT-054-D5DT01348H-s001

DT-054-D5DT01348H-s002

## Data Availability

The data supporting this article have been included as part of the SI. Supplementary information is available: synthetic procedures, analytical data and NMR spectra, catalytic data, and details on crystal structure determination. See DOI: https://doi.org/10.1039/d5dt01348h CCDC 2422796 contains the supplementary crystallographic data for this paper.^59^
