# Supplementary material for: Ligand modification for the tuning of activity and selectivity in the chemoselective transfer hydrogenation of α,β-unsaturated carbonyls using EtOH as a hydrogen source
Source: Dalton Trans. 2025 Jul 28;54(35):13155–65. doi: 10.1039/d5dt01348h (PMC12356170; doi:10.1039/d5dt01348h)
Supplement: DT-054-D5DT01348H-s001 [file DT-054-D5DT01348H-s001.pdf]

Supplementary Information (SI) for Dalton Transactions.
This journal is © The Royal Society of Chemistry 2025

Electronic Supporting Information

Ligand Modification for the Tuning of Activity and Selectivity in Chemoselective Transfer Hydrogenation of α,β-Unsaturated Carbonyls using EtOH as Hydrogen Source

Alicia Beaufils,[a] Nicole Elia,[a] Sabela Reuge,[a] and Martin Albrecht*,[a]

Department of Chemistry, Biochemistry, and Pharmaceutical Sciences, University of Bern, Freiestrasse 3, CH-3012 Bern (Switzerland)
E-Mail: martin.albrecht@unibe.ch

TABLE OF CONTENTS

S.1 EXPERIMENTAL PROCEDURES FOR LIGAND SYNTHESIS S2
S.1.1 Synthesis of amides **1b–e** S2
S.1.2 Synthesis of pyridinium salts **2b–e** S4
S.2 ANALYTICAL DATA OF COMPLEXES 3 AND 4 .... S6
S.2.1 Synthesis and spectroscopic data of complexes **4a–d** S8
S.3 CATALYTIC DATA S9
S.3.1 General catalytic procedure for catalytic transfer hydrogenation S9
S.3.2 Determination of rate constants S9
S.3.3 Characterization of transfer hydrogenation products **6a-6e** S15
S5 CRYSTAL STRUCTURE DETERMINATION S16
S6 NMR SPECTRA OF ALL COMPOUNDS S18
S7 REFERENCES S40

S.1 Experimental procedures for ligand synthesis

General. All reactions were performed under air unless stated otherwise. Experiments under inert atmosphere were carried out using standard Schlenk techniques under N_2 atmosphere and dry deoxygenated solvents. Dry solvents were taken from a solvent purification system (SPS), stored over molecular sieves for at least 2 days, and degassed by N_2 gas bubbling for 30 min. All compounds were commercially available and used as received. The syntheses of tert-butyl (3-aminopyridin-4-yl)carbamate[S1] and complex **3a** have been reported previously.[S2] Nuclear magnetic resonance spectra were recorded on a Bruker Avance Neo spectrometer operating at 300 or 400 MHz for ^{1}H at room temperature unless otherwise noticed. All chemical shifts (d) are quoted in ppm and coupling constants in Hz. Chemical environments have been assigned through COSY, HSQC/HMBC or NOE NMR spectroscopic experiments. Residual protio solvent resonances were used as an internal reference for ^{1}H and $^{13}C\{^{1}H\}$ NMR spectra, and externally referenced to $SiMe_4$. $^{31}P\{^{1}H\}$ NMR spectra were referenced externally to 85% H_3PO_4 (D_2O). $^{19}F\{^{1}H\}$ NMR chemical shifts are externally referenced to $CFCl_3$. The following abbreviations are used: s, singlet; d, doublet; t, triplet; sept, septet; m, multiplet. Elemental analyses were performed at the DCBP Microanalytic Laboratory using a Thermo Scientific Flash 2000 CHNS-O elemental analyzer. High-resolution mass spectrometry was carried out with a Thermo Scientific LTQ Orbitrap XL (ESI-TOF) by the DCBP mass spectrometry group at the University of Bern. UV-vis spectra were collected on Shimadzu UV 1800 Spectrophotometer, with a silicon photodiode detector ranging from 190 to 1100 nm. Starna Scientific quartz cuvettes (type 23-N/Q/10) with a path length of 10 mm were used. The spectra were collected at 298 K.

S.1.1 Synthesis of amides 1b–e

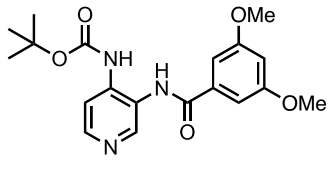

***Tert*-butyl(3-(3,5-dimethoxybenzamido)pyridin-4-yl)carbamate (1b).** *Tert*-butyl(3-aminopyridin-4-yl)carbamate (500 mg, 2.4 mmol, 1 eq) and Et_3N (355 µL, 2.63 mmol, 1.1 eq) were dissolved in THF (15 mL) and 3,5-dimethoxybenzoyl chloride (527 mg, 2.6 mmol, 1.1 eq) was added dropwise under agitation at 0 °C, and then stirred for 16 h at 70 °C. The reaction mixture was cooled to room temperature, and all volatiles were removed under reduced pressure. Water was added and the solid was collected by filtration and solubilized in THF. The solution was dried over Na_2SO_4, filtered, and evaporated to dryness. The crude product was purified by column chromatography (SiO_2; hexane/ethyl acetate 9:1 to 1:1) to afford **1b** as a white solid (622 mg, 70%).

^{1}H NMR (CD_3CN, 298 K, 300 MHz): δ 8.74 (bs, 1H, N**H**), 8.46 (s, 1H, C$\mathbf{H}_{PYA}$), 8.35 (d, J = 5.6 Hz, 1H, C$\mathbf{H}_{PYA}$), 7.89 (d, J = 5.6 Hz, 1H, C$\mathbf{H}_{PYA}$), 7.74 (bs, 1H, N**H**), 7.11 (d, J = 2.3 Hz, 2H, C$\mathbf{H}_{Ar}$), 6.72 (t, J = 2.3 Hz, 1H, C$\mathbf{H}_{Ar}$), 3.85 (s, 6H, C$\mathbf{H}_{3\text{-OMe}}$), 1.48 (s, 9H, C$\mathbf{H}_{3\text{-}t\text{Bu}}$). **$^{13}C\{^{1}H\}$ NMR (CD_3CN, 298 K, 75 MHz):** δ 167.62 ($\mathbf{C}_{C=O}$), 162.06 ($\mathbf{C}_{Ar}$), 153.56 ($\mathbf{C}_{C=O}$), 149.23 ($\mathbf{C}H_{PYA}$), 149.08 ($\mathbf{C}H_{PYA}$), 141.98 ($\mathbf{C}_{PYA}$), 136.80

(**C**$_{Ar}$), 124.34 (**C**$_{PYA}$), 115.53 (**C**H$_{PYA}$), 106.63 (**C**H$_{Ar}$), 105.05 (**C**H$_{Ar}$), 82.08 (**C**$_{tBu}$), 56.40 (**C**H$_{3\text{-}OMe}$), 28.37 (**C**H$_{3\text{-}tBu}$). **ESI-MS (MeCN, calc. for [$C_{19}H_{23}N_3O_5$]$^+$):** 374.1709 (374.1710).

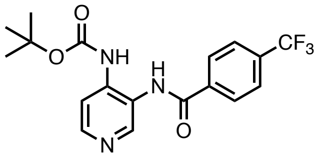

***Tert*-butyl(3-(4-(trifluoromethyl)benzamido)pyridin-4-yl)carbamate (1c)**. Using the procedure described for **1b**, compound **1c** was obtained from *tert*-butyl(3-aminopyridin-4-yl)carbamate (250 mg, 2.4 mmol, 1 eq) and 4-(trifluoromethyl)benzoyl chloride (160 µL, 2.6 mmol, 1.1 eq) as a white solid (302 mg, 66%).

^{1}H NMR (CD$_3$CN, 298 K, 300 MHz): δ 8.91 (bs, 1H, N**H**), 8.49 (s, 1H, C**H**$_{PYA}$), 8.36 (d, J = 5.6 Hz, 1H, C**H**$_{PYA}$), 8.13 (d, J = 8.3 Hz, 2H, C**H**$_{Ar}$), 7.90 (d, J = 5.6 Hz, 1H, C**H**$_{PYA}$), 7.86 (d, J = 8.2 Hz, 2H, C**H**$_{Ar}$), 7.78 (bs, 1H, N**H**), 1.48 (s, 9H, C**H**$_{3\text{-}tBu}$). **^{13}C{^{1}H} NMR (CD$_3$CN, 298 K, 75 MHz):** δ 166.74 (**C**$_{C=O}$), 153.62 (**C**$_{C=O}$), 149.37 (**C**H$_{PYA}$), 149.20 (**C**H$_{PYA}$), 142.20 (**C**$_{PYA}$), 138.46 (**C**$_{Ar}$), 133.59 (**C**$_{Ar}$), 129.52 (**C**H$_{Ar}$), 129.29 (**C**$_{Ar}$), 126.56 (q, J = 3.9 Hz, **C**H$_{Ar}$), 123.23 (**C**$_{PYA}$), 115.45 (**C**H$_{PYA}$), 82.16 (**C**$_{tBu}$), 28.35 (**C**H$_{3\text{-}tBu}$). **ESI-MS (MeCN, calc. for [$C_{18}H_{18}F_3N_3O_3$]$^+$):** 382.1371 (382.1373).

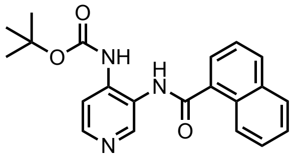

***Tert*-butyl(3-(1-naphthamido)pyridin-4-yl)carbamate (1d).** Using the procedure described for **1b**, compound **1d** was prepared from *tert*-butyl(3-aminopyridin-4-yl)carbamate (500 mg, 2.4 mmol, 1 eq) and 1-naphthoyl chloride (310 µL, 2.6 mmol, 1.1 eq) and was obtained as a white solid (612 mg, 70%).

^{1}H NMR (CD$_3$CN, 298 K, 300 MHz): δ 8.77 (bs, 1H, N**H**), 8.59 (s, 1H, C**H**$_{PYA}$), 8.40–8.37 (m, 2H, C**H**$_{PYA}$, C**H**$_{Ar}$), 8.09 (d, J = 8.3 Hz, 1H, C**H**$_{Ar}$), 8.01–7.99 (m, 1H, C**H**$_{Ar}$), 7.93–7.89 (m, 2H, C**H**$_{PYA}$, C**H**$_{Ar}$), 7.86 (bs, 1H, N**H**), 7.65–7.59 (m, 3H, C**H**$_{Ar}$), 1.48 (s, 9H, C**H**$_{3\text{-}tBu}$). **^{13}C{^{1}H} NMR (CD$_3$CN, 298 K, 75 MHz):** δ 169.94 (**C**$_{C=O}$), 153.56 (**C**$_{C=O}$), 149.06 (**C**H$_{PYA}$), 148.96 (**C**H$_{PYA}$), 141.61 (**C**$_{PYA}$), 134.78 (**C**$_{Ar}$), 134.13 (**C**$_{Ar}$), 132.31 (**C**H$_{Ar}$), 131.15 (**C**$_{Ar}$), 129.46 (**C**H$_{Ar}$), 128.26 (**C**H$_{Ar}$), 127.60 (**C**H$_{Ar}$), 127.12 (**C**H$_{Ar}$), 126.37 (**C**H$_{Ar}$), 125.85 (**C**H$_{Ar}$), 124.58 (**C**$_{PYA}$), 115.83 (**C**H$_{PYA}$), 82.06 (**C**$_{tBu}$), 28.39 (**C**H$_{3\text{-}tBu}$). **ESI-MS (MeCN, calc. for [$C_{21}H_{21}N_3O_3$]$^+$):** 364.1649 (364.1656).

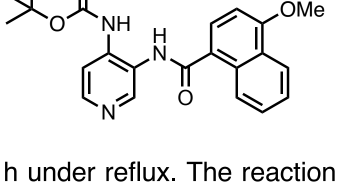

***Tert*-butyl(3-(4-methoxy-1-naphthamido)pyridin-4-yl)carbamate (1e).** Adapted from a reported procedure,[S3] 4-methoxy-1-naphthoic acid (250 mg, 1.24 mmol, 1 eq), $SOCl_2$ (180 µL, 2.48 mmol, 2 eq) and few drops of DMF were dissolved in CH_2Cl_2 (10 mL) and the mixture was stirred for 6 h under reflux. The reaction mixture was cooled to room temperature, and all volatiles were removed under reduced pressure, and the precipitate was washed thoroughly with Et_2O and used without further purification. The obtained 4-methoxy-1-naphthoic chloride was dissolved in THF and added dropwise under agitation at 0 °C to a solution of *tert*-butyl(3-aminopyridin-4-yl)carbamate (258 mg, 1.24 mmol, 1 eq) and Et_3N (183 µL, 1.36 mmol, 1.1 eq) in THF. The reaction mixture was cooled to room temperature,

and all volatiles were removed under reduced pressure. The crude product was purified by column chromatography (SiO_2; hexane/ethyl acetate 8:2 to 1:1) to afford the corresponding product as an off-white solid (262 mg, 54%).

^{1}H NMR (CD_3CN, 298 K, 300 MHz): δ 8.69 (bs, 1H, N**H**), 8.55 (s, 1H, C**H**$_{PYA}$), 8.47 (d, *J* = 7.9 Hz, 1H, C**H**$_{Ar}$), 8.37–8.30 (m, 2H, C**H**$_{PYA}$, C**H**$_{Ar}$), 7.94–7.89 (m, 3H, N**H**, C**H**$_{PYA}$, C**H**$_{Ar}$), 7.66–7.55 (m, 2H, C**H**$_{Ar}$), 7.00 (d, *J* = 8.1 Hz, 1H, C**H**$_{Ar}$), 4.08 (s, 3H, OC**H**$_3$), 1.48 (s, 9H, C**H**$_{3\text{-}tBu}$). **^{13}C{^{1}H} NMR (CD_3CN, 298 K, 75 MHz):** δ 169.93 (**C**$_{C=O}$), 158.80 (**C**$_{Ar}$), 153.53 (**C**$_{C=O}$), 148.82 (2×**C**H$_{PYA}$), 141.71 (**C**$_{PYA}$), 132.60 (**C**$_{Ar}$), 128.89 (**C**H$_{Ar}$), 128.67 (**C**H$_{Ar}$), 126.90 (**C**H$_{Ar}$), 126.55 (**C**$_{Ar}$), 126.41 (**C**H$_{Ar}$), 125.83 (**C**$_{Ar}$), 124.85 (**C**$_{PYA}$), 123.01 (**C**H$_{Ar}$), 115.73 (**C**H$_{PYA}$), 103.79 (**C**H$_{Ar}$), 81.99 (**C**$_{tBu}$), 56.70 (O**C**H$_3$), 28.38 (**C**H$_{3\text{-}tBu}$). **ESI-MS (MeCN, calc. for [$C_{22}H_{23}N_3O_4$]$^+$):** 394.1751 (394.1761).

S.1.2 Synthesis of pyridinium salts 2b–e

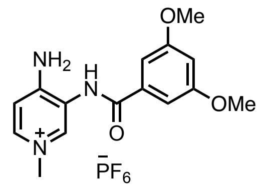

4-amino-3-(3,5-dimethoxybenzamido)-1-methylpyridin-1-ium trifluoromethanesulfonate (2b). Under an N_2 atmosphere, compound **1b** (100 mg, 0.27 mmol, 1 eq) and MeOTf (147 µL, 1.3 mmol, 5 eq) were dissolved in dry CH_2Cl_2 (5 mL) and heated at 42 °C in a closed system for 16 h. After cooling to room temperature, the reaction mixture was concentrated to few mL and MeCN was added until all material was solubilized (ca. 1 mL). Then, a solution of NH_4PF_6 (218 mg, 1.3 mmol, 5 eq) in H_2O (5 mL) was added. After stirring the mixture at rt for 30 min, MeCN was removed under reduced pressure, resulting in the precipitation of a white solid. The mixture was stored at 4 °C for 16 h, then the precipitate was collected by filtration and dried in vacuo to yield the corresponding product as a white solid (65 mg, 43%).

^{1}H NMR (CD_3CN, 298 K, 300 MHz): δ 8.44 (bs, 1H, N**H**), 8.17 (d, *J* = 1.8 Hz, 1H, C**H**$_{PYA}$), 7.87 (dd, *J* = 7.1, 1.8 Hz, 1H, C**H**$_{PYA}$), 7.09 (d, *J* = 2.3 Hz, 2H, C**H**$_{Ar}$), 6.99 (d, *J* = 7.1 Hz, 1H, C**H**$_{PYA}$), 6.74 (t, *J* = 2.3 Hz, 1H, C**H**$_{Ar}$), 6.58 (bs, 2H, N**H$_2$**), 3.92 (s, 3H, NC**H$_3$**), 3.85 (s, 6H, 2×OC**H$_3$**). **^{13}C{^{1}H} NMR (CD_3CN, 298 K, 75 MHz):** δ 167.20 (**C**$_{C=O}$), 162.08 (**C**$_{Ar}$), 156.06 (**C**$_{PYA}$), 143.03 (**C**H$_{PYA}$), 142.94 (**C**H$_{PYA}$), 136.21 (**C**$_{Ar}$), 120.93 (**C**$_{PYA}$), 111.40 (**C**H$_{PYA}$), 106.77 (**C**H$_{Ar}$), 105.11 (**C**H$_{Ar}$), 56.45 (O**C**H$_3$), 46.22 (N**C**H$_3$). **^{19}F{^{1}H} NMR** (**CD_3CN, 298 K, 282 MHz**): δ -72.88 (d, *J* = 707 Hz, P**F$_6$**). **^{31}P{^{1}H} NMR (CD_3CN, 298 K, 121 MHz):** δ -144.62 (sept, *J* = 707 Hz, **P**F$_6$). **ESI-MS (MeCN, calc. for [M–PF$_6$]$^+$):** 288.1342 (288.1343).

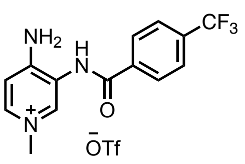

4-amino-1-methyl-3-(4-(trifluoromethyl)benzamido)pyridin-1-ium trifluoromethanesulfonate (2c). Under an N_2 atmosphere, compound **1c** (100 mg, 0.26 mmol, 1 eq) and MeOTf (143 µL, 1.3 mmol, 5 eq) were dissolved in dry CH_2Cl_2 (5 mL) and heated at 42 °C in a closed system for 16 h. After cooling to room temperature, Et_2O (50 mL) was added, and the precipitate was collected by filtration and dried thoroughly to yield **2c** as a white solid (94 mg, 80%).

^{1}H NMR (CD_3CN, 298 K, 300 MHz): δ 8.71 (bs, 1H, N**H**), 8.19–8.12 (m, 3H, C$\mathbf{H}_{PYA}$, 2×C$\mathbf{H}_{Ar}$), 7.90–7.87 (m, 3H, C$\mathbf{H}_{PYA}$, 2×C$\mathbf{H}_{Ar}$), 7.01 (d, *J* = 7.2 Hz, 1H, C$\mathbf{H}_{PYA}$), 6.66 (bs, 2H, N$\mathbf{H_2}$), 3.92 (s, 3H, NC$\mathbf{H_3}$). **^{13}C{^{1}H} NMR (CD_3CN, 298 K, 75 MHz):** δ 166.12 ($\mathbf{C}_{C=O}$), 155.70 ($\mathbf{C}_{PYA}$), 142.74 ($\mathbf{C}H_{PYA}$), 142.67 ($\mathbf{C}H_{PYA}$), 137.38 ($\mathbf{C}_{Ar}$), 133.44 ($\mathbf{C}_{Ar}$), 129.36 ($\mathbf{C}_{Ar}$), 129.27 ($\mathbf{C}H_{Ar}$), 126.25 (q, *J* = 3.9 Hz, $\mathbf{C}H_{Ar}$), 120.23 ($\mathbf{C}_{PYA}$), 111.04 ($\mathbf{C}H_{PYA}$), 45.80 (N$\mathbf{C}H_3$). **^{19}F{^{1}H} NMR** (**CD_3CN, 298 K, 282 MHz**): δ -63.57 (s), -79.35 (s). **ESI-MS (MeCN, calc. for [M–PF_6]$^+$):** 288.1342 (288.1343).

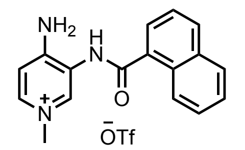

4-amino-3-(3,5-dimethoxybenzamido)-1-methylpyridin-1-ium trifluoromethanesulfonate (2d). Using the procedure described for **2c**, compound **2d** was prepared from **1d** (130 mg, 0.36 mmol, 1 eq) and MeOTf (196 μL, 1.8 mmol, 5 eq) and was isolated as a white solid (135 mg, 88%).

^{1}H NMR (CD_3CN, 298 K, 300 MHz): δ 8.51 (bs, 1H, N**H**), 8.44 (s, 1H, C$\mathbf{H}_{PYA}$), 8.41–8.38 (m, 1H, C$\mathbf{H}_{Ar}$), 8.11 (d, *J* = 8.4 Hz, 1H, C$\mathbf{H}_{Ar}$), 8.02–7.99 (m, 1H, C$\mathbf{H}_{Ar}$), 7.95 (dd, *J* = 7.1, 1.2 Hz, 1H, C$\mathbf{H}_{Ar}$), 7.89 (dd, *J* = 7.1, 1.8 Hz, 1H, C$\mathbf{H}_{PYA}$), 7.66–7.59 (m, 3H, 3×C$\mathbf{H}_{Ar}$), 7.04 (d, *J* = 7.1 Hz, 1H, C$\mathbf{H}_{PYA}$), 6.63 (bs, 2H, N$\mathbf{H_2}$), 3.96 (s, 3H, NC$\mathbf{H_3}$). **^{13}C{^{1}H} NMR (CD_3CN, 298 K, 75 MHz):** δ 169.48 ($\mathbf{C}_{C=O}$), 155.36 ($\mathbf{C}_{PYA}$), 142.68 ($\mathbf{C}H_{PYA}$), 142.37 ($\mathbf{C}H_{PYA}$), 134.75 ($\mathbf{C}_{Ar}$), 133.24 ($\mathbf{C}_{Ar}$), 132.71 ($\mathbf{C}H_{Ar}$), 131.13 ($\mathbf{C}_{Ar}$), 129.48 ($\mathbf{C}H_{Ar}$), 128.37 ($\mathbf{C}H_{Ar}$), 127.68 ($\mathbf{C}H_{Ar}$), 127.61 ($\mathbf{C}H_{Ar}$), 126.36 ($\mathbf{C}H_{Ar}$), 125.81 ($\mathbf{C}H_{Ar}$), 121.08 ($\mathbf{C}_{PYA}$), 111.51 ($\mathbf{C}H_{PYA}$), 46.32 (N$\mathbf{C}H_3$). **^{19}F{^{1}H} NMR** (**CD_3CN, 298 K, 282 MHz**): δ -79.35 (s). **ESI-MS (MeCN, calc. for [$C_{17}H_{16}N_3O$]$^+$):** 278.1280 (278.1288).

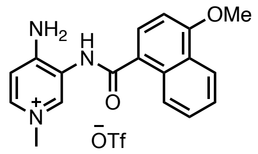

4-amino-3-(4-methoxy-1-naphthamido)-1-methylpyridin-1-ium trifluoromethanesulfonate (2e) Using the procedure described for **2c**, compound **2e** was obtained from **1e** (100 mg, 0.25 mmol, 1 eq) and MeOTf (140 μL, 1.3 mmol, 5 eq) as a white solid (85 mg, 73%).

^{1}H NMR (CD_3CN, 298 K, 300 MHz): δ 8.49 (d, *J* = 8.2 Hz, 1H, C$\mathbf{H}_{Ar}$), 8.41 (bs, 1H, N**H**), 8.38 (s, 1H, C$\mathbf{H}_{PYA}$), 8.32 (d, *J* = 8.2 Hz, 1H, C$\mathbf{H}_{Ar}$), 7.97 (d, *J* = 8.1 Hz, 1H, C$\mathbf{H}_{Ar}$), 7.88 (d, *J* = 7.2 Hz, 1H, C$\mathbf{H}_{PYA}$), 7.61 (m, C$\mathbf{H}_{Ar}$), 7.02 (dd, *J* = 7.6, 4.5 Hz, 2H, C$\mathbf{H}_{PYA}$, C$\mathbf{H}_{Ar}$), 6.62 (bs, 2H, N$\mathbf{H_2}$), 4.09 (s, 3H, OC$\mathbf{H_3}$), 3.95 (s, 3H, NC$\mathbf{H_3}$). **^{13}C{^{1}H} NMR (CD_3CN, 298 K, 75 MHz):** δ 169.31 ($\mathbf{C}_{C=O}$), 159.12 ($\mathbf{C}_{Ar}$), 155.50 ($\mathbf{C}_{PYA}$), 142.57 ($\mathbf{C}H_{PYA}$), 142.33 ($\mathbf{C}H_{PYA}$), 132.65 ($\mathbf{C}_{Ar}$), 129.48 ($\mathbf{C}H_{Ar}$), 128.80 ($\mathbf{C}H_{Ar}$), 126.99 ($\mathbf{C}H_{Ar}$), 126.54 ($\mathbf{C}_{Ar}$), 126.42 ($\mathbf{C}H_{Ar}$), 124.96 ($\mathbf{C}_{Ar}$), 123.04 ($\mathbf{C}H_{Ar}$), 121.42 ($\mathbf{C}_{PYA}$), 111.41 ($\mathbf{C}H_{PYA}$), 103.75 ($\mathbf{C}H_{Ar}$), 56.77 (O$\mathbf{C}H_3$), 46.29 (N$\mathbf{C}H_3$). **^{19}F{^{1}H} NMR** (**CD_3CN, 298 K, 282 MHz**): δ -79.34 (s). **ESI-MS (MeCN, calc. for $C_{18}H_{18}N_3O_2$ [M]$^+$):** 308.1385 (308.1394).

S.2 Analytical data of complexes 3 and 4

Table S1. Comparison of ^{1}H NMR shifts (ppm) of the aromatic *p*-cymene protons depending on the solvent for complexes **3a–e**.

entry	complex	$\delta_{H,cym}$ (CD_2Cl_2)	$\delta_{H,cym}$ (CD_3OD)
1	**3a**	5.28 (2H), 5.08 (2H)	5.39 (2H), 5.08 (2H)
2	**3b**	5.36 (2H), 5.14 (2H)	5.46 (2H), 5.10 (2H)
3	**3c**	5.30 (2H), 5.11 (2H)	5.42 (2H), 4.99 (2H)
4	**3d**	5.07 (1H), 4.89 (1H), 4.60 (2H)	5.28 (1H), 4.85 (1H), 4.55 (2H)
5	**3e**	5.15 (1H), 5.01 (1H), 4.77 (2H)	5.33 (1H), 4.97 (1H), 4.77 (2H)

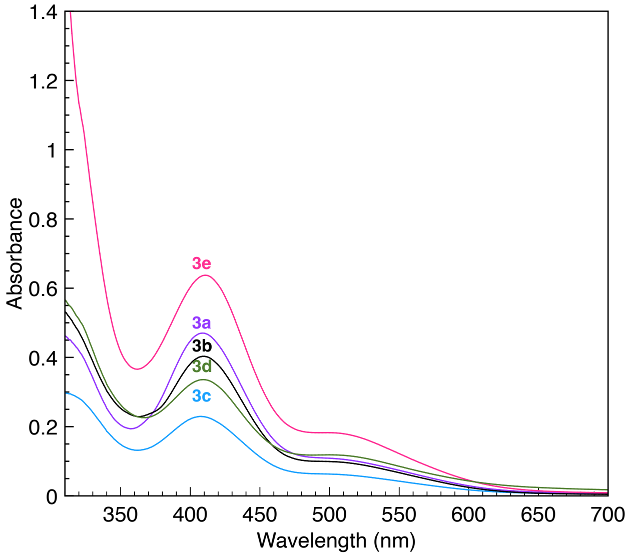

Figure S1. UV-Vis absorption spectra in MeOH of Ru(II) PYA complexes **3a–e** with various acyl substituents.

Table S2. Absorption maxima λ_{max} (nm) and extinction coefficients ε ($M^{-1}cm^{-1}$) for complexes **3a–e** in CH_2Cl_2 and CH_3OH.

entry	complex	solvent	λ_{max} (nm) [a]	ε (10^4 M^{-1} cm^{-1}) [b]
1	**3a**	CH_2Cl_2	409	1.3
2	**3a**	CH_3OH	409	0.9
3	**3b**	CH_2Cl_2	410	1.1
4	**3b**	CH_3OH	409	0.8
5	**3c**	CH_2Cl_2	409	1.5
6	**3c**	CH_3OH	407	0.5
7	**3d**	CH_2Cl_2	411	1.3
8	**3d**	CH_3OH	409	0.7
9	**3e**	CH_2Cl_2	411	1.5
10	**3e**	CH_3OH	411	1.3

[a] 0.1 nm wavelength accuracy; [b] <10% esd. Measurements were generally made from a 10 mM stock solution of complex **3a–e** in the corresponding solvent and subsequent dilution to 0.05 mM.

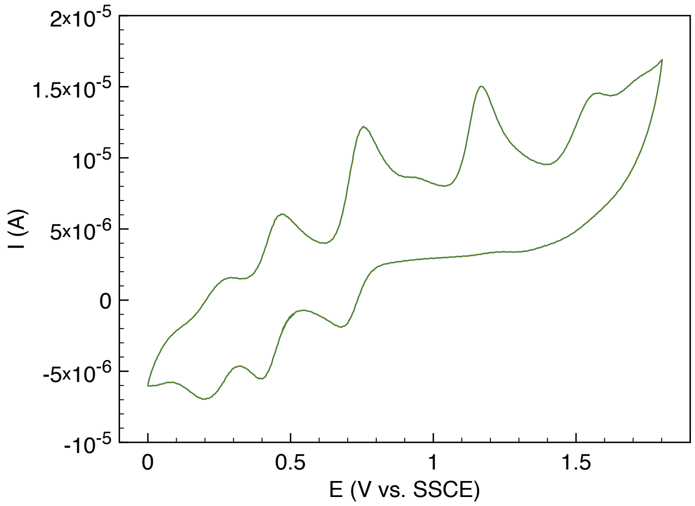

Figure S2. Cyclic voltammetry measurement of complex **3a**. Potential vs. Fc^+/Fc couple in CH_3CN, $(Bu_4N)PF_6$ as supporting electrolyte.

S.2.1 Synthesis and spectroscopic data of complexes 4a–d

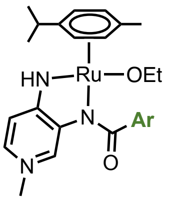

In a NMR tube, compound **3a–d** (1 eq) was suspended in MeOD (0.5 mL) and EtONa (2 eq) was added at rt. The reaction was analyzed by ^{1}H NMR spectroscopy.

Complex 4a – ^{1}H NMR (CD_3OD, 298 K, 300 MHz): δ 8.91 (s, 1H, C**H**$_{PYA}$), 7.99 (bs, 2H, C**H**$_{Ar}$), 7.60–7.48 (m, 3H, C**H**$_{PYA,}$), 7.28 (d, *J* = 6.7 Hz, 1H, C**H**$_{Ar}$), 6.63 (d, *J* = 7.0 Hz, 1H, C**H**$_{PYA}$), 5.10 (d, *J* = 5.7 Hz, 2H, C**H**$_{cym}$), 4.38 (d, *J* = 5.6 Hz, 2H, C**H**$_{cym}$), 3.70 (s, 3H, NC**H$_3$**), 3.61 (q, *J* = 7.0 Hz, 4H, OC**H$_2$**CH_3), 2.38 (septet, *J* = 6.7 Hz, 1H, C**H**$(CH_3)_2$), 1.96 (s, 3H, cym-C**H$_3$**), 1.18 (t, *J* = 7.0 Hz, 6H, OCH_2C**H$_3$**), 1.04 (d, *J* = 6.7 Hz, 6H, CH(C**H$_3$**)$_2$).

Complex 4b – ^{1}H NMR (CD_3OD, 298 K, 300 MHz): δ 8.96 (s, 1H, C**H**$_{PYA}$), 7.16 (d, *J* = 2.3 Hz, 1H, C**H**$_{Ar}$), 7.12 (m, 1H, C**H**$_{PYA,}$), 6.59 (t, *J* = 2.3 Hz, 1H, C**H**$_{Ar}$), 6.38 (d, *J* = 7.1 Hz, 1H, C**H**$_{PYA}$), 5.14 (d, *J* = 5.8 Hz, 2H, C**H**$_{cym}$), 4.04 (d, *J* = 5.6 Hz, 2H, C**H**$_{cym}$), 3.89 (s, 3H, NC**H$_3$**), 3.62 (q, *J* = 7.0 Hz, 4H, OC**H$_2$**CH_3), 2.34 (septet, *J* = 6.9 Hz,, 1H, C**H**$(CH_3)_2$), 1.88 (s, 3H, cym-C**H$_3$**), 1.18 (t, *J* = 7.0 Hz, 6H, OCH_2C**H$_3$**), 1.00 (d, *J* = 6.9 Hz, 6H, CH(C**H$_3$**)$_2$).

Complex 4c – ^{1}H NMR (CD_3OD, 298 K, 300 MHz): δ 9.07 (s, 1H, C**H**$_{PYA}$), 8.15 (d, *J* = 8.0 Hz, 2H, C**H**$_{Ar}$), 7.79 (d, *J* = 8.0 Hz, 2H, C**H**$_{Ar}$), 7.14 (dd, *J* = 7.1, 1.9 Hz, 1H, C**H**$_{PYA}$), 6.40 (d, *J* = 7.1 Hz, 1H, C**H**$_{PYA}$), 4.99 (d, *J* = 5.7 Hz, 2H, C**H**$_{cym}$), 4.03 (d, *J* = 5.6 Hz, 2H, C**H**$_{cym}$), 3.63 (s, 3H, NC**H$_3$**), 3.61 (q, *J* = 7.0 Hz, 4H, OC**H$_2$**CH_3), 2.34 (septet, *J* = 6.9 Hz, 1H, C**H**$(CH_3)_2$), 1.89 (s, 3H, cym-C**H$_3$**), 1.18 (t, *J* = 7.0 Hz, 6H, OCH_2C**H$_3$**), 0.99 (d, *J* = 6.9 Hz, 6H, CH(C**H$_3$**)$_2$).

Complex 4d – ^{1}H NMR (CD_3OD, 298 K, 300 MHz): δ 9.26 (s, 1H, C**H**$_{PYA}$), 8.35–8.24 (m, 1H, C**H**$_{Ar}$), 7.98 (dd, *J* = 8.2, 5.7 Hz, 2H, C**H**$_{Ar}$), 7.87–7.78 (m, 1H, C**H**$_{Ar}$), 7.70–7.53 (m, 3H, C**H**$_{Ar}$), 7.17 (dd, *J* = 7.1, 1.9 Hz, 1H, C**H**$_{PYA}$), 6.42 (d, *J* = 7.1 Hz, 1H, C**H**$_{PYA}$), 5.28 (s, 1H, C**H**$_{cym}$), 4.60 (s, 1H, C**H**$_{cym}$), 4.37 (s, 1H, C**H**$_{cym}$), 3.66 (s, 3H, NC**H$_3$**), 3.61 (q, *J* = 7.0 Hz, 4H, OC**H$_2$**CH_3), 2.27 (septet, *J* = 6.9 Hz, 1H, C**H**$(CH_3)_2$), 1.89 (s, 3H, cym-C**H$_3$**), 1.18 (t, *J* = 7.0 Hz, 6H, OCH_2C**H$_3$**), 0.95 (br s, 6H, CH(C**H$_3$**)$_2$).

S.3 Catalytic data

S.3.1 General catalytic procedure for catalytic transfer hydrogenation

In a 10 mL round bottom flask, the substrate (0.5 mmol), complex **3** (1 mol%), and 1,3,5-trimethoxybenzene (10 mol%) as internal standards were dissolved in EtOH (5 mL) and the solution was degassed with N_2 for 10 min. The catalytic run was started with the injection of K_2CO_3 (5 mol%, 2 M solution in H_2O) and the tube was placed in a pre-heated oil bath (25 °C). The reaction was monitored by ^1H NMR spectroscopy (300 MHz, 298 K), with reaction aliquots (ca. 0.1 mL) taken at set times under N_2 and dissolved in $CDCl_3$ (0.5 mL) to determine the spectroscopic conversion and yields relative to the internal standard. Representative time-yield profiles for complexes **3b** and **3d** are shown in Fig. S3a and S3b.

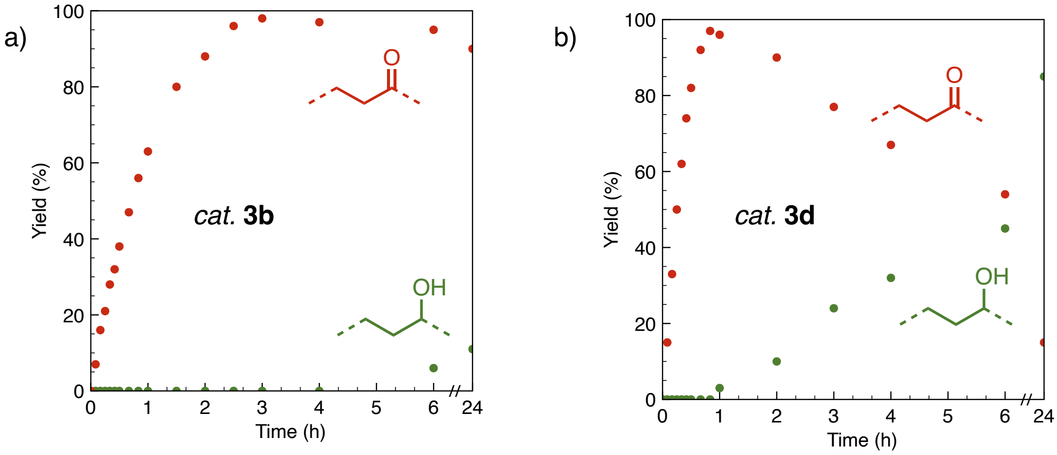

Figure S3. Time-conversion profiles of the Ru-catalyzed transfer hydrogenation of *trans*-chalcone to form subsequently 1,3-diphenylpropan-1-one (red dots) and 1,3-diphenylpropan-1-ol (green dots) using EtOH as hydrogen source using a) complex **3b** as catalyst precursor, and b) complex **3d**.

S.3.2 Determination of rate constants

Under the general catalytic procedure, the initial rate constants $k_{C=C}$ and $k_{C=O}$ for the successive C=C and C=O bond catalytic transfer hydrogenation of *trans*-chalcone for complexes **3a–e** were extracted from the linear regimes (Figures S4-S8 1,3-diphenylpropan-1-one in red, 1,3-diphenylpropan-1-ol in green). Numeric evaluation of the primary process, the C=C bond transfer hydrogenations are compiled in Tables S3–S7. For the second process, that is, the C=O bond transfer hydrogenation, the initial time t = 0 was set at >97% conversion of *trans*-chalcone to 1,3-diphenylpropan-1-one, *i.e.* after 50 min for complex **3d**, after 1h for complexes **3a** and **3e**, after 2h for complex **3c**, and after 3h for complex **3b** (Tables S8–S12).

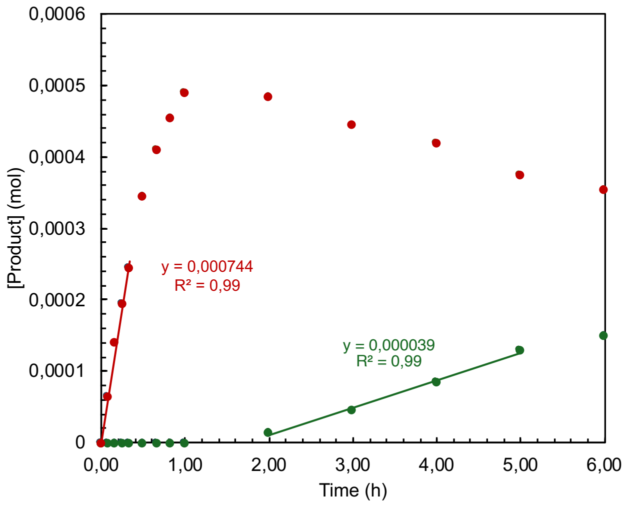

Figure S4. Determination of rate constants $k_{C=C}$ and $k_{C=O}$ for the consecutive C=C and C=O transfer hydrogenation of *trans*-chalcone catalyzed by complex **3a**.

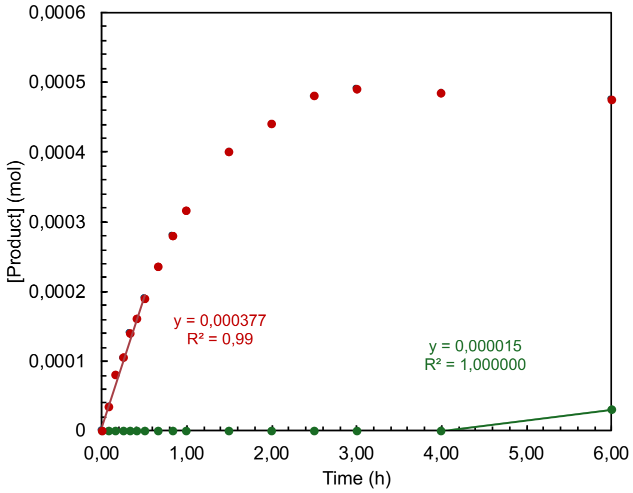

Figure S5. Determination of rate constants $k_{C=C}$ and $k_{C=O}$ for the consecutive C=C and C=O transfer hydrogenation of *trans*-chalcone catalyzed by complex **3b**.

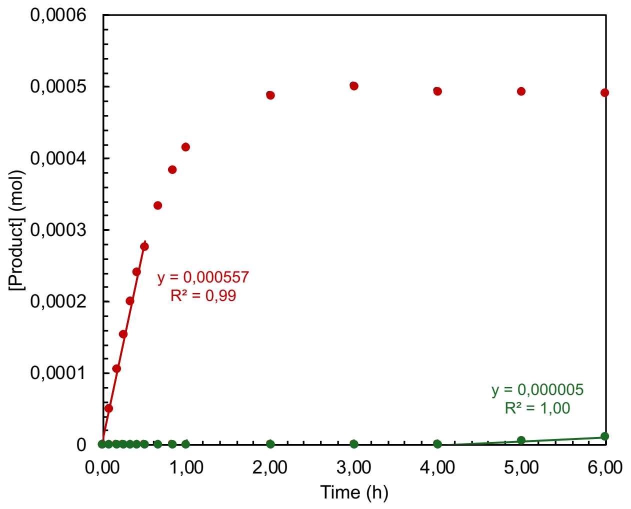

Figure S6. Determination of rate constants $k_{C=C}$ and $k_{C=O}$ for the consecutive C=C and C=O transfer hydrogenation of *trans*-chalcone catalyzed by complex **3c**.

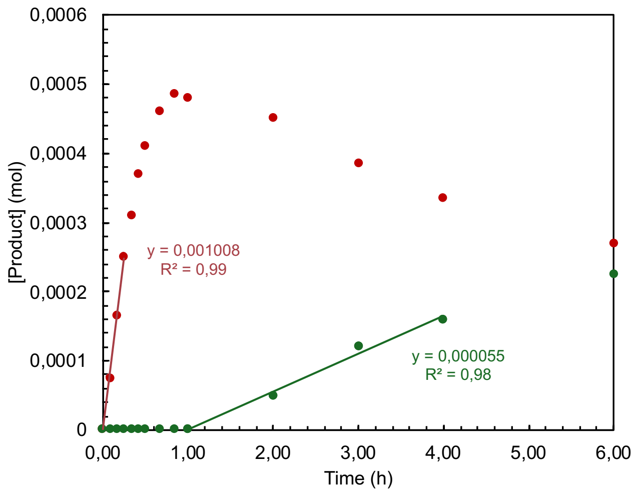

Figure S7. Determination of rate constants $k_{C=C}$ and $k_{C=O}$ for the consecutive C=C and C=O transfer hydrogenation of *trans*-chalcone catalyzed by complex **3d**.

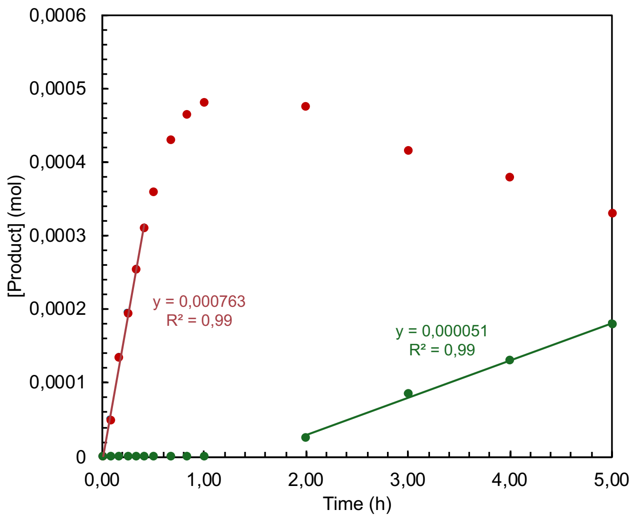

Figure S8. Determination of rate constants $k_{C=C}$ and $k_{C=O}$ for the consecutive C=C and C=O transfer hydrogenation of *trans*-chalcone catalyzed by complex **3e**.

C=C transfer hydrogenation kinetcis

Table S3. Data for the determination of $k_{C=C}$ and max $TOF_{C=C}$ of the C=C transfer hydrogenation of *trans*-chalcone catalyzed by complex **3a**.

Time (h)	[Product]	$k_{C=C}$ (h^{-1})	max $TOF_{C=C}$ (h^{-1})
0.00	0	74×10^{-5}<br>R^2 = 0.99	170<br>(at 28% conversion,<br>t = 0.17 h)
0.08	0.000065		
0.17	0.000140		
0.25	0.000195		
0.33	0.000245		

Table S4. Data for the determination of $k_{C=C}$ and max $TOF_{C=C}$ of the C=C transfer hydrogenation of *trans*-chalcone catalyzed by complex **3b**.

Time (h)	[Product]	$k_{C=C}$ (h^{-1})	max $TOF_{C=C}$ (h^{-1})
0.00	0	38×10^{-5}<br>R^2 = 0.99	100<br>(at 17% conversion,<br>t = 0.17 h)
0.08	0.000035		
0.17	0.000080		
0.25	0.000105		
0.33	0.000140		
0.42	0.000160		
0.50	0.000190		

Table S5. Data for the determination of $k_{C=C}$ and max $TOF_{C=C}$ of the C=C transfer hydrogenation of *trans*-chalcone catalyzed by complex **3c**.

Time (h)	[Product]	$k_{C=C}$ (h^{-1})	max $TOF_{C=C}$ (h^{-1})
0.00	0	56×10^{-5} $R^2 = 0.99$	130 (at 21% conversion, t = 0.17 h)
0.08	0.000050		
0.17	0.000105		
0.25	0.000153		
0.33	0.000200		
0.42	0.000240		
0.50	0.000275		

Table S6. Data for the determination of $k_{C=C}$ and max $TOF_{C=C}$ of the C=C transfer hydrogenation of *trans*-chalcone catalyzed by complex **3d**.

Time (h)	[Product]	$k_{C=C}$ (h^{-1})	max $TOF_{C=C}$ (h^{-1})
0.00	0	101×10^{-5} $R^2 = 0.99$	200 (at 50% conversion, t = 0.25 h)
0.08	0.000075		
0.17	0.000165		
0.25	0.000250		

Table S7. Data for the determination of $k_{C=C}$ and max $TOF_{C=C}$ of the C=C transfer hydrogenation of *trans*-chalcone catalyzed by complex **3e**.

Time (h)	[Product]	$k_{C=C}$ (h^{-1})	max $TOF_{C=C}$ (h^{-1})
0.00	0	76×10^{-5} $R^2 = 0.99$	160 (at 27% conversion, t = 0.17 h)
0.08	0.000050		
0.17	0.000135		
0.25	0.000195		
0.33	0.000255		
0.42	0.000310		

C=O transfer hydrogenation kinetcis

Table S8. Data for the determination of $k_{C=O}$ and max $TOF_{C=O}$ of the *in situ* C=O transfer hydrogenation of 1,3-diphenylpropan-1-one catalyzed by complex **3a**.

Time (h)	[Product]	$k_{C=O}$ (h^{-1})	max $TOF_{C=O}$ (h^{-1})
0	0	3.9×10^{-5} $R^2 = 0.99$	7 (at 26% conversion, t = 4 h)
1	0.000015		
2	0.000045		
3	0.000085		
4	0.000130		

Table S9. Data for the determination of $k_{C=O}$ and max $TOF_{C=O}$ of the *in situ* C=O transfer hydrogenation of 1,3-diphenylpropan-1-one catalyzed by complex **3b**.

Time (h)	[Product]	$k_{C=O}$ (h^{-1})	max $TOF_{C=O}$ (h^{-1})
0	0	1.5×10^{-5} [a]	2 (at 6% conversion, t = 3 h)
1	0		
3	0.000030		

[a]Not sufficient data points for an accurate linear correlation.

Table S10. Data for the determination of $k_{C=O}$ and max $TOF_{C=O}$ of the *in situ* C=O transfer hydrogenation of 1,3-diphenylpropan-1-one catalyzed by complex **3c**.

Time (h)	[Product]	$k_{C=O}$ (h^{-1})	max $TOF_{C=O}$ (h^{-1})
0	0	0.5×10^{-5} $R^2 = 1.00$	1 (at 2% conversion, t = 4 h)
1	0		
2	0		
3	0.000005		
4	0.000010		

Table S11. Data for the determination of $k_{C=O}$ and max $TOF_{C=O}$ of the *in situ* C=O transfer hydrogenation of 1,3-diphenylpropan-1-one catalyzed by complex **3d**.

Time (h)	[Product]	$k_{C=O}$ (h^{-1})	max $TOF_{C=O}$ (h^{-1})
0	0	5.5×10^{-5} $R^2 = 0.99$	11 (at 24% conversion, t = 2.17 h)
1.17	0.000050		
2.17	0.000120		
3.17	0.000160		

Table S12. Data for the determination of $k_{C=O}$ and max $TOF_{C=O}$ of the *in situ* C=O transfer hydrogenation of 1,3-diphenylpropan-1-one catalyzed by complex **3e**.

Time (h)	[Product]	$k_{C=O}$ (h^{-1})	max $TOF_{C=O}$ (h^{-1})
0	0		
1	0,000025		9
2	0,000085	5.1×10^{-5} $R^2 = 0.99$	(at 17% conversion,
3	0,00013		t = 2 h)
4	0,00018		

S.3.3 Characterization of transfer hydrogenation products 6a-6e

General procedure for product characterization: Either after substrate consumption or after a maximum of 24 h, the reaction mixture was cooled down to room temperature if necessary and concentrated under reduced pressure. All products were isolated before characterization unless stated otherwise. Product isolation was performed by concentration of the crude mixture followed by flash column chromatography (SiO_2, hexane/EtOAc) using Isolera One Flash Chromatography Instrument. The NMR data are consistent with the literature.[S4]

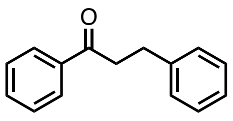

1,3-diphenylpropan-1-one (6a). The crude product was purified by flash column chromatography (SiO_2, hexane/EtOAc 95:5). **^{1}H NMR (300 MHz, 298 K, $CDCl_3$):** δ 7.96 (m, 2H), 7.56 (t, J = 7.3, 1H), 7.45 (t, J = 7.2, 2H), 7.33–7.18 (m, 5H), 3.31 (t, J = 7.7 Hz, 2H), 3.07 (t, J = 8.0 Hz, 2H). **$^{13}C\{^1H\}$ NMR ($CDCl_3$, 298 K, 75 MHz):** δ 199.37, 141.44, 137.02, 133.20, 128.75, 128.68, 128.57, 128.19, 126.28, 40.60, 30.29.

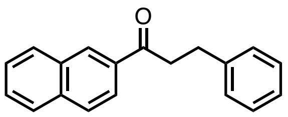

1-(naphthalen-2-yl)-3-phenylpropan-1-one (6b). The crude product was purified by flash column chromatography (SiO_2, hexane/EtOAc 9:1). **^{1}H NMR (300 MHz, 298 K, $CDCl_3$):** δ 8.41 (s, 1H), 8.00 (dd, J = 8.6, 1.8 Hz, 1H), 7.90–7.81 (m, 3H), 7.58–7.47 (m, 2H), 7.31–7.24 (m, 4H), 7.20–7.15 (m, 1H), 3.38 (t, J = 8.1 Hz, 2H), 3.09 (t, J = 8.2 Hz, 2H). **$^{13}C\{^1H\}$ NMR ($CDCl_3$, 298 K, 75 MHz):** δ 199.19, 141.45, 135.66, 134.27, 132.61, 129.77, 129.63, 128.65, 128.57, 128.54, 128.52, 127.86, 126.85, 126.26, 123.93, 40.62, 30.36.

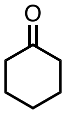

Cyclohexanone (6c). The crude product was purified by flash column chromatography (SiO_2, hexane/EtOAc 9:1). **^{1}H NMR (300 MHz, 298 K, $CDCl_3$):** δ 2.31 (t, J = 6.7 Hz, 4H), 1.87–1.79 (m, 4H), 1.73–1.65 (m, 2H). **$^{13}C\{^1H\}$ NMR ($CDCl_3$, 298 K, 75 MHz):** δ 212.30, 42.05, 27.10, 25.07.

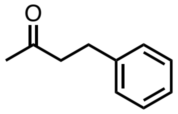

4-phenylbutan-2-one (6d). The crude product was purified by flash column chromatography (SiO_2, hexane/EtOAc 9:1). **^{1}H NMR (300 MHz, 298 K, $CDCl_3$):** δ 7.26–7.21 (m, 2H), 7.16–7.12 (m, 3H), 2.85 (t, *J* = 7.5 Hz, 2H), 2.71 (t, *J* = 7.2 Hz, 2H), 2.09 (s, 3H). **$^{13}C\{^1H\}$ NMR ($CDCl_3$, 298 K, 75 MHz):** δ 207.98, 141.08, 128.57, 128.37, 126.19, 45.23, 30.13, 29.81.

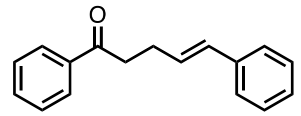

1,5-diphenylpent-4-en-1-one (6e). The crude product was purified by flash column chromatography (silica gel, hexane/EtOAc 9:1). **^{1}H NMR (300 MHz, 298 K, $CDCl_3$):** δ 8.00–7.97 (m, 2H), 7.57 (t, *J* = 7.4, 1H), 7.47 (t, *J* = 7.0 Hz, 2H), 7.37–7.26 (m, 4H), 7.20 (t, *J* = 7.4 Hz, 1H), 6.48 (d, *J* = 15.9 Hz, 1H), 6.30 (dt, *J* = 15.8, 6.8 Hz, 1H), 3.17 (t, *J* = 7.5 Hz, 2H), 2.67 (q, *J* = 6.7 Hz, 2H). **$^{13}C\{^1H\}$ NMR ($CDCl_3$, 298 K, 75 MHz):** δ 199.48, 137.62, 137.09, 133.20, 130.96, 129.28, 128.77, 128.64, 128.20, 127.22, 126.18, 38.43, 27.66.

S5 Crystal structure determination

A crystal of **3b** immersed in parabar oil was mounted at ambient conditions and transferred into the stream of nitrogen (100 K). All measurements were made on a *RIGAKU Synergy S* area-detector diffractometer using mirror optics monochromated Cu $K\alpha$ radiation (λ = 1.54184 Å). The unit cell constants and an orientation matrix for data collection were obtained from a least-squares refinement of the setting angles of reflections in the range 2.789° < θ < 78.282°. A total of 9116 frames were collected using ω scans, with 0.3 second exposure time (2 s for high-angle reflections), a rotation angle of 0.5° per frame, a crystal-detector distance of 31.0 mm, at T = 173.00(10) K. Data reduction was performed using the *CrysAlisPro* program. The intensities were corrected for Lorentz and polarization effects, and an absorption correction based on the multi-scan method using SCALE3 ABSPACK in *CrysAlisPro* was applied. Data collection and refinement parameters are given in Table S13. The structure was solved by intrinsic phasing using *SHELXT*,[S5] which revealed the positions of all non-hydrogen atoms of the title compound. All non-hydrogen atoms were refined anisotropically. H-atoms were assigned in geometrically calculated positions and refined using a riding model where each H-atom was assigned a fixed isotropic displacement parameter with a value equal to 1.2Ueq of its parent atom (1.5Ueq for methyl groups), except for that attached to N1, where the H atom was located from the difference density map and had its position and isotropic displacement parameter refined freely. Refinement of the structure was carried out on F^2 using full-matrix least-squares procedures, which minimized the function $\Sigma w(Fo^2–Fc^2)^2$. The weighting scheme was based on counting statistics and included a factor to downweight the intense reflections. All calculations were performed using the *SHELXL-2014/7*[S6]

program in OLEX2.[S7] Disorder model was used for parts of the structure where the occupancies of each disorder component was refined through the use of a free variable. The sum of equivalent components was constrained to 1, i.e. 100%. Crystallographic data for this structure have been deposited with the Cambridge Crystallographic Data Centre (CCDC) as supplementary publication number 2422796.

Table S13. Selected crystallographic and refinement data.

	Compound **3b**
CCDC No.	2422796
Empirical formula	$C_{25}H_{30}F_6N_3O_3PRu$
Formula weight	666.56
Temperature/K	173.00(10)
Crystal system	triclinic
Space group	P-1
a/Å	8.77993(9)
b/Å	9.12402(11)
c/Å	18.0299(2)
α/°	81.6256(10)
β/°	80.8187(9)
γ/°	73.6818(9)
Volume/Å^3	1360.63(3)
Z	2
ρ_{calc}g/cm^3	1.627
μ/mm^{-1}	5.889
F(000)	676.0
Crystal size/mm^3	0.243 × 0.152 × 0.076
Radiation	Cu Kα (λ = 1.54184)
2Θ range for data collection/°	4.994 to 149
Index ranges	-10 ≤ h ≤ 10, -11 ≤ k ≤ 11, -22 ≤ l ≤ 22
Reflections collected	48200
Independent reflections	5503 [R_{int} = 0.0294, R_{sigma} = 0.0135]
Data/restraints/parameters	5503/120/455
Goodness-of-fit on F^2	1.108
Final R indexes [I>=2σ (I)]	R_1 = 0.0254, wR_2 = 0.0609
Final R indexes [all data]	R_1 = 0.0257, wR_2 = 0.0611
Largest diff. peak/hole / e Å^{-3}	0.58/-0.63

S6 NMR spectra of all compounds

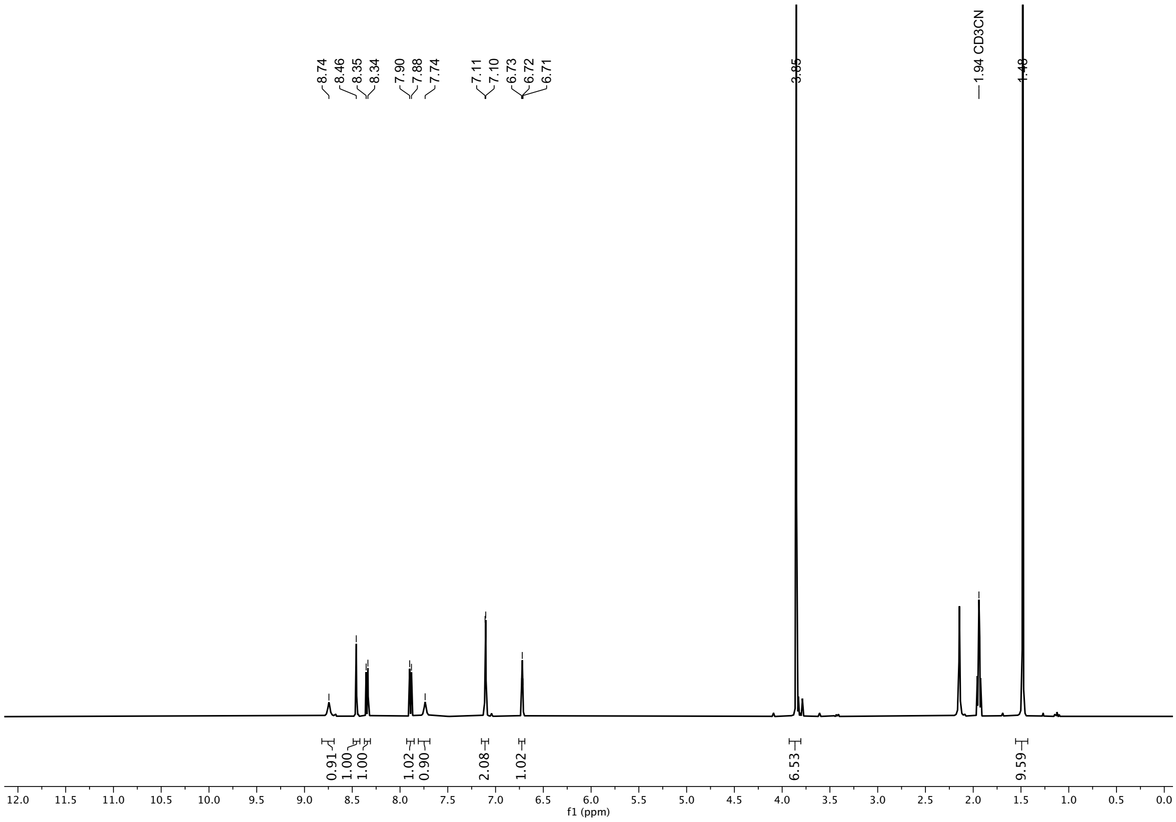

Figure S9. Solution ^{1}H NMR spectrum (CD_3CN, 298 K, 300 MHz) of **1b**.

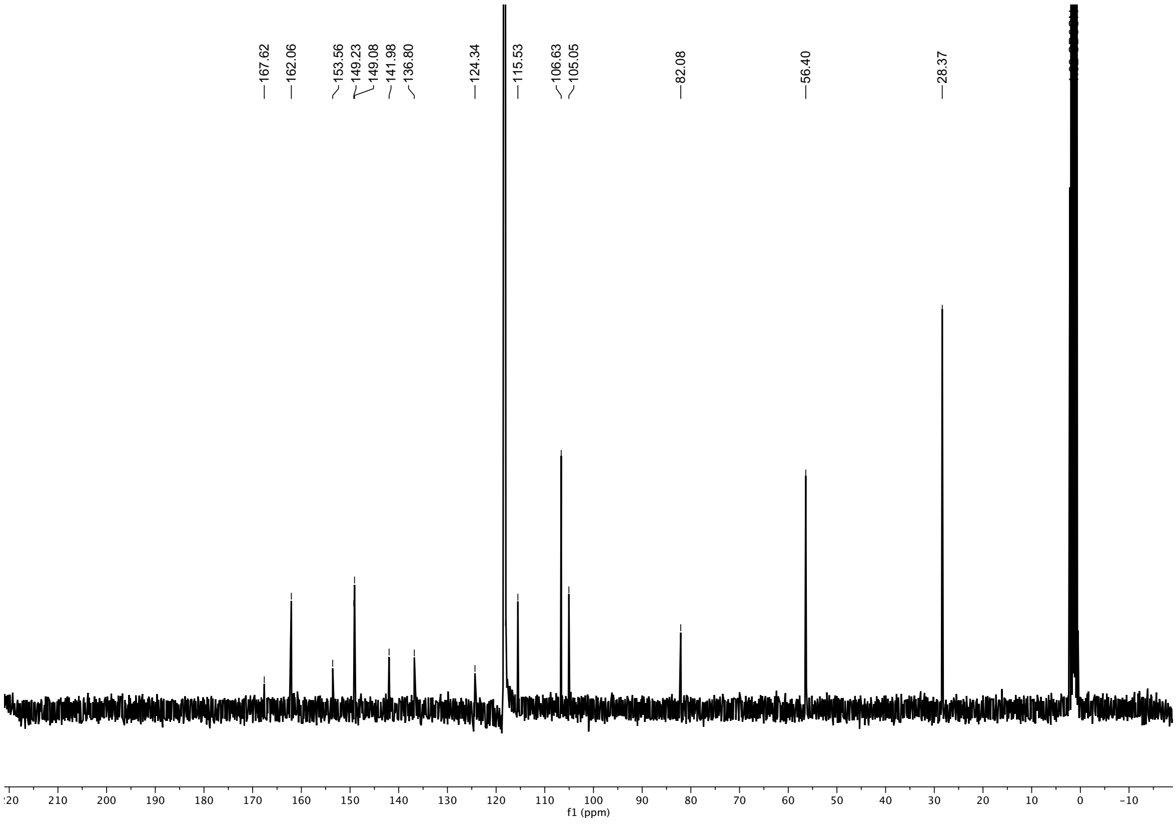

Figure S10. Solution $^{13}C\{^{1}H\}$ NMR spectrum (CD_3CN, 298 K, 75 MHz) of **1b**.

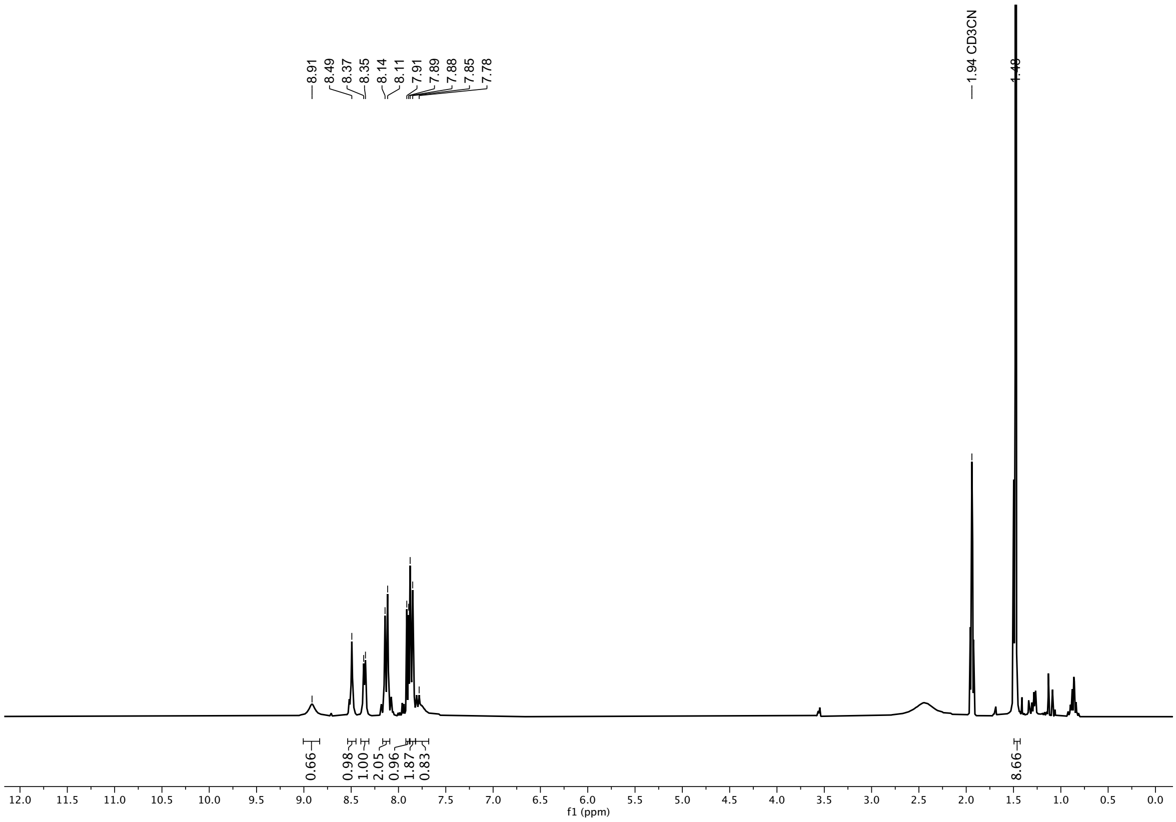

Figure S11. Solution ^{1}H NMR spectrum (CD_3CN, 298 K, 300 MHz) of **1c**.

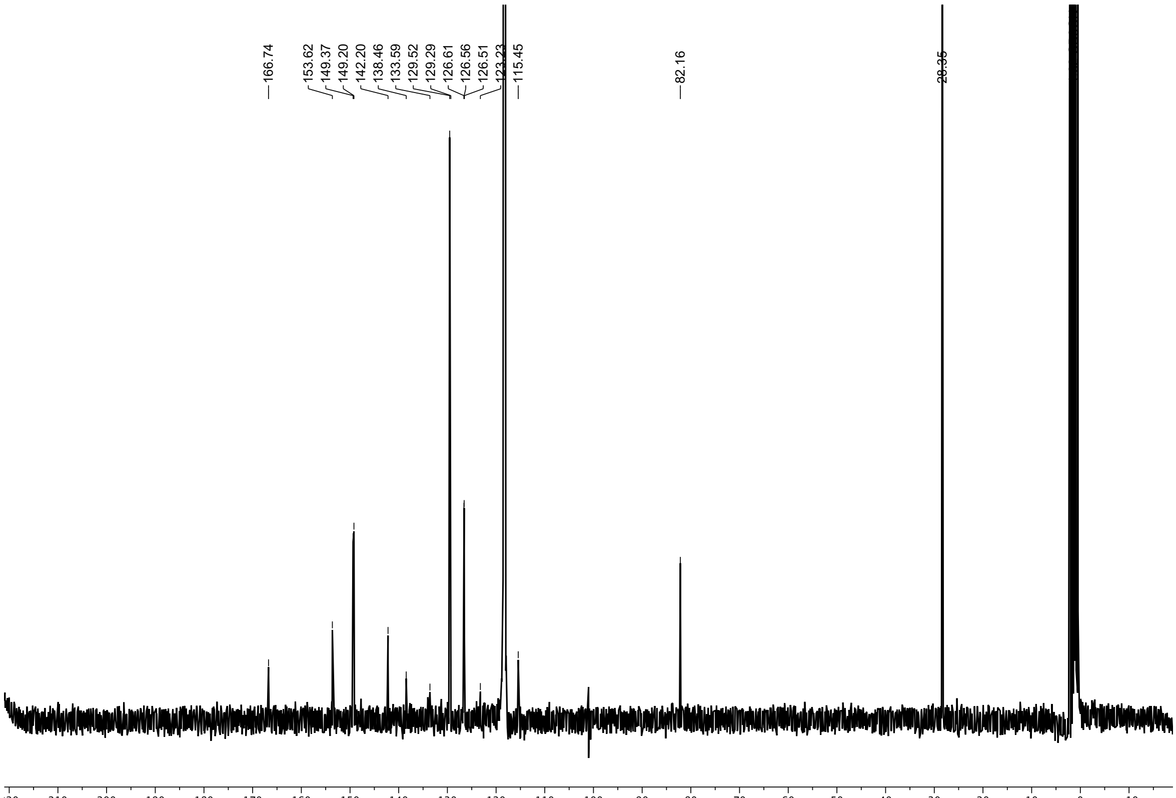

Figure S12. Solution $^{13}C\{^1H\}$ NMR spectrum (CD_3CN, 298 K, 75 MHz) of **1c**.

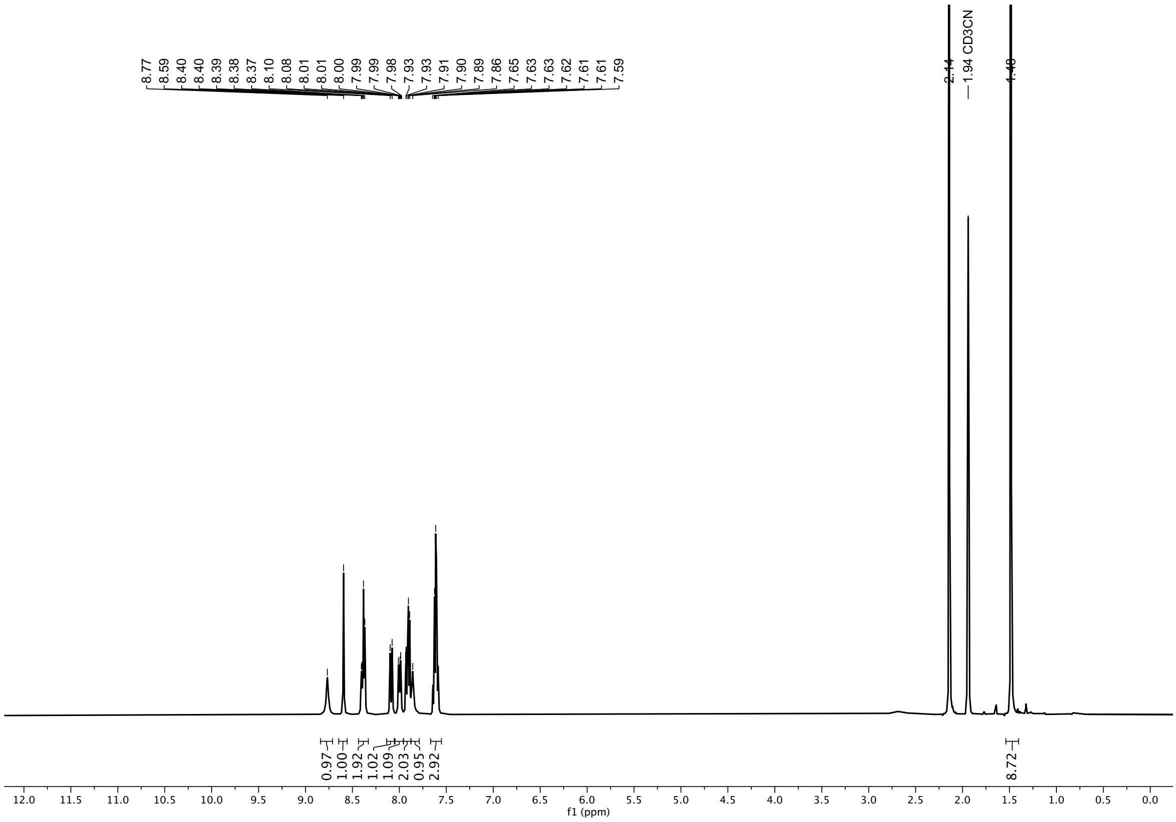

Figure S13. Solution ^{1}H NMR spectrum (CD_3CN, 298 K, 300 MHz) of **1d**.

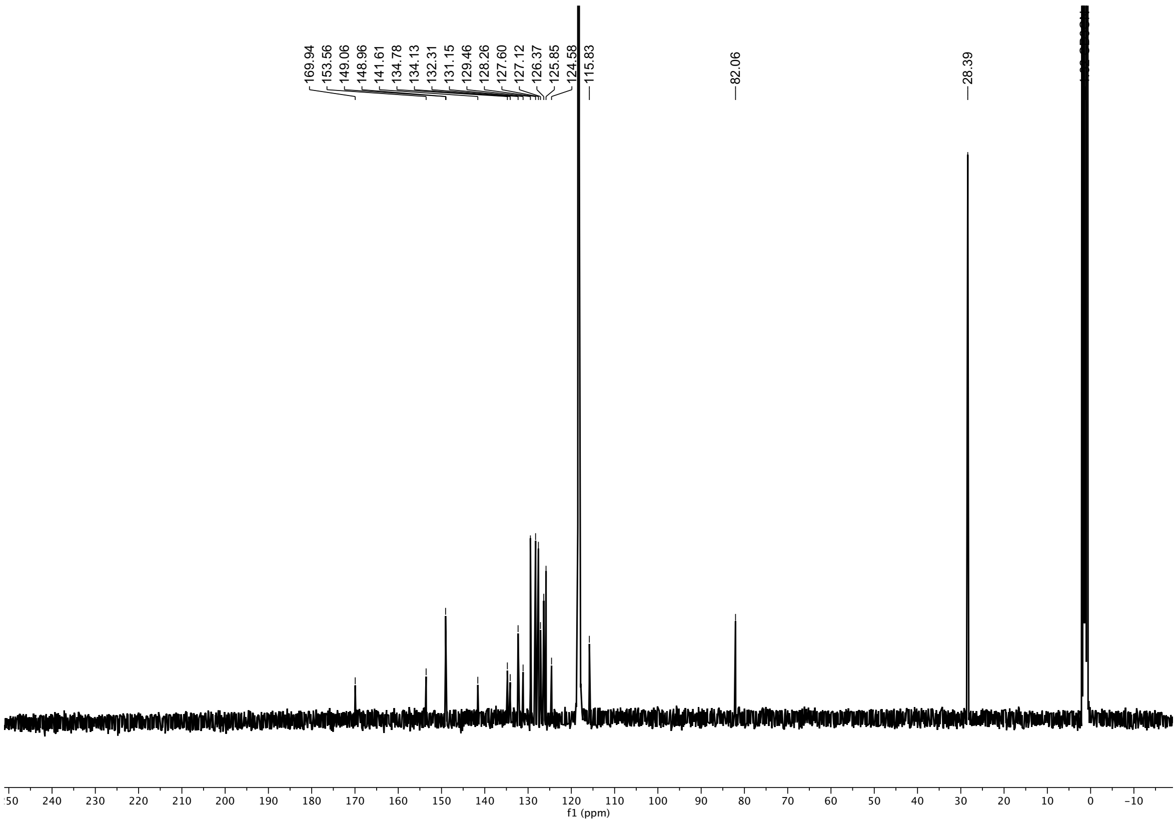

Figure S14. Solution $^{13}C\{^{1}H\}$ NMR spectrum (CD_3CN, 298 K, 75 MHz) of **1d**.

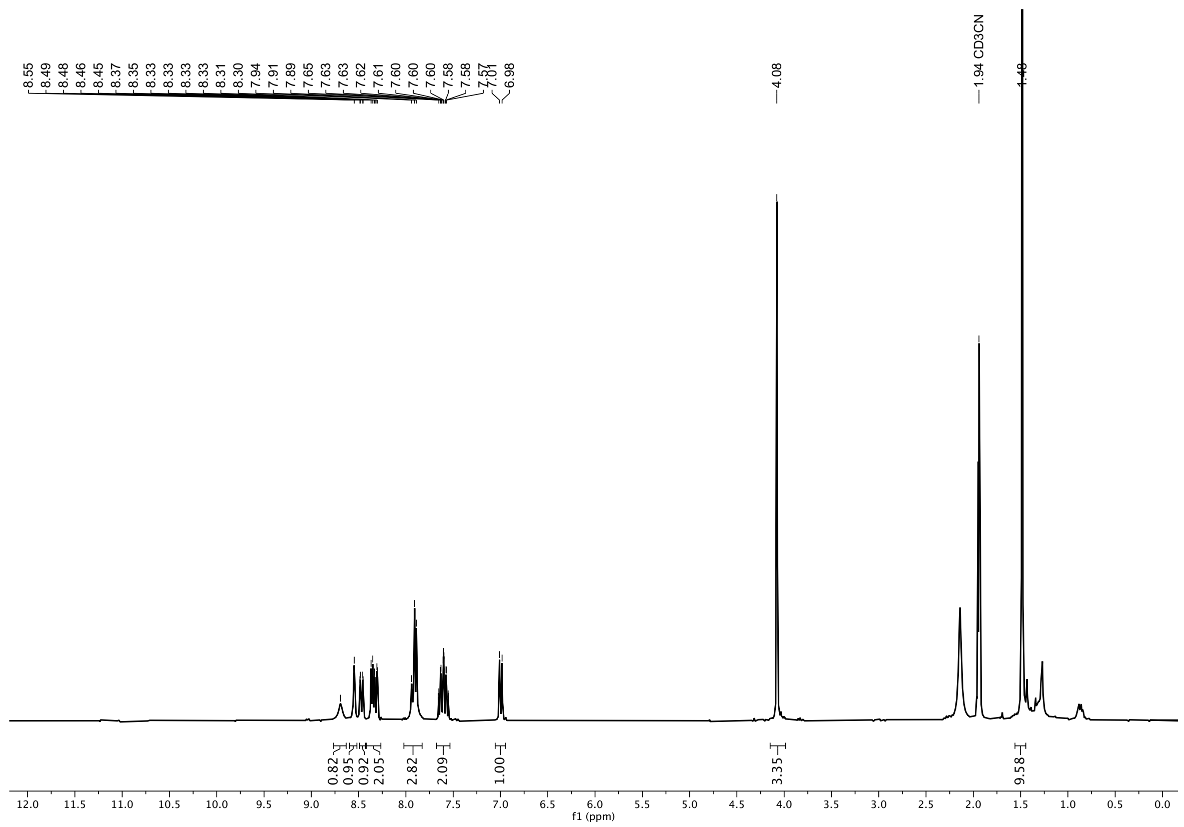

Figure S15. Solution ^{1}H NMR spectrum (CD_3CN, 298 K, 300 MHz) of **1e**.

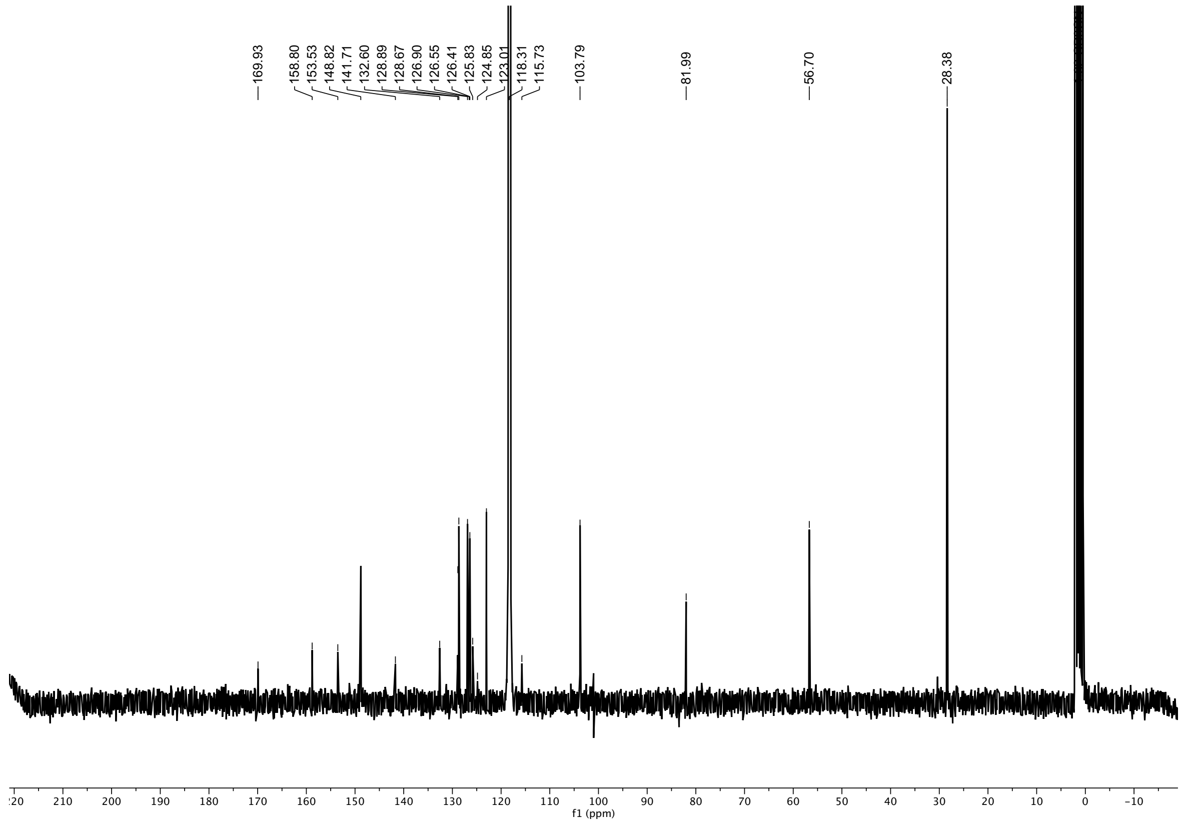

Figure S16. Solution $^{13}C\{^1H\}$ NMR spectrum (CD_3CN, 298 K, 75 MHz) of **1e**.

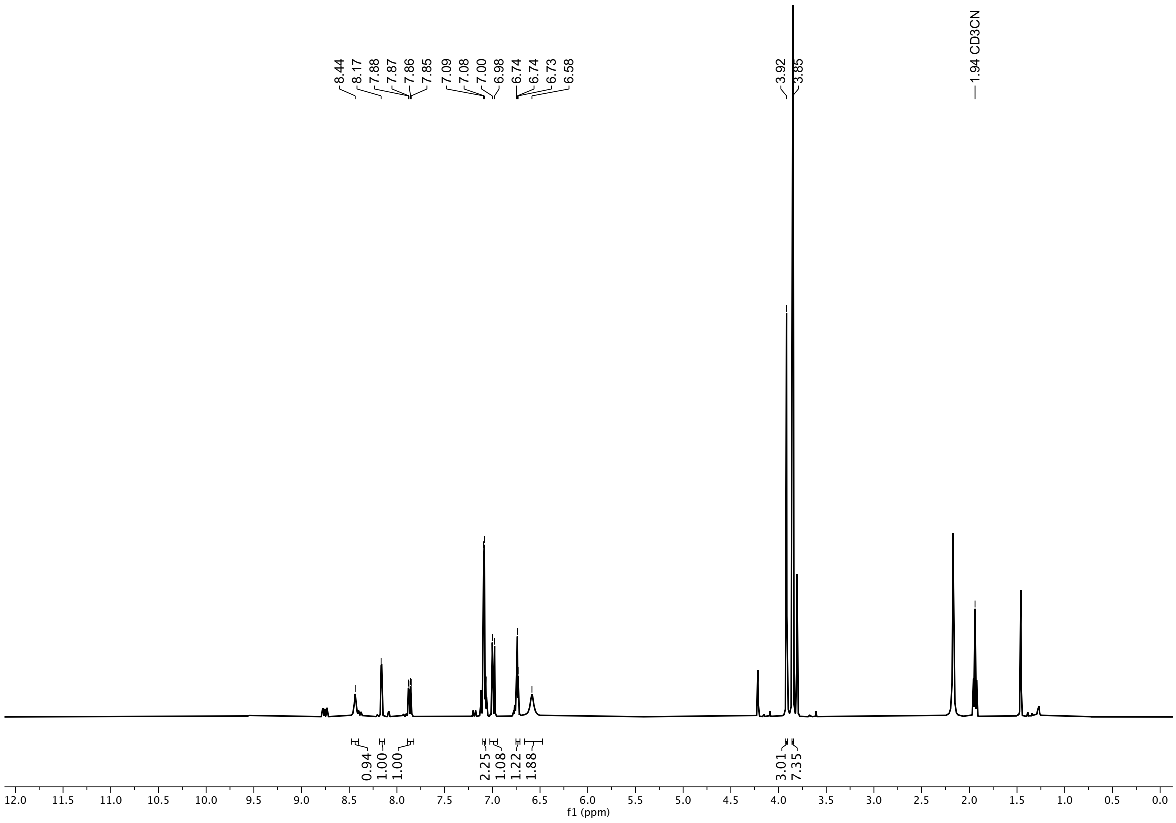

Figure S17. Solution ^{1}H NMR spectrum (CD_3CN, 298 K, 300 MHz) of **2b**.

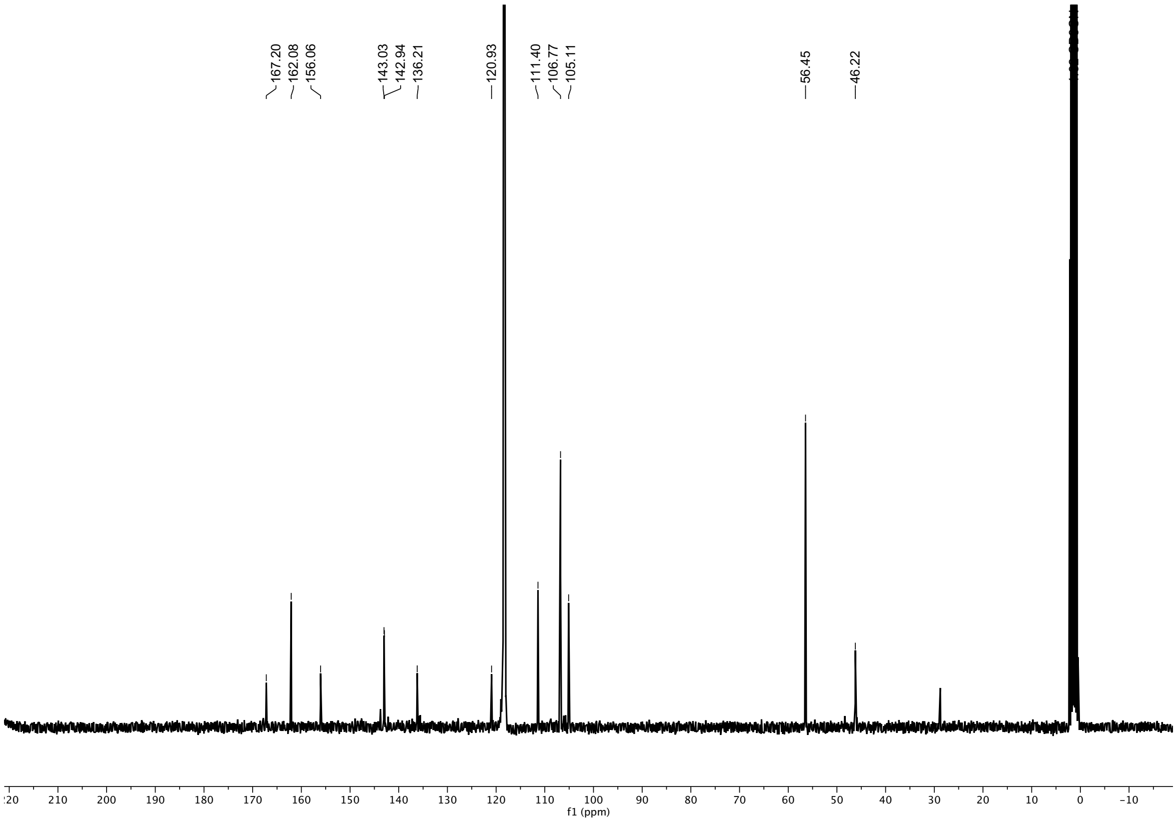

Figure S18. Solution $^{13}C\{^{1}H\}$ NMR spectrum (CD_3CN, 298 K, 75 MHz) of **2b**.

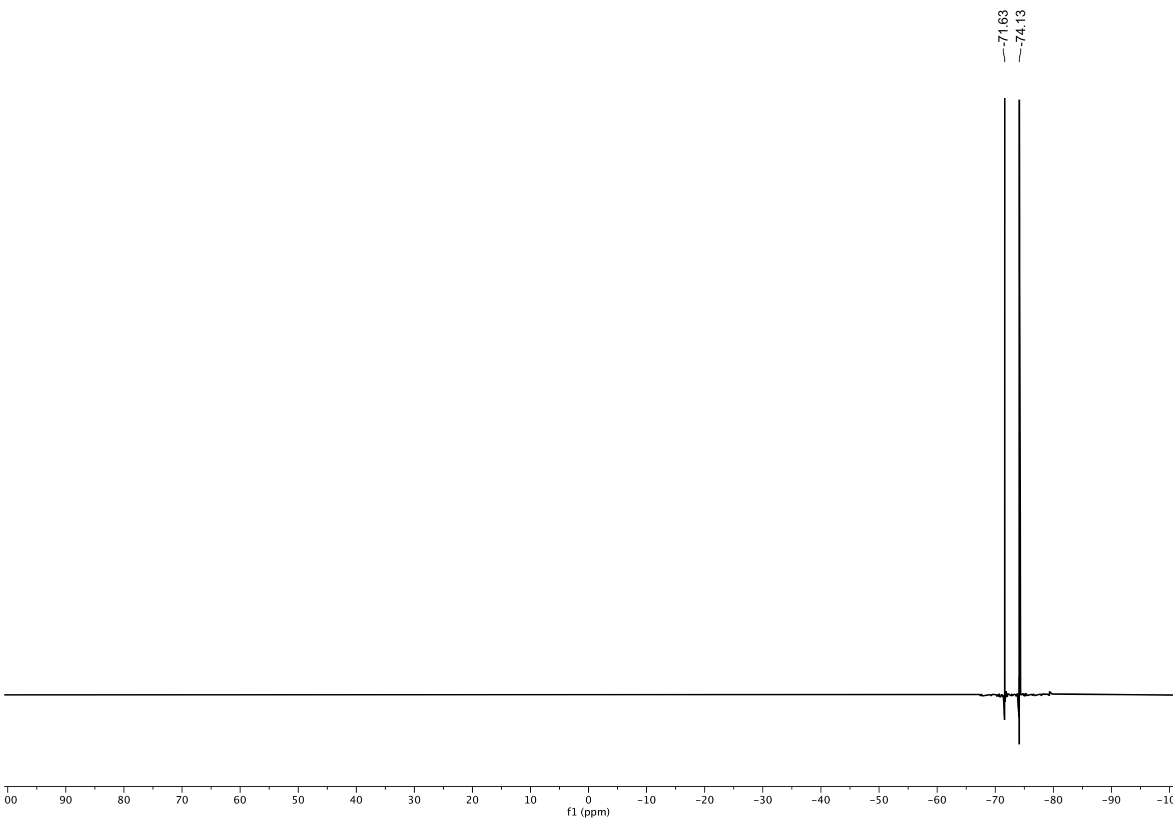

Figure S19. Solution ^{19}F NMR spectrum (CD_3CN, 298 K, 282 MHz) of **2b**.

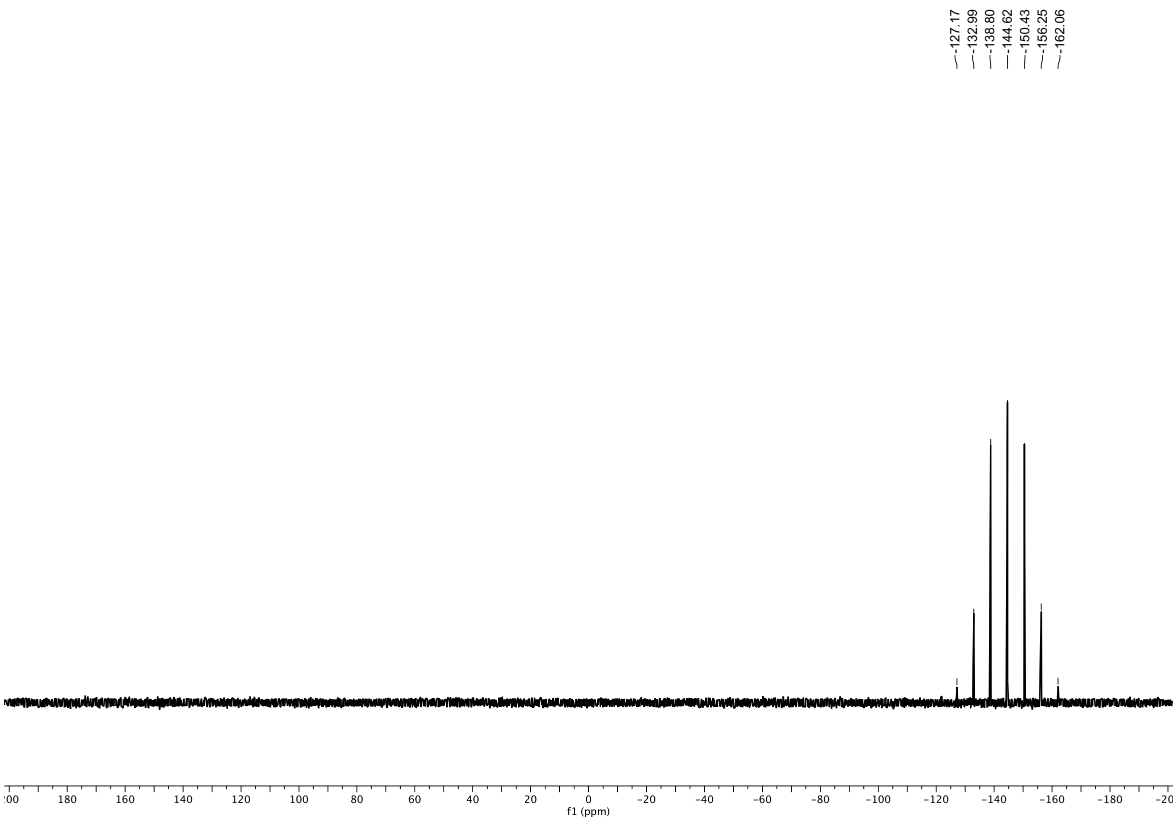

Figure S20. Solution ^{31}P NMR spectrum (CD_3CN, 298 K, 121 MHz) of **2b**.

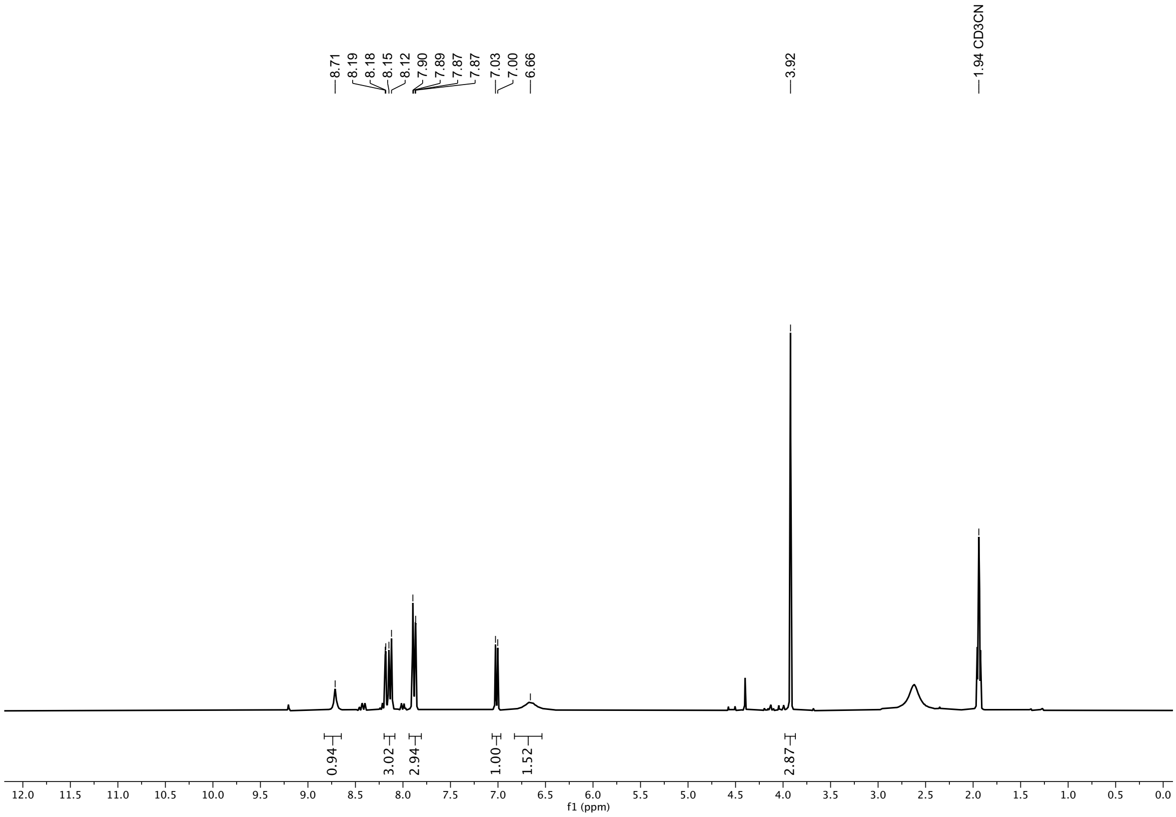

Figure S21. Solution ^{1}H NMR spectrum (CD_3CN, 298 K, 300 MHz) of **2c**.

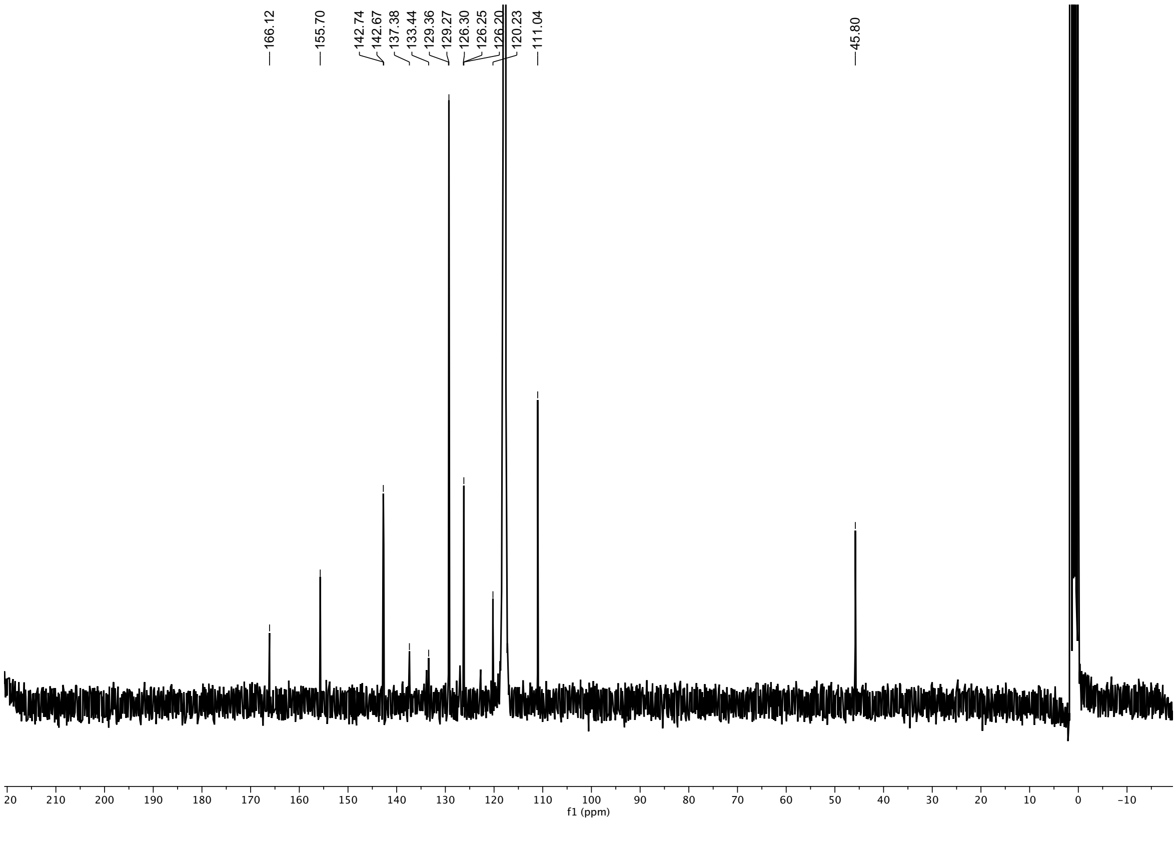

Figure S22. Solution ^{13}C{^{1}H} NMR spectrum (CD_3CN, 298 K, 75 MHz) of **2c**.

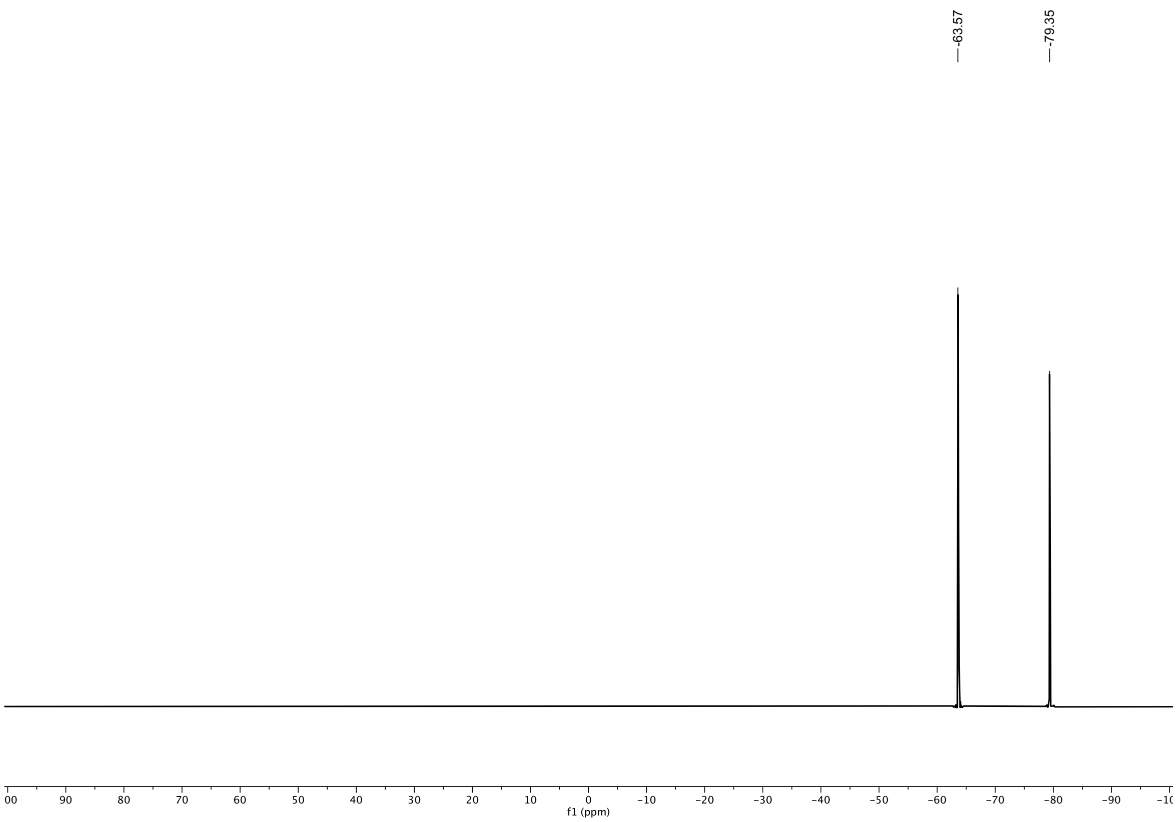

Figure S23. Solution ^{19}F NMR spectrum (CD_3CN, 298 K, 282 MHz) of **2c**.

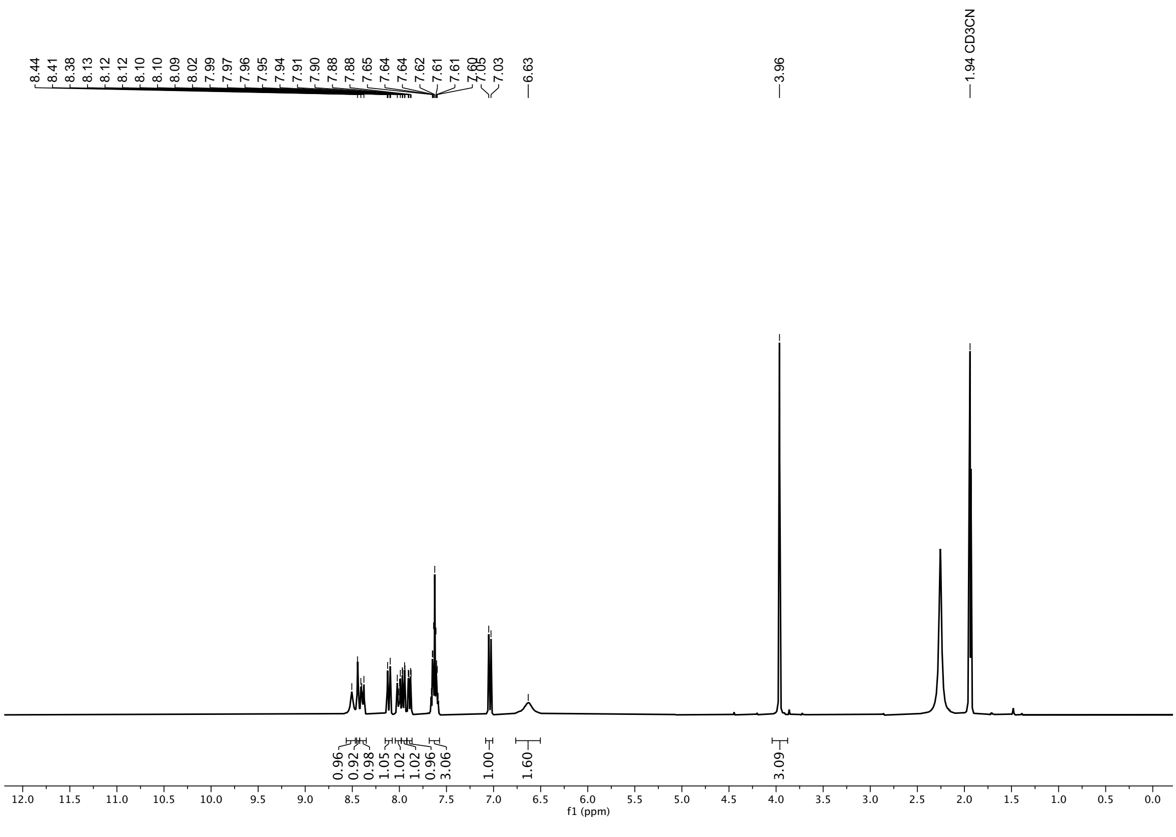

Figure S24. Solution ^{1}H NMR spectrum (CD_3CN, 298 K, 300 MHz) of **2d**.

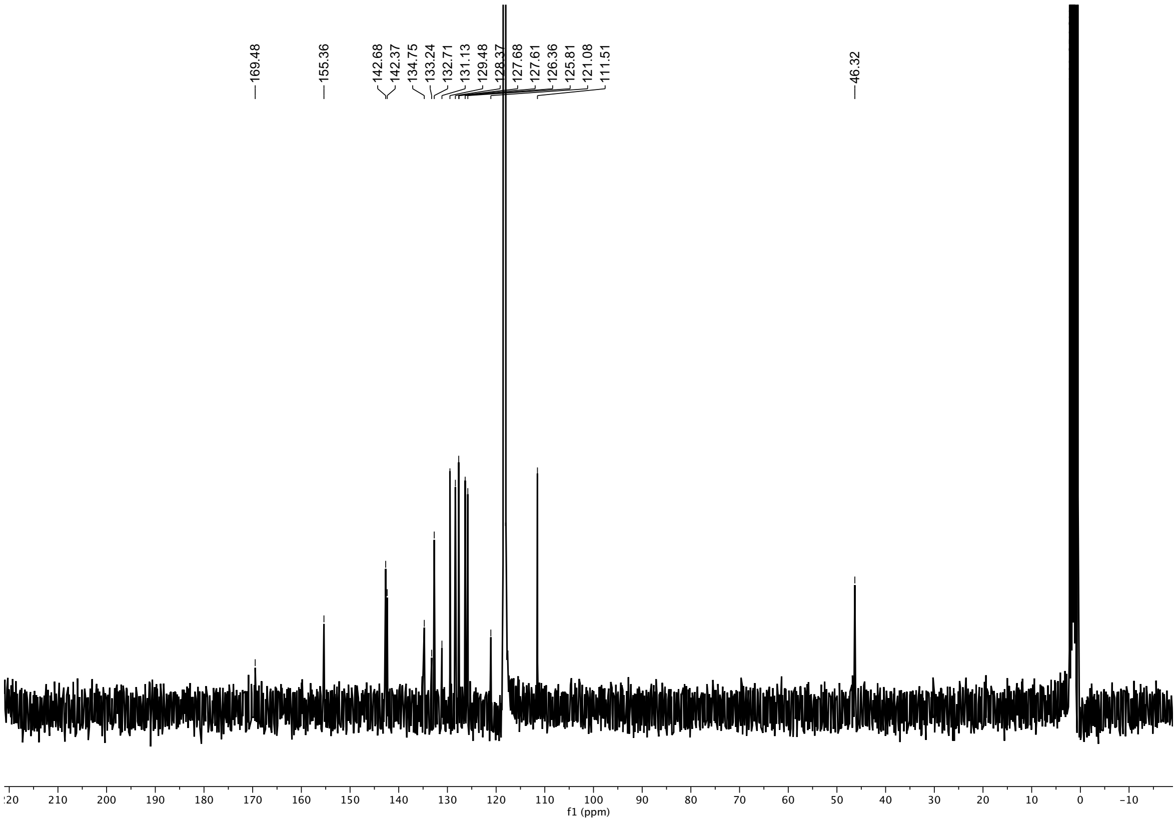

Figure S25. Solution $^{13}C\{^{1}H\}$ NMR spectrum (CD_3CN, 298 K, 75 MHz) of **2d**.

Figure S26. Solution ^{19}F NMR spectrum (CD_3CN, 298 K, 282 MHz) of **2d**.

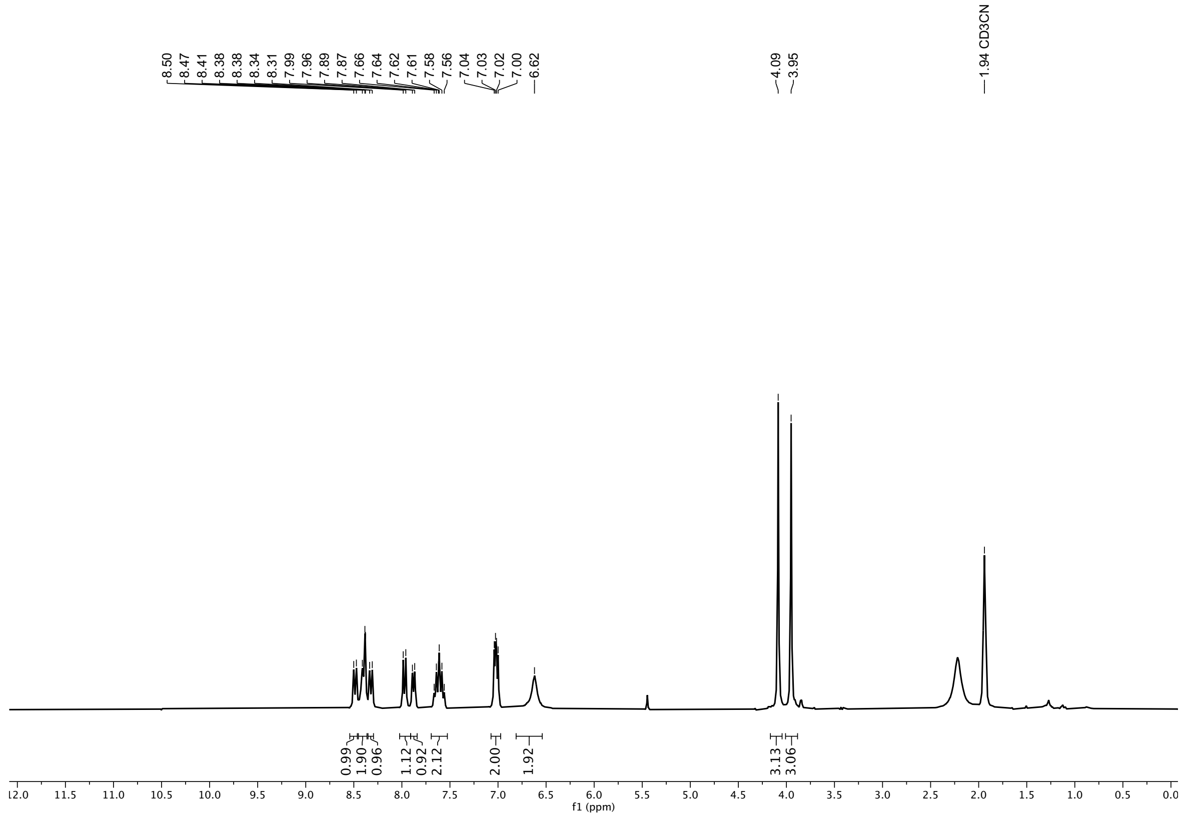

Figure S27. Solution ^{1}H NMR spectrum (CD_3CN, 298 K, 300 MHz) of **2e**.

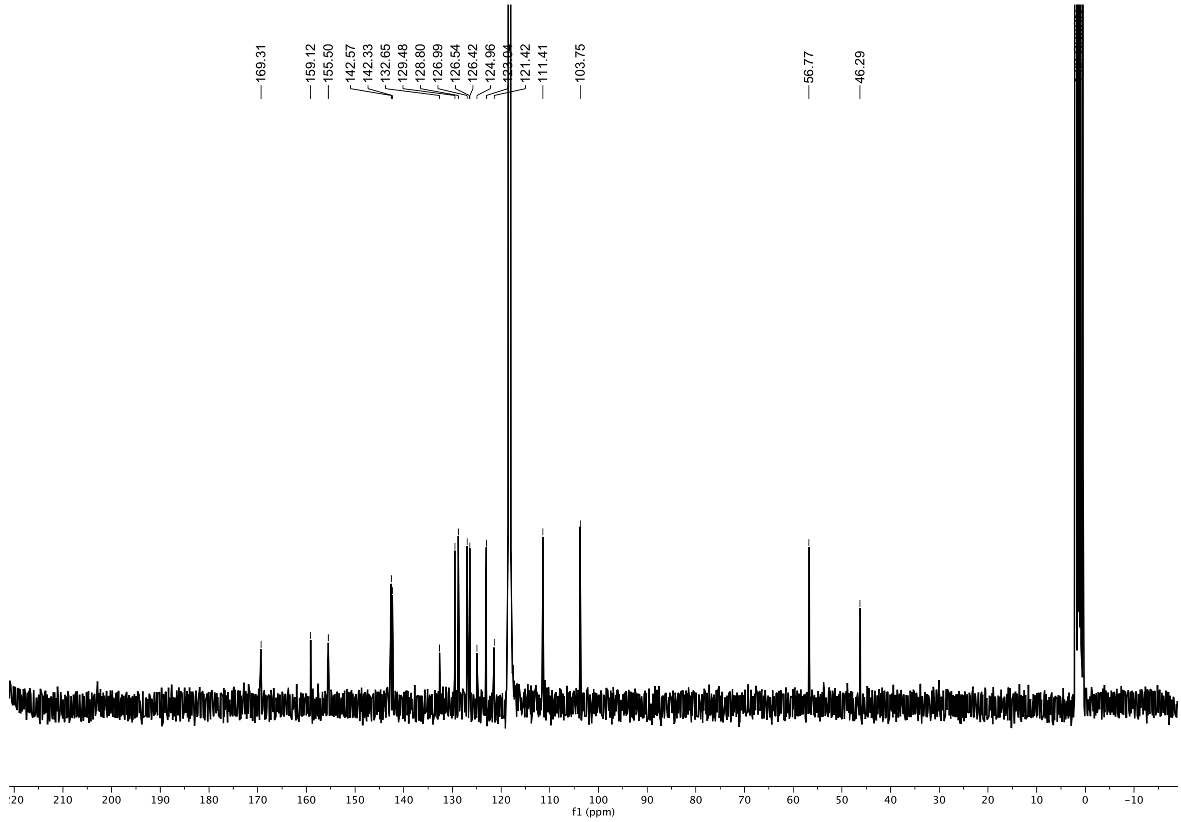

Figure S28. Solution $^{13}C\{^{1}H\}$ NMR spectrum (CD_3CN, 298 K, 75 MHz) of **2e**.

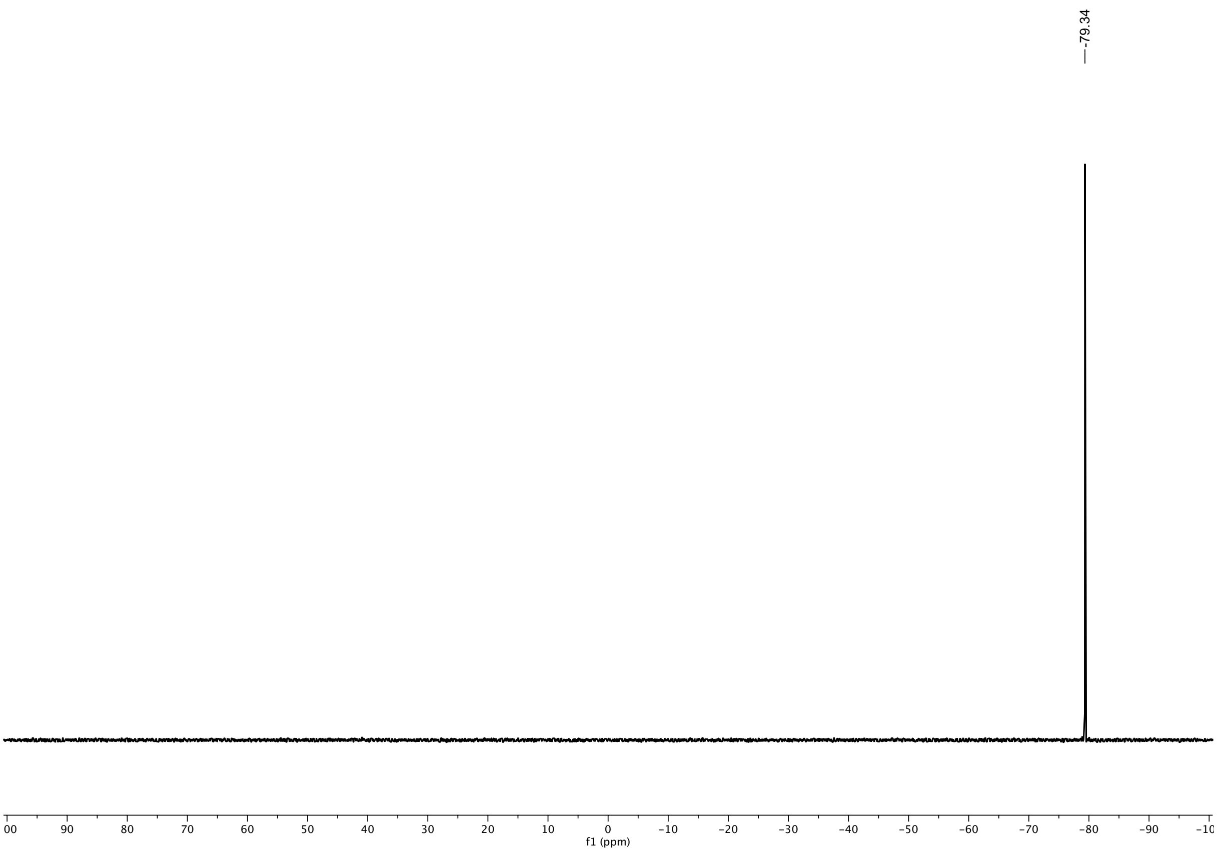

Figure S29. Solution ^{19}F NMR spectrum (CD_3CN, 298 K, 282 MHz) of **2e**.

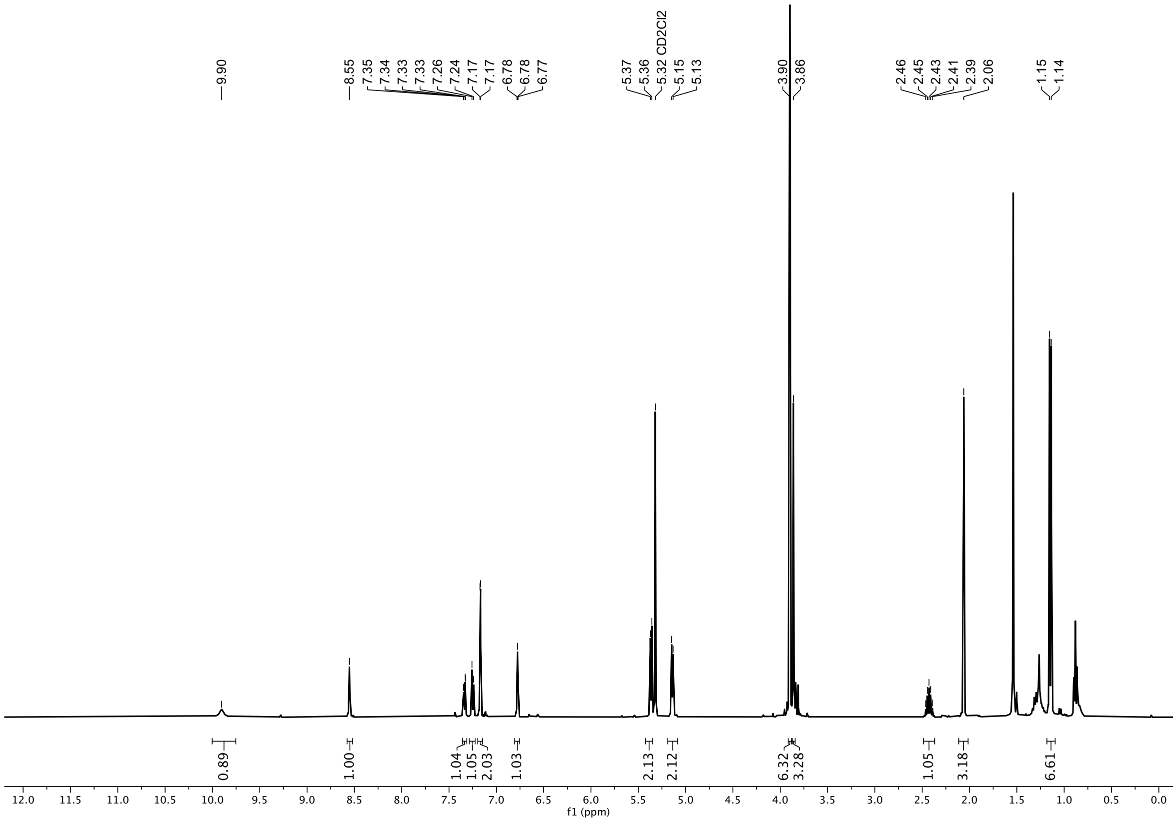

Figure S30. Solution ^{1}H NMR spectrum (CD_2Cl_2, 298 K, 300 MHz) of **3b**.

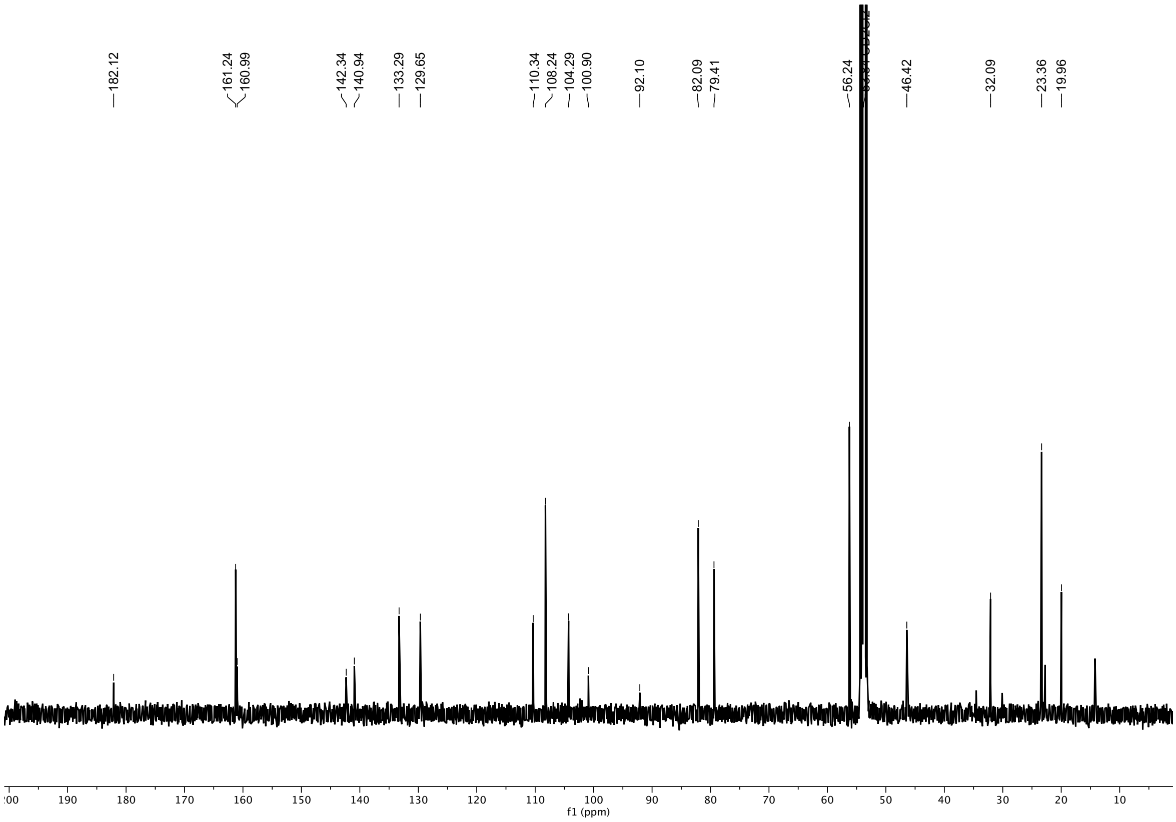

Figure S31. Solution $^{13}C\{^{1}H\}$ NMR spectrum (CD_2Cl_2, 298 K, 75 MHz) of **3b**.

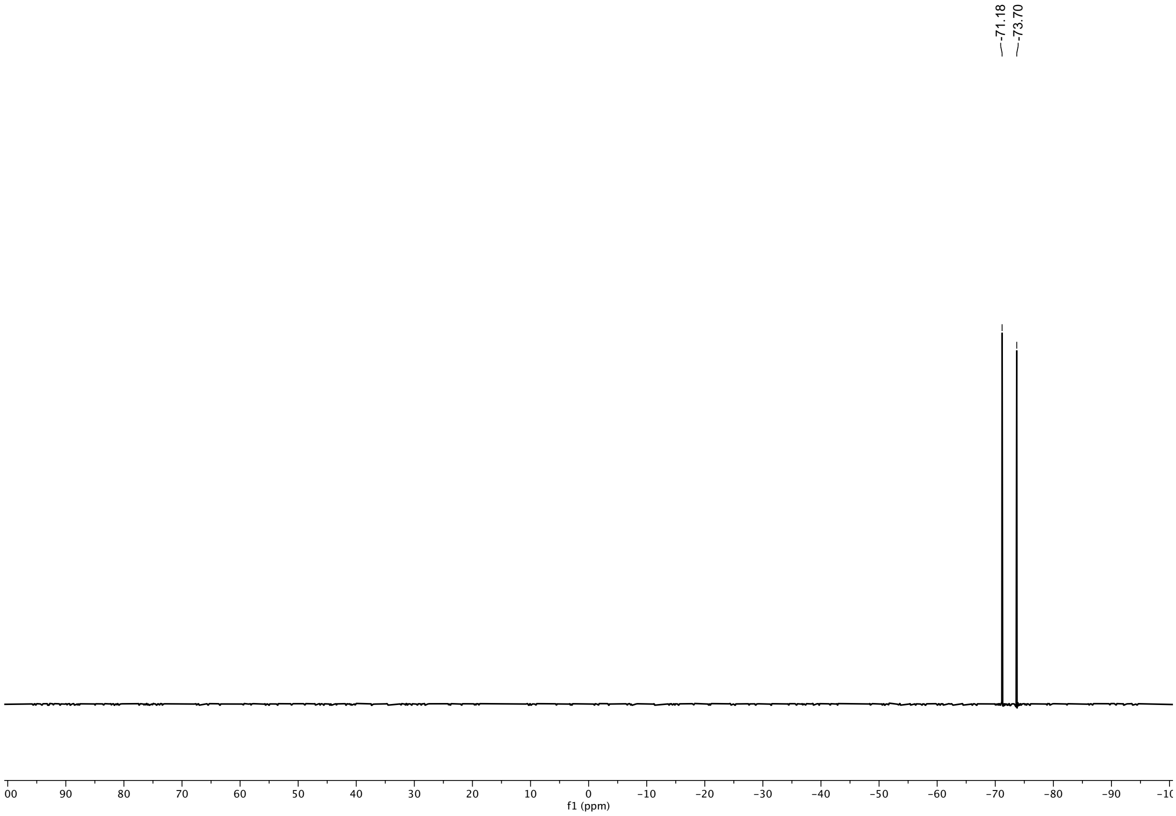

Figure S32. Solution ^{19}F NMR spectrum (CD_2Cl_2, 298 K, 282 MHz) of **3b**.

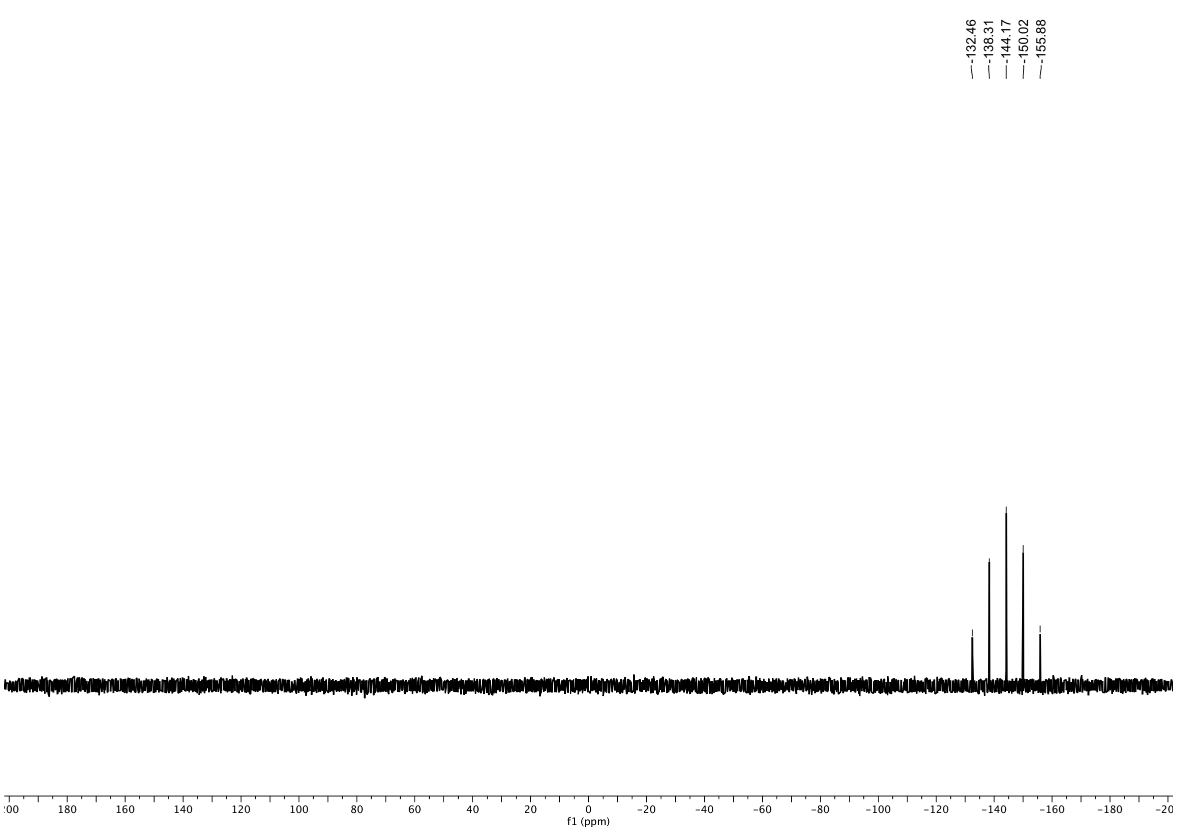

Figure S33. Solution ^{31}P NMR spectrum (CD_2Cl_2, 298 K, 121 MHz) of **3b**.

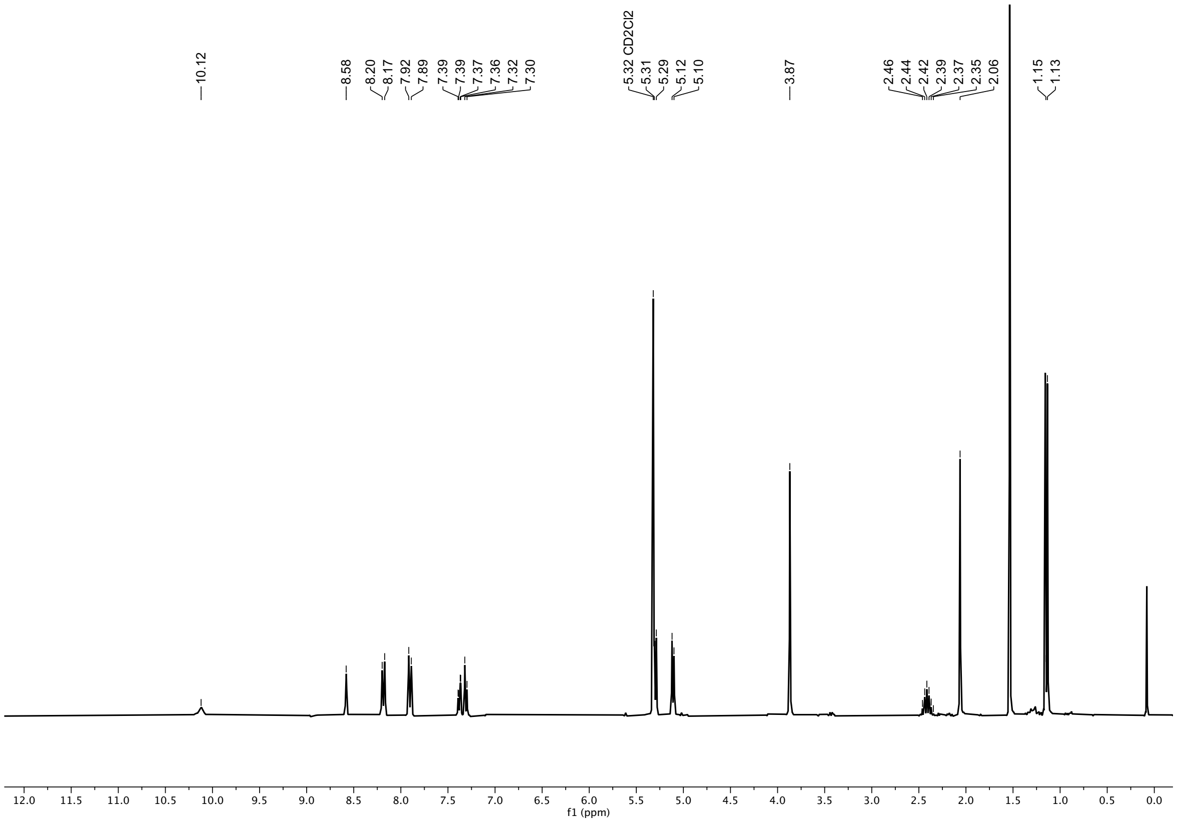

Figure S34. Solution ^{1}H NMR spectrum (CD_2Cl_2, 298 K, 300 MHz) of **3c**.

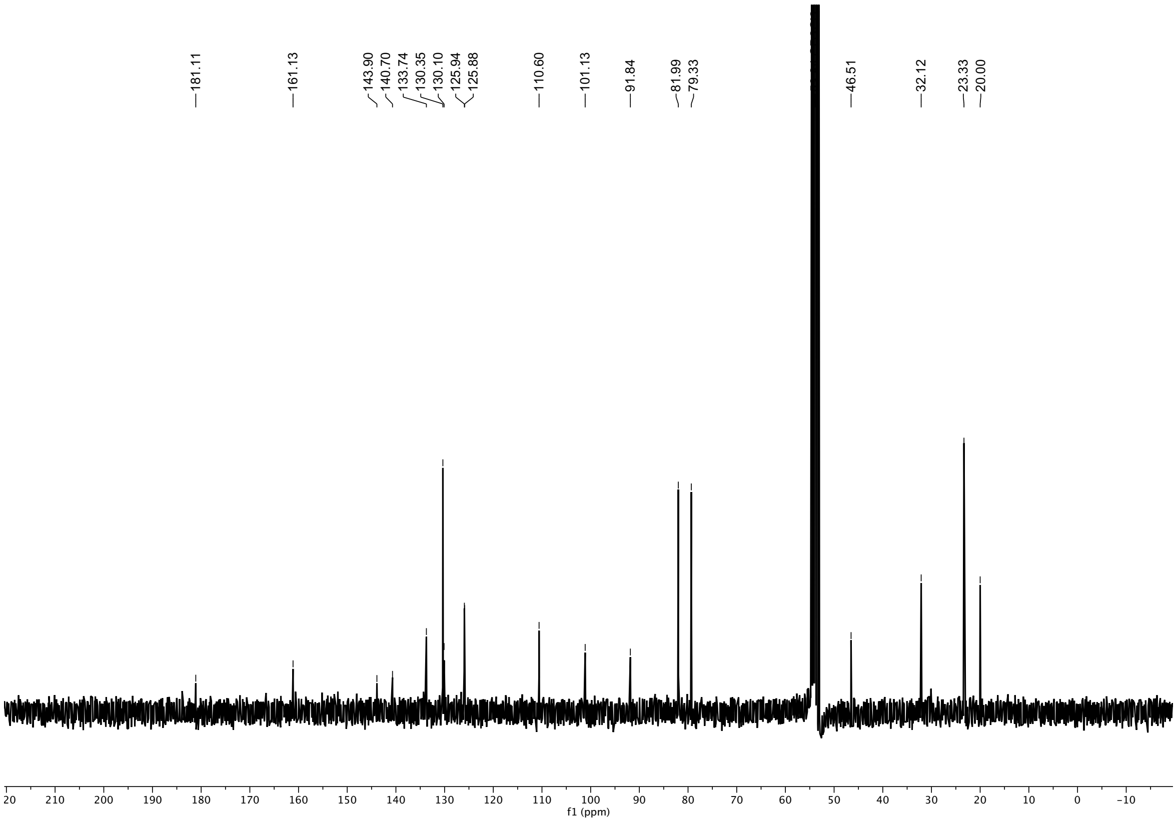

Figure S35. Solution $^{13}C\{^{1}H\}$ NMR spectrum (CD_3CN, 298 K, 75 MHz) of **3c**.

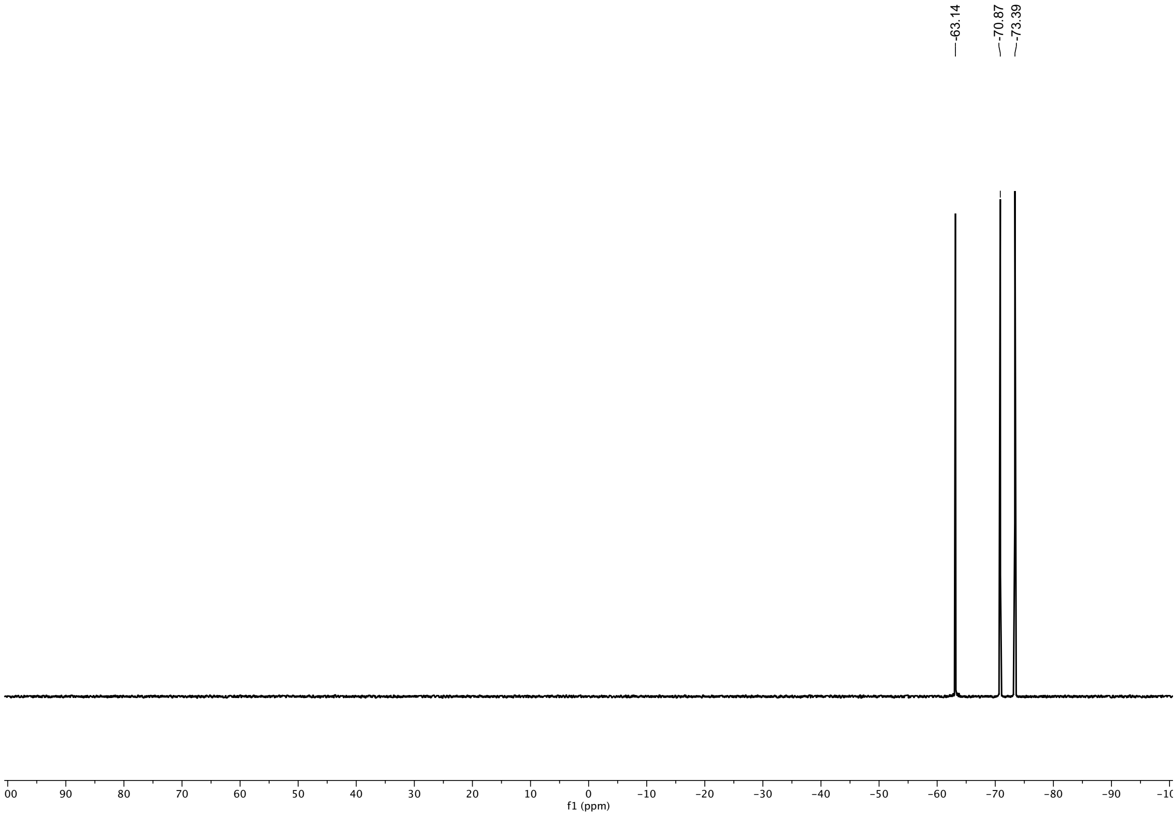

Figure S36. Solution ^{19}F NMR spectrum (CD_2Cl_2, 298 K, 282 MHz) of **3c**.

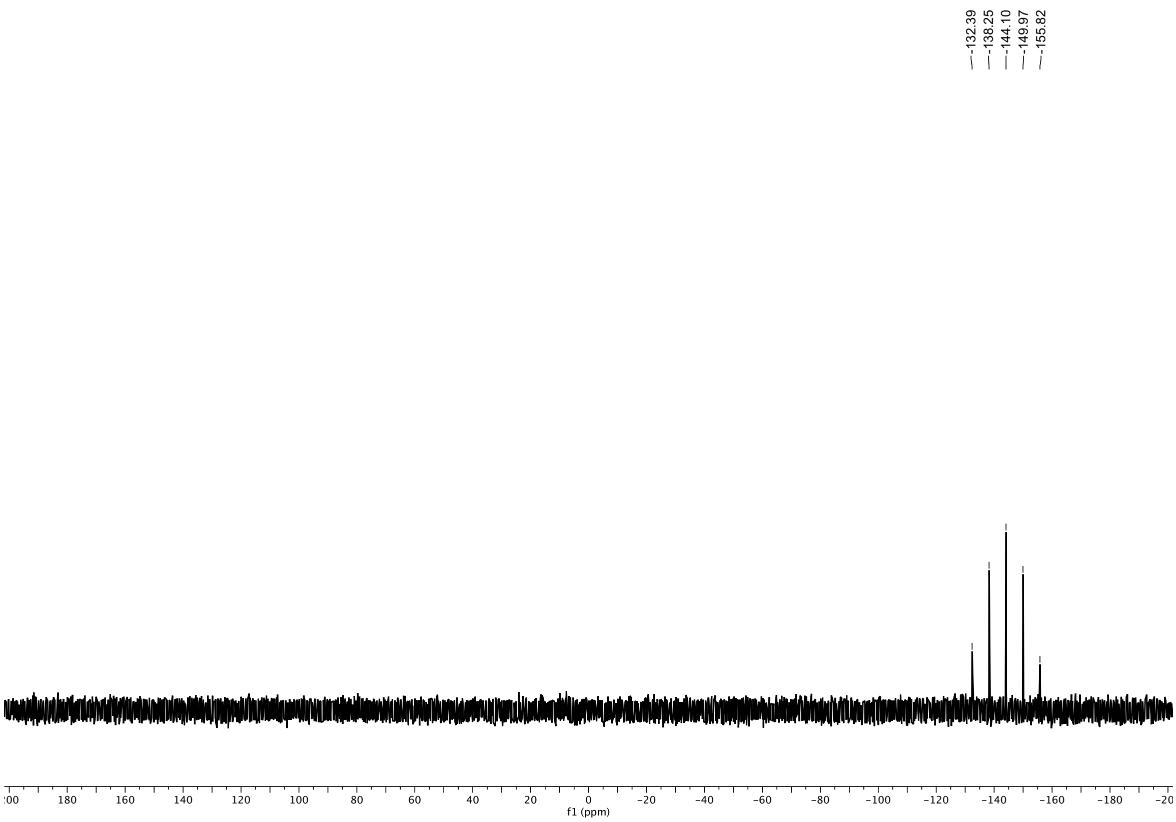

Figure S37. Solution ^{31}P NMR spectrum (CD_2Cl_2, 298 K, 121 MHz) of **3c**.

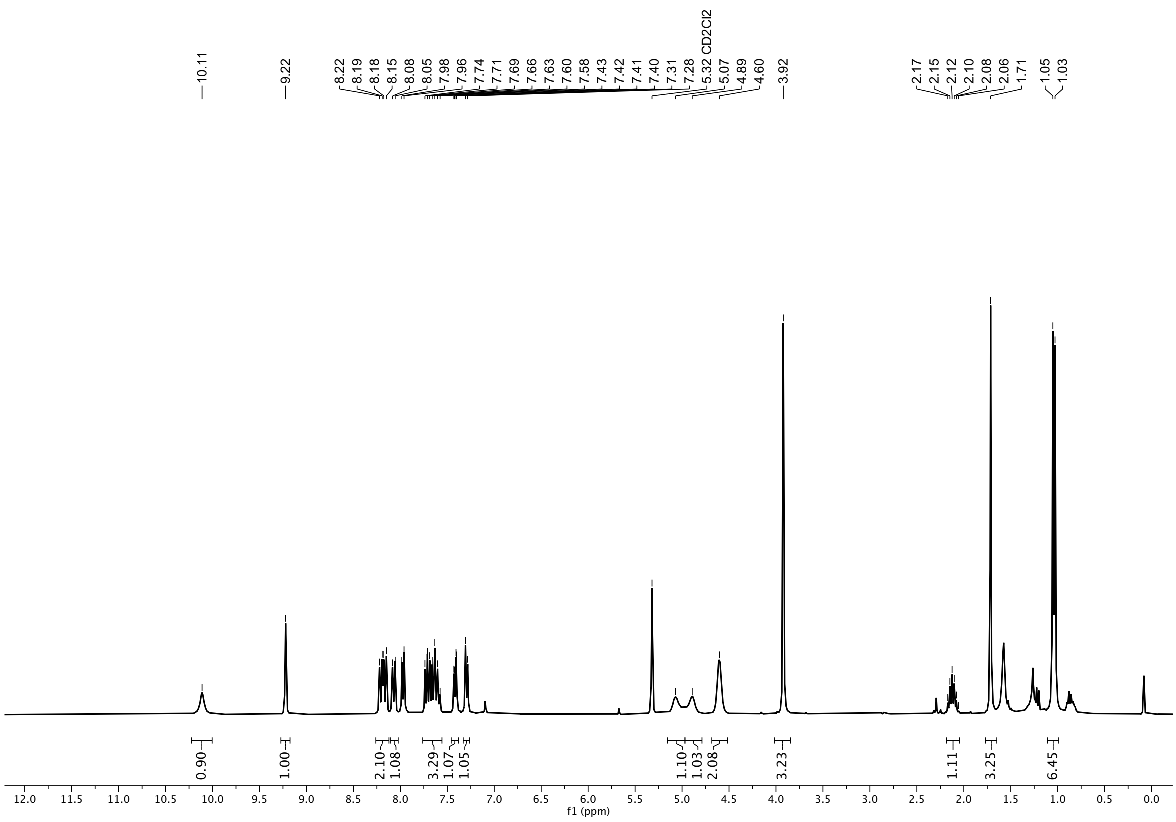

Figure S38. Solution ^{1}H NMR spectrum (CD_2Cl_2, 298 K, 300 MHz) of **3d**.

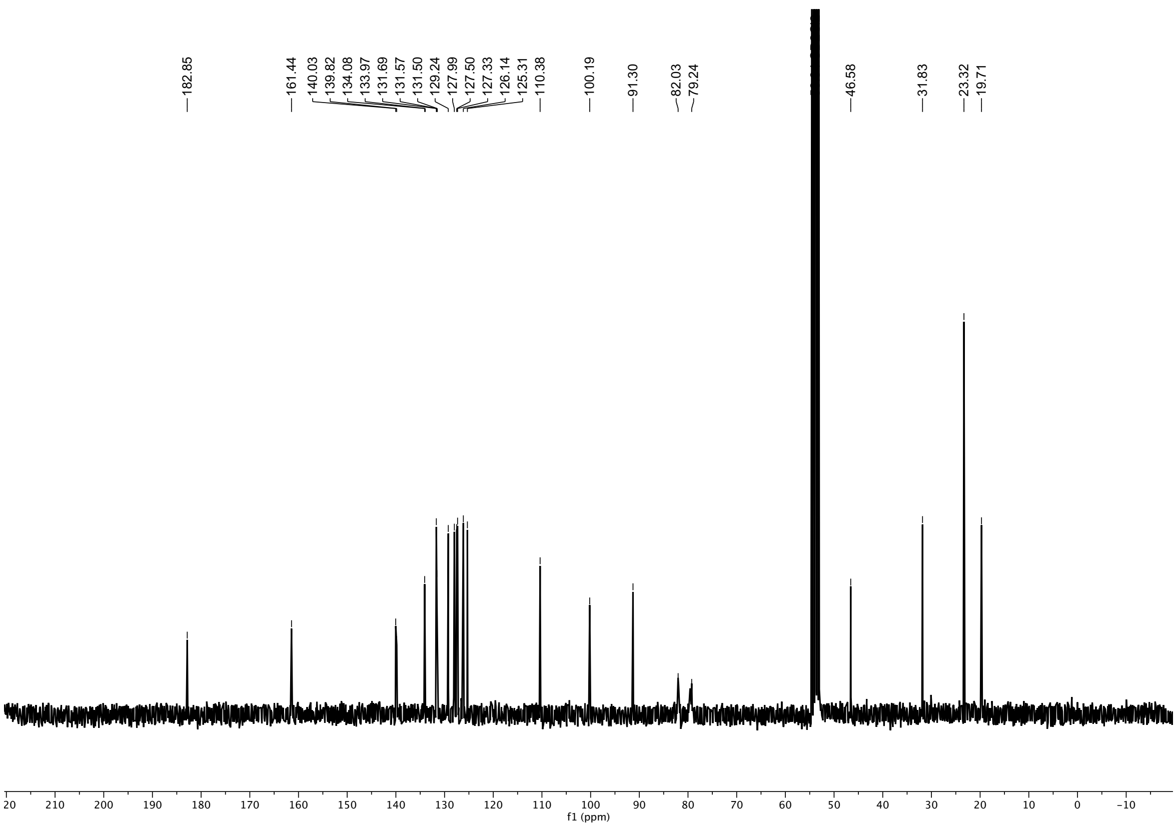

Figure S39. Solution $^{13}C\{^{1}H\}$ NMR spectrum (CD_3CN, 298 K, 75 MHz) of **3d**.

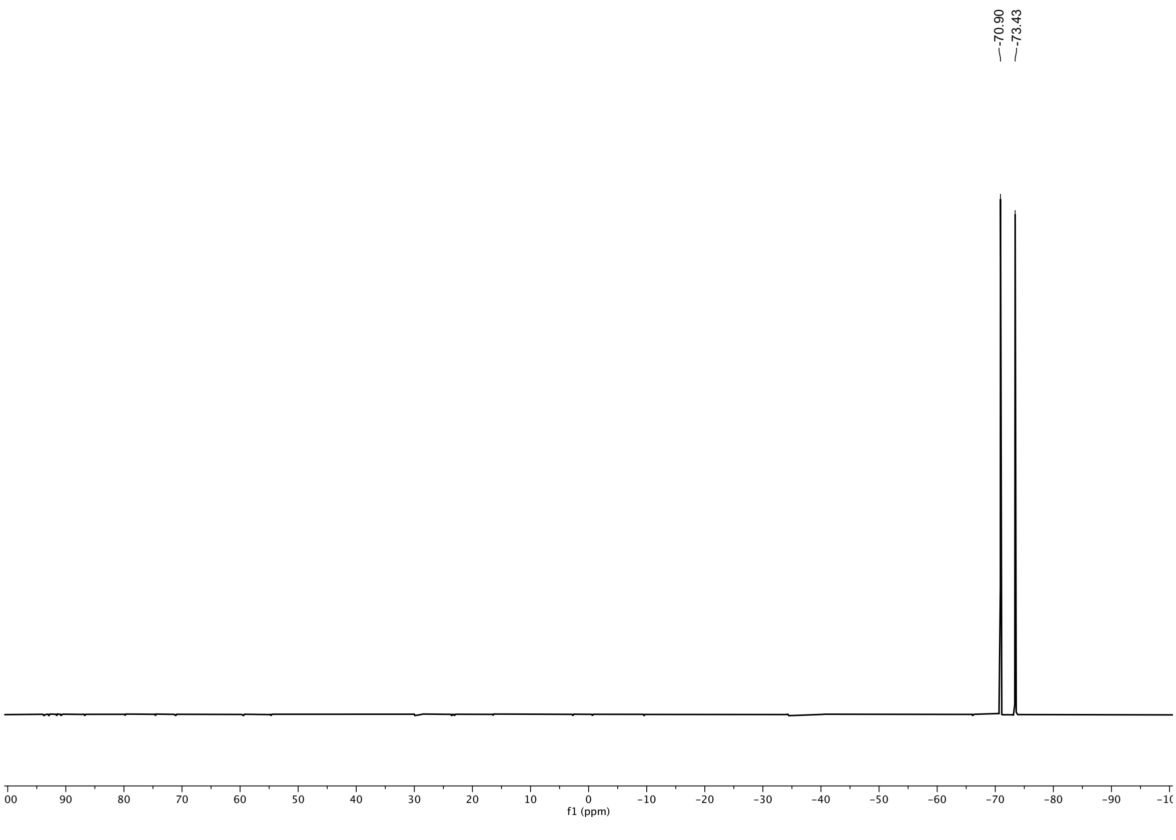

Figure S40. Solution ^{19}F NMR spectrum (CD_2Cl_2, 298 K, 282 MHz) of **3d**.

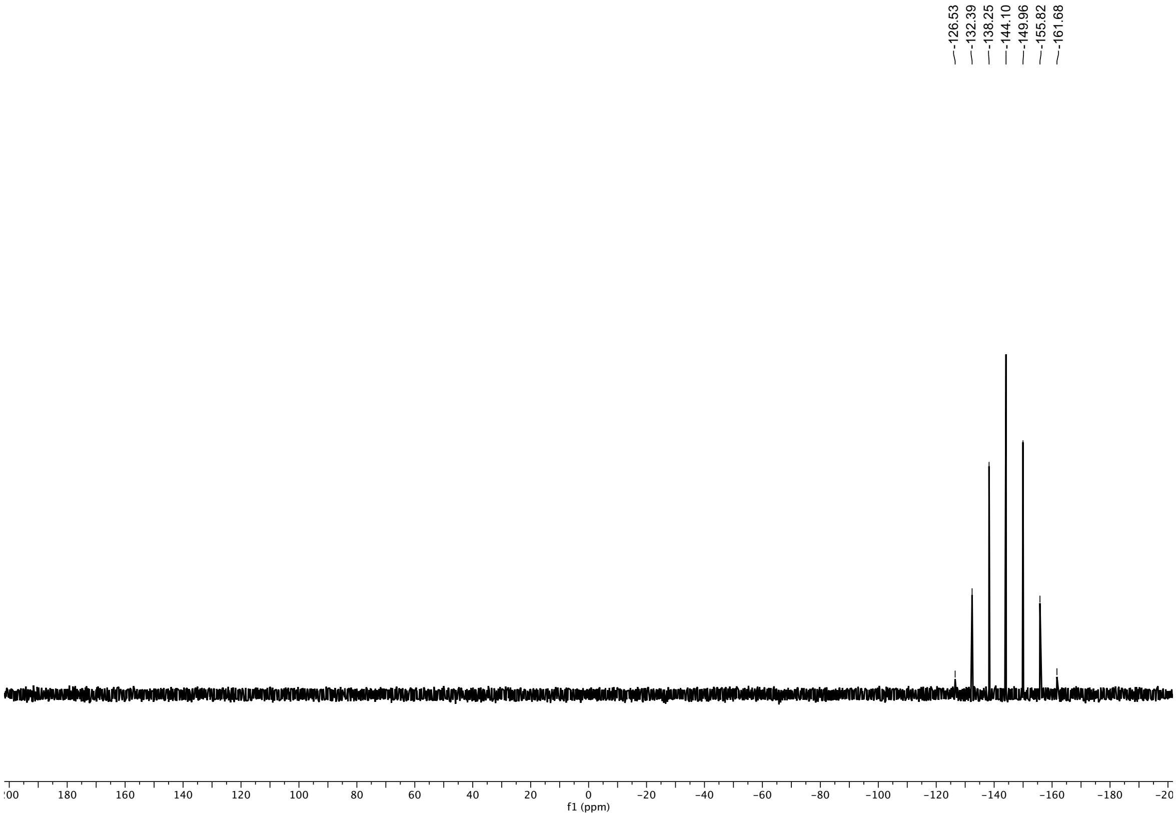

Figure S41. Solution ^{31}P NMR spectrum (CD_2Cl_2, 298 K, 121 MHz) of **3d**.

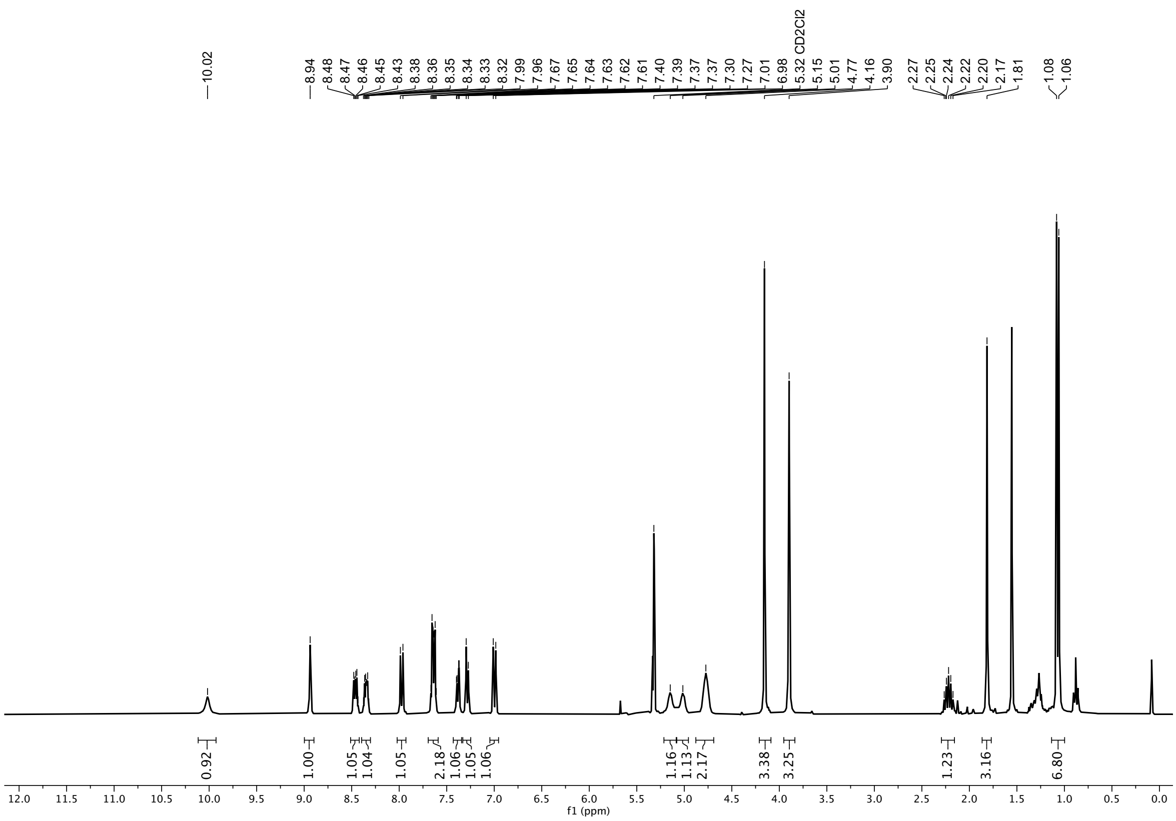

Figure S42. Solution ^{1}H NMR spectrum (CD_2Cl_2, 298 K, 300 MHz) of **3e**.

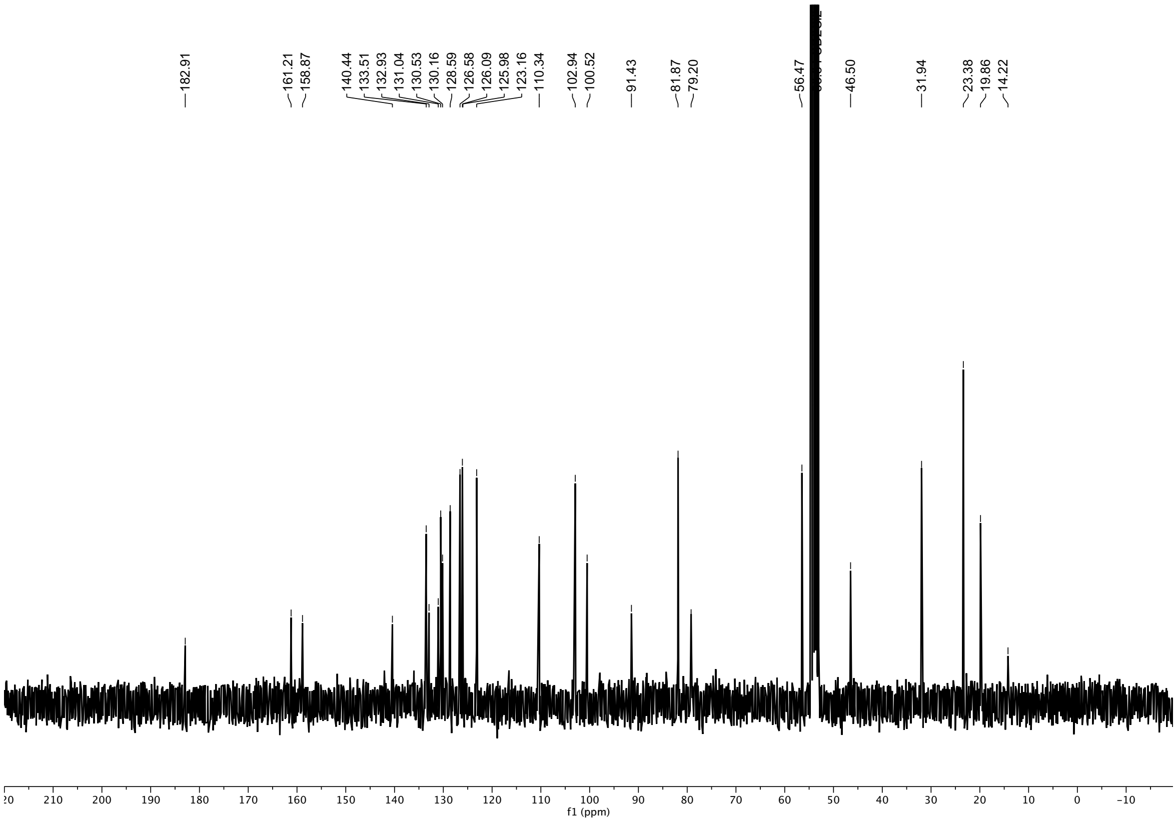

Figure S43. Solution $^{13}C\{^{1}H\}$ NMR spectrum (CD_3CN, 298 K, 75 MHz) of **3e**.

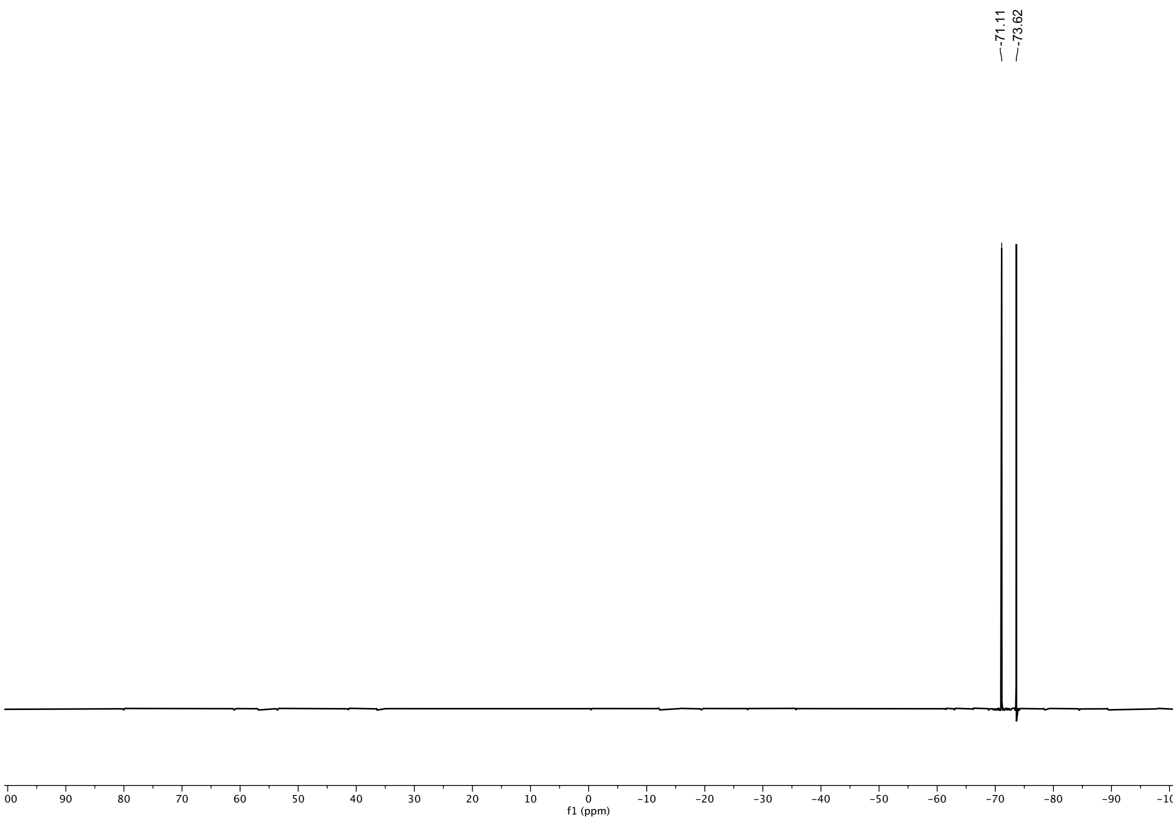

Figure S44. Solution ^{19}F NMR spectrum (CD_2Cl_2, 298 K, 282 MHz) of **3e**.

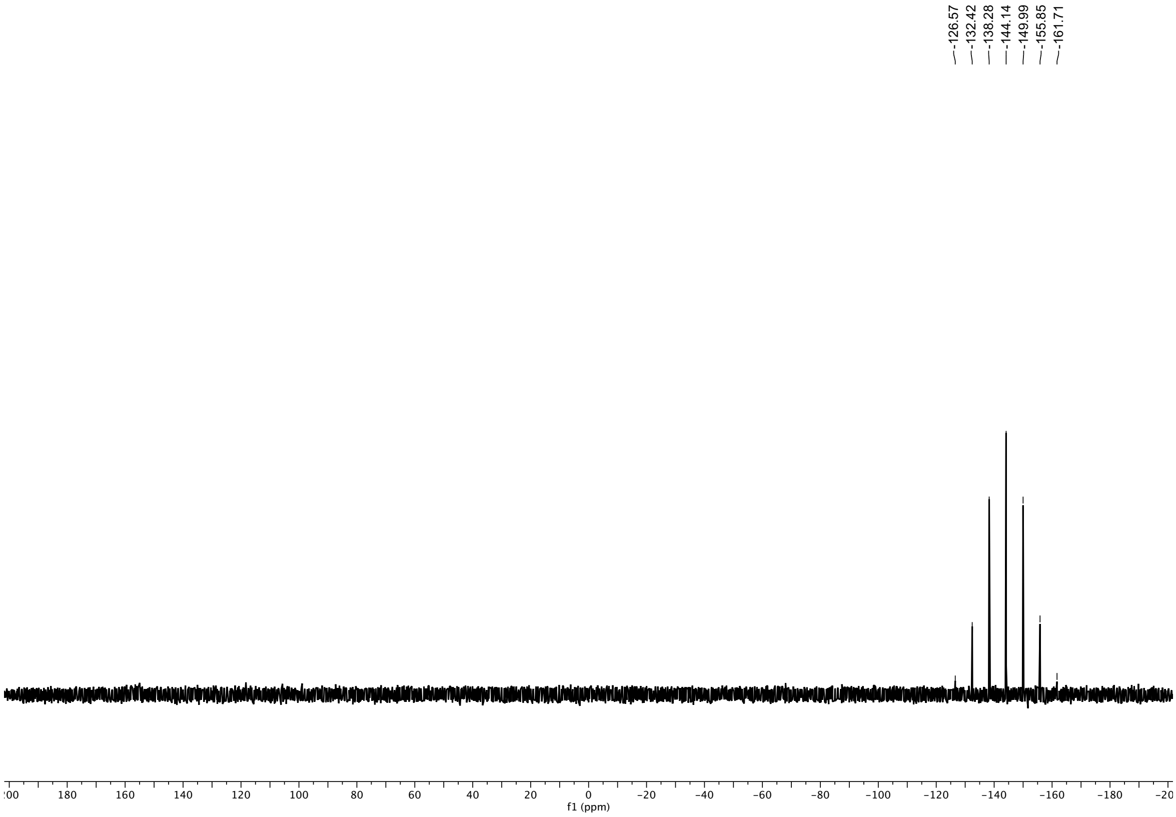

Figure S45. Solution ^{31}P NMR spectrum (CD_2Cl_2, 298 K, 121 MHz) of **3e**.

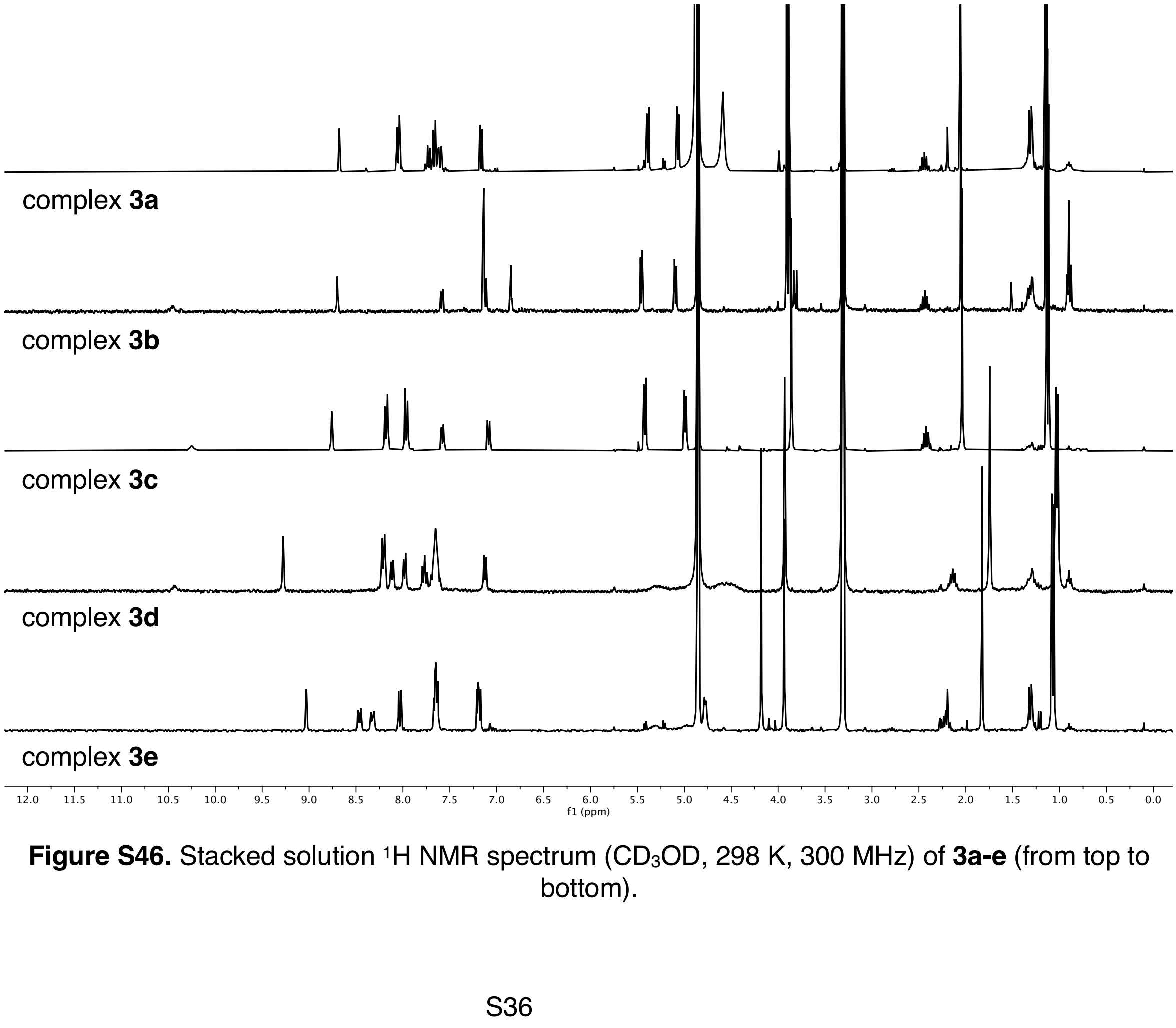

Figure S46. Stacked solution ^{1}H NMR spectrum (CD_3OD, 298 K, 300 MHz) of **3a-e** (from top to bottom).

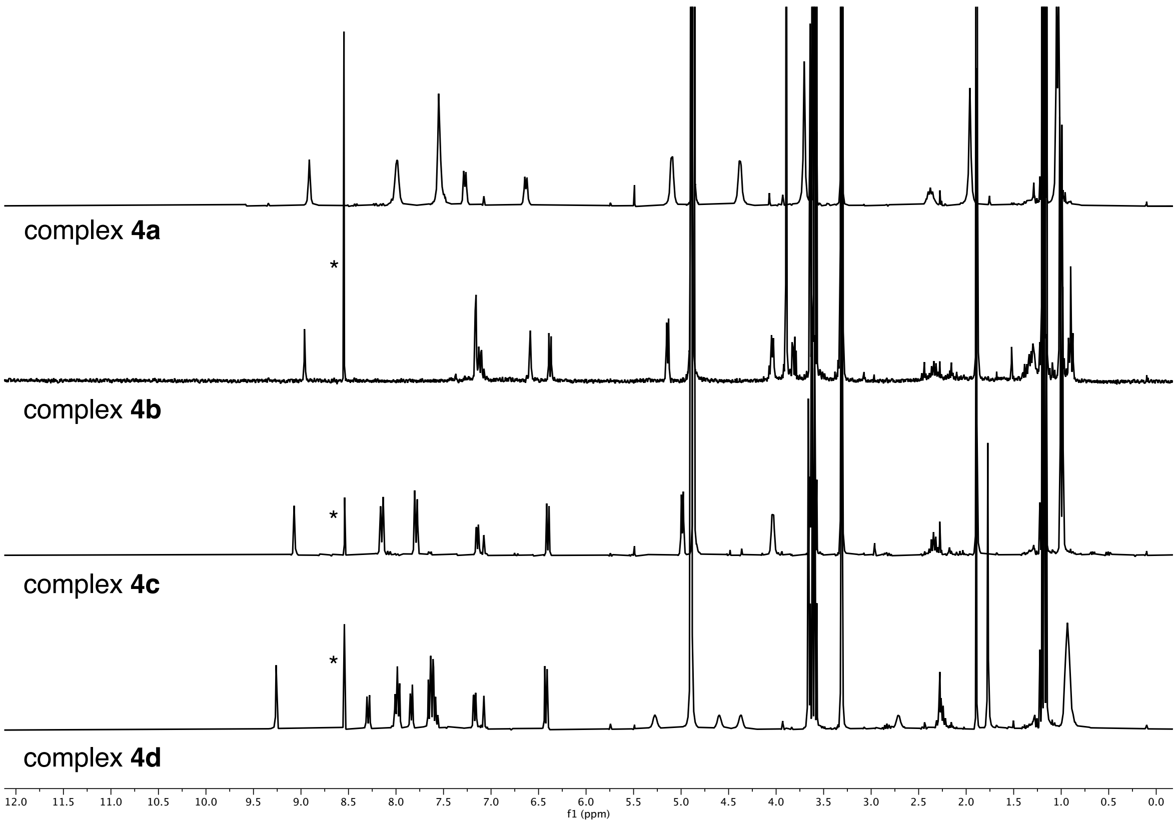

Figure S47. Stacked solution ^{1}H NMR spectrum (CD_3OD, 298 K, 300 MHz) of **4a-d** (from top to bottom); * represents NaOEt impurity.

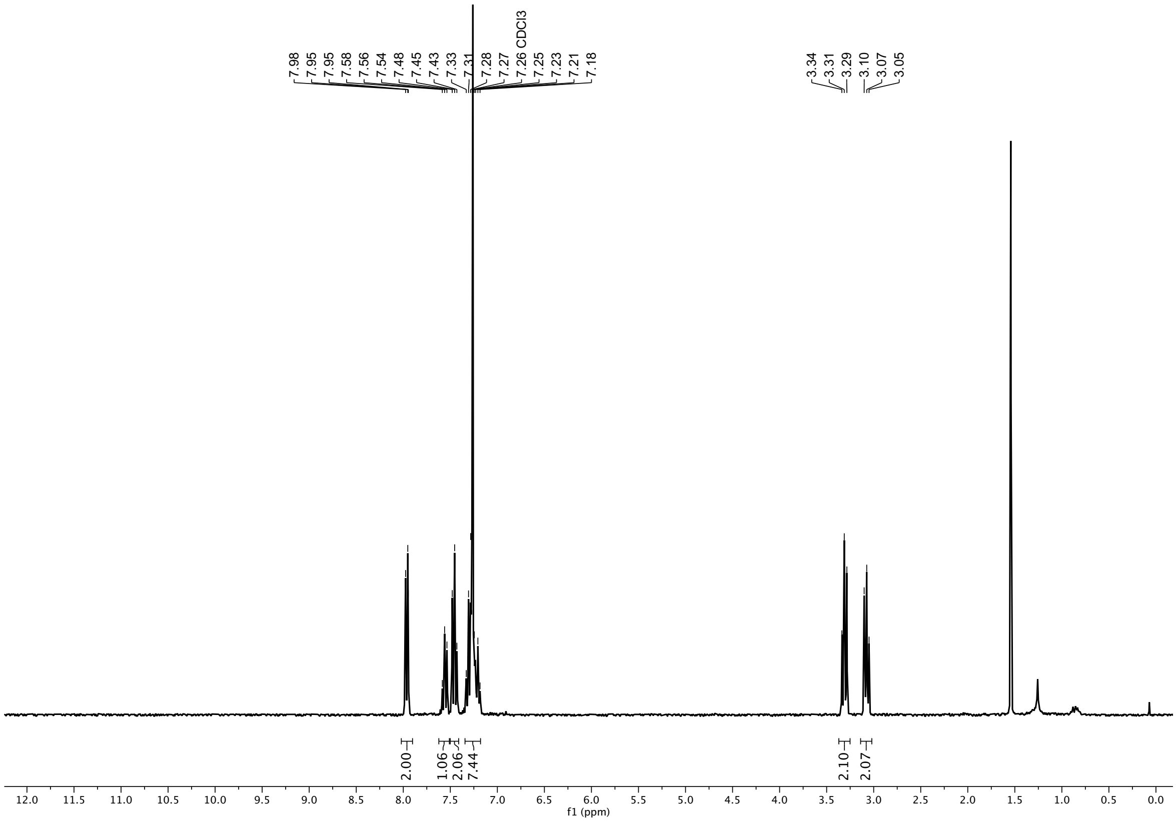

Figure S48. Solution ^{1}H NMR spectrum ($CDCl_3$, 298 K, 300 MHz) of **6a**.

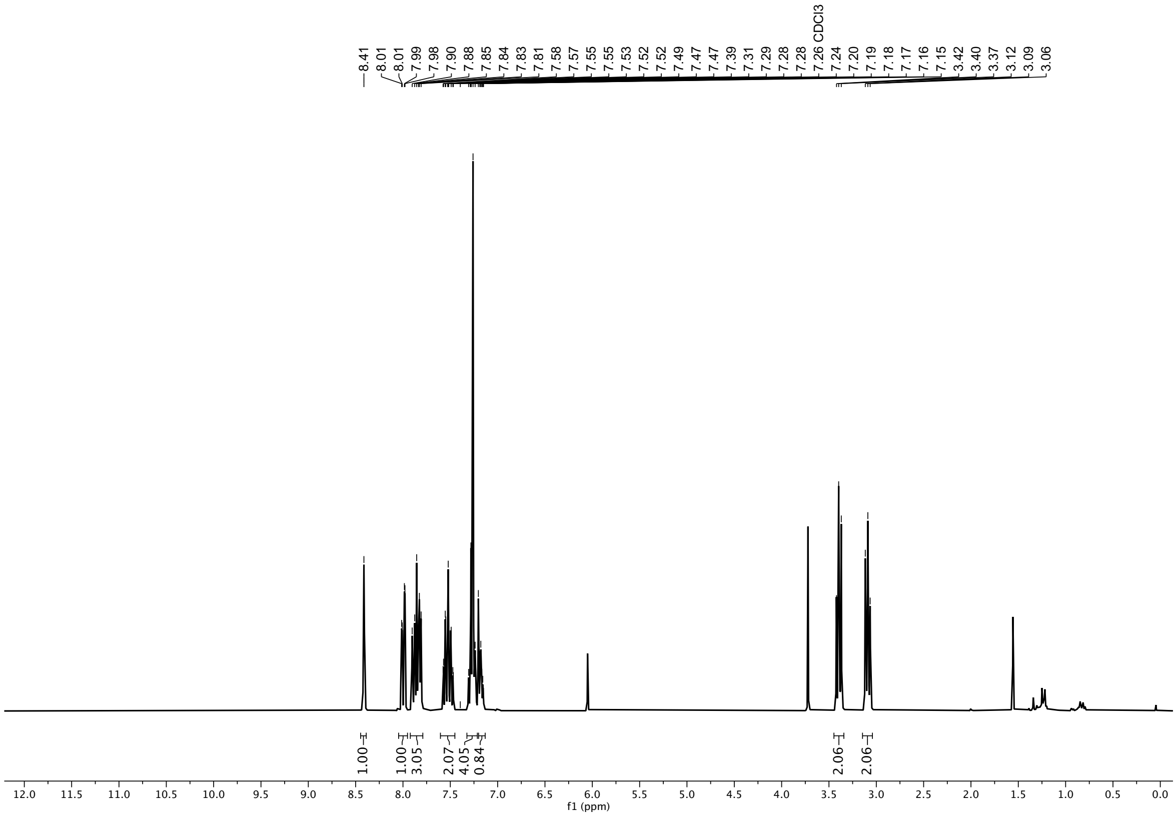

Figure S49. Solution ^{1}H NMR spectrum ($CDCl_3$, 298 K, 300 MHz) of **6b**.

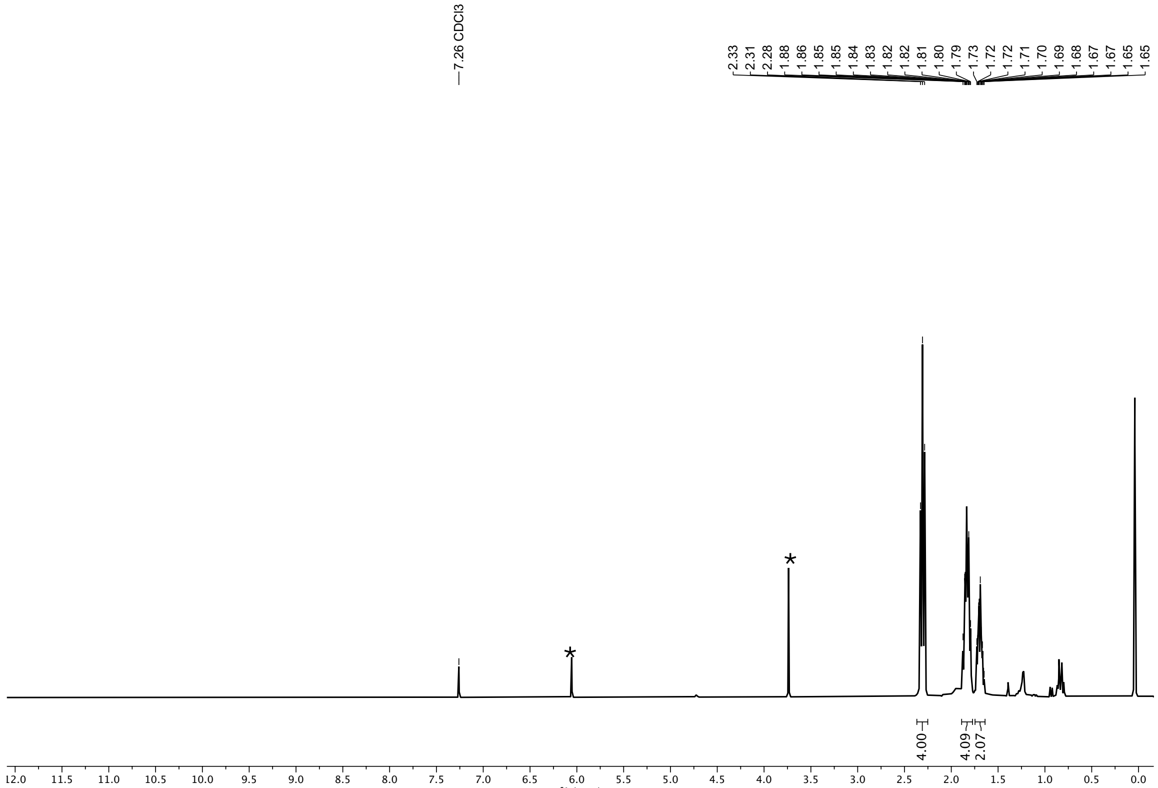

Figure S50. Solution ^{1}H NMR spectrum ($CDCl_3$, 298 K, 300 MHz) of **6e** (* = 1,3,5-trimethoxybenzene).

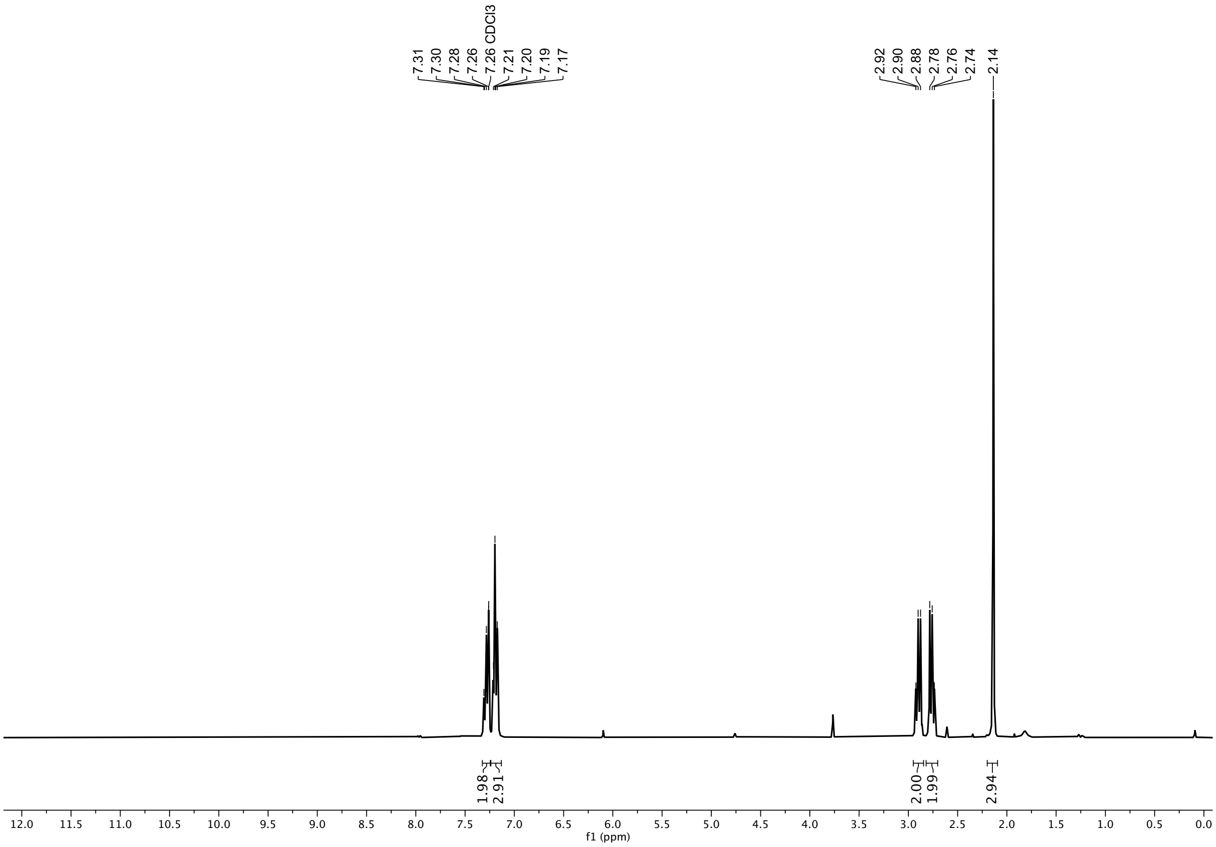

Figure S51. Solution ^{1}H NMR spectrum ($CDCl_3$, 298 K, 300 MHz) of **6d**.

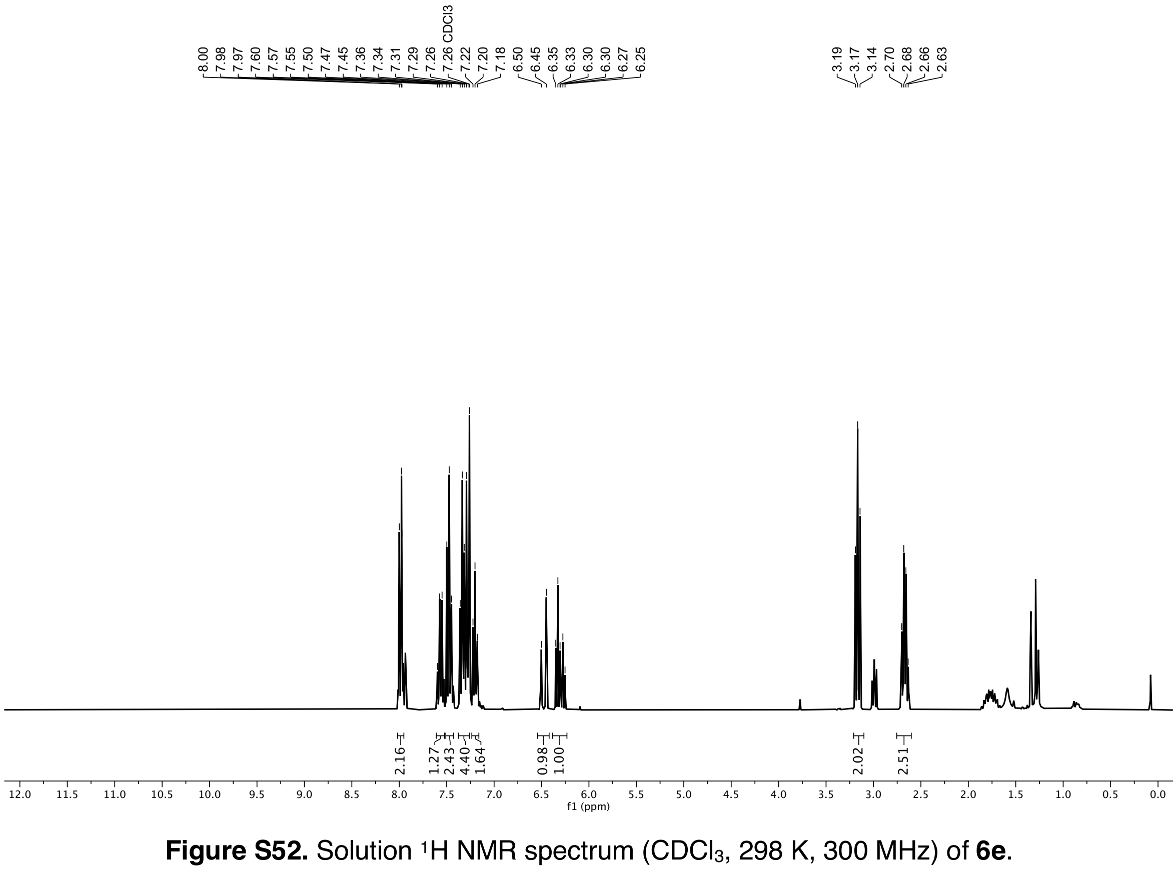

Figure S52. Solution ^{1}H NMR spectrum ($CDCl_3$, 298 K, 300 MHz) of **6e**.

S7 References

S1 J. C. Wong, G. Tang, X. Wu, C. Liang, Z. Zhang, L. Guo, Z. Peng, W. Zhang, X. Lin, Z. Wang, J. Mei, J. Chen, S. Pan, N. Zhang, Y. Liu, M. Zhou, L. Feng, W. Zhao, S. Li, C. Zhang, M. Zhang, Y. Rong, T.-G. Jin, X. Zhang, S. Ren, Y. Ji, R. Zhao, J. She, Y. Ren, C. Xu, D. Chen, J. Cai, S. Shan, D. Pan, Z. Ning, X. Lu, T. Chen, Y. He and L. Chen, *J. Med. Chem.,* 2012, **55**, 8903–8925.

S2 A. Beaufils, P. Melle, N. Lentz and M. Albrecht, *Inorg. Chem.,* 2024, **63**, 2072–2081.

S3 Y. Zuo, X. He, Q. Tang, W. Hu, T. Zhou, W. Hu and Y. Shang, *Adv. Synth. Catal.,* 2021, **363**, 2117–2123.

S4 G. Zhang, J. Wu, H. Zeng, S. Zhang and Z. Yin, S. Zheng, *Org. Lett.,* 2017, **19**, 1080–1083.

S5 G. M. Sheldrick, *Acta Crystallogr. A,* 2015, **71**, 3–8.

S6 G. M. Sheldrick, *Acta Crystallogr. C,* 2015, **71**, 3–8.

S7 O. V. Dolomanov, L. J. Bourhis, R. J. Gildea, J. A. K. Howard and H. Puschmann, *J. Appl. Crystallogr.*, 2009, **42**, 339–341.
